# Characteristics of MRI lesions in AQP4 antibody-positive NMOSD, MOGAD, and multiple sclerosis: a systematic review and meta-analysis

**DOI:** 10.1007/s00415-025-13303-w

**Published:** 2025-08-07

**Authors:** Unnah Leitner, Sin Hong Chew, Jessica Blanch, Megha Viswanathan, Sasha Patil, Kayla Ward, Sandeep Bhuta, Ping Zhang, Jing Sun, Simon A. Broadley

**Affiliations:** 1 School of Medicine and Dentistry, Griffith University, Gold Coast Campus, Southport, QLD Australia; 2 Department of Neurology, Gold Coast University Hospital, Southport, QLD Australia; 3 Department of Radiology, Gold Coast University Hospital, Southport, QLD Australia; 4 Institute for Biomedicine and Glycomics, Griffith University, Gold Coast Campus, Southport, QLD Australia; 5 Data Science Institute, University of Technology Sydney, Sydney, NSW 2000 Australia; 6 Rural Health Research Institute, Charles Sturt University, Orange, NSW Australia

**Keywords:** Neuromyelitis optica, MOG antibody associated disease, Multiple sclerosis, Aquaporin-4 antibodies, Magnetic resonance imaging, Network meta-analysis

## Abstract

**Background and objectives:**

Multiple sclerosis (MS), aquaporin-4 antibody-positive neuromyelitis optica spectrum disorder (AQP4-Ab + ve NMOSD), and myelin oligodendrocyte glycoprotein-associated disease (MOGAD) are demyelinating diseases with differing pathophysiological processes and treatments. The objective of this study was to compile a comprehensive list of MRI lesions, and to quantify the utility of these lesions in distinguishing between these conditions.

**Methods:**

We searched for articles comparing MRI lesion frequency in MS, AQP4-Ab + ve NMOSD, MOGAD and healthy controls. Bayesian network meta-analysis together with pairwise and pooled case-case comparison analyses to develop sensitivity, specificity, and positive predictive values were undertaken.

**Results:**

Sixty-six articles were reported on 2933 MS, 3296 AQP4-Ab + ve NMOSD, and 1559 MOGAD cases, and 561 healthy controls. MRI lesions associated with MS were: periventricular T2, subcortical white matter T2, Dawson's finger, U-fibre T2 lesion, posterior spinal column T2, inferior temporal T2, cortical T2, brain T1 hypointensity (black holes), peripheral spinal cord T2, pons T2, unilateral optic nerve T2 and brain gadolinium enhancing lesions. Optic chiasm T2, LETM, bright spotty spinal cord T2, area postrema T2, hypothalamic T2, spinal cord atrophy and optic tract T2 lesions were associated with AQP4-Ab + ve NMOSD. Conus medullaris T2, fluffy, perineural enhancement, peri-ependymal 3rd ventricle T2 and peri-ependymal 4th ventricle T2 lesions were associated with MOGAD.

**Discussion:**

This review identified MRI features supportive of a diagnosis of MS, NMOSD or MOGAD, and has clarified the diagnostic utility of various MRI lesion characteristics, to aid in future clinical decision-making and guide future approaches to research.

**Supplementary Information:**

The online version contains supplementary material available at 10.1007/s00415-025-13303-w.

## Introduction

Multiple sclerosis (MS), aquaporin-4 antibody positive neuromyelitis optica spectrum disorder (AQP4-Ab + ve NMOSD), and myelin oligodendrocyte glycoprotein antibody-associated disease (MOGAD) are three discrete autoimmune diseases of the central nervous system (CNS) that cause demyelination. Whilst no antibody target is known for MS, distinct antibodies have been identified for AQP4-Ab + ve NMOSD and MOGAD. AQP4 is a water channel found in the foot processes of astrocytes, and on ependymal cells within the CNS, facilitating water transport between the blood, brain, and cerebrospinal fluid (CSF) [1–3]. Under the 2017 diagnostic criteria, the diagnosis of AQP4-Ab + ve NMOSD does not require the presence of AQP4 antibodies; clinical and MRI criteria can be used to diagnose NMOSD in the absence of AQP4 antibodies [4], but here we will only consider AQP4 antibody-positive cases. The antibody target identified for MOGAD is myelin oligodendrocyte glycoprotein (MOG) [5]. MOG is located on the surface of oligodendrocytes and is implicated in the structural integrity of myelin and the stabilisation of microtubules. Its external location on the oligodendrocyte membrane facilitates its role as a target in the immune system response [5]. Cell-based assays are recommended for both AQP4 and MOG antibodies, with live cell-based assays yielding more accurate results than fixed cell-based assays for AQP4 and particularly for MOG antibodies [6–8].

MRI is a useful tool in the diagnosis of autoimmune demyelinating disorders that affect the central nervous system, with MRI features being identified within the individual diagnostic criteria for MS, AQP4-Ab + ve NMOSD and MOGAD [4, 9, 10]. Whilst lesion examples have been identified in the diagnostic criteria for these conditions, there have been relatively few attempts to quantify differences in lesion frequencies between these three diseases. Globally, there also remains a lack of widespread standardised definitions for demyelinating lesions and their characteristics. There have been efforts to compile and standardise definitions [11], but further standardisation is necessary to optimise accuracy for future MRI studies. The purpose of this systematic review and meta-analysis was to explore the current literature for frequencies of MRI lesions and their characteristics, compared between AQP4-Ab + ve NMOSD, MOGAD and MS, and where possible, healthy controls (HC). These data have been analysed using network meta-analysis and a direct comparison meta-analysis to determine the level of association between the three conditions. We determined the following hypotheses: (1) some lesions will almost exclusively be seen in just one condition; (2) some lesions will be more common in one or more conditions; (3) some lesions would be seen in approximately equal numbers between the three conditions, as well as potentially in healthy controls (‘background noise’).

## Methods

### Search strategy

This systematic review and meta-analysis followed the Preferred Reporting Items for Systematic reviews and Meta-Analyses (PRISMA) guideline [12, 13]. To develop a comprehensive search strategy, the Population, Intervention, Control and Outcome (PICO) principles were used [14]. Population was adults or children (as long as specified) of any age. There was no intervention/exposure; therefore, a diagnosis of AQP4-Ab + ve NMOSD and MOGAD was used as the case comparator groups. The comparator group was MS, or where available, healthy controls. Outcome variables were MRI features—as defined by location, shape, defining characteristics or enhancement pattern, or a combination of these.

The finalised search strategy can be found in Supplementary Table 1. The search strategy was based on MRI, any combination of the three diagnoses (AQP4-Ab + ve NMOSD, MOGAD and MS) and healthy controls, MRI features, and study type. The review protocol was registered with PROSPERO (CRD42023347857).

### Inclusion–exclusion criteria

Studies were included if they met the following inclusion criteria: (1) summary demographics of participants were stated; (2) diagnosis of AQP4-Ab + ve NMOSD or MOGAD was made using AQP4 or MOG cell-based assays, with all participants positive for respective antibodies or data for this subgroup being available; (3) either no control population, or a comparison population consisting of people with MS or healthy controls; (4) primary outcomes assessed MRI features and reported on these; (5) published in a peer-reviewed journal between January 2000 and July 2024; (6) published in English and (7) cohort study or case–control study.

Exclusion criteria were: (1) fewer than 20 participants at baseline; (2) diagnosis of AQP4-Ab + ve NMOSD via outdated or unvalidated criteria; (3) seronegative AQP4-Ab + ve NMOSD; (4) MRI data that do not fit into the scope of lesion characterisation (e.g. volumetric studies) and (5) magnetic resonance spectroscopy or functional MRI studies.

Articles using advanced MRI techniques which require post-processing such as quantified volumetric analyses were excluded, as the scope of this review aimed to assess MRI lesions and characteristics that can be identified with routine MRI. Some simple volumetric changes evident on routine imaging (e.g. spinal cord atrophy/swelling) were included.

### Data extraction

The search was completed and screened by two independent reviewers (U.L, SH.C). Any conflicts in screening were resolved through discussion. Databases used to complete the search were PubMed, Scopus and Embase. All citations and abstracts were downloaded and imported into Covidence^®^ (Veritas Health Innovation Ltd, Melbourne, Australia) for review [15]. Duplicates were removed, and all articles underwent title and abstract screening, with disagreements resolved through discussion. Articles that included the same or similar authors were screened to ensure that the recruitment dates or study centres differed, to avoid doubling up of cohorts. Where overlap of included cases was identified, only the most recent and complete analysis was included. The remaining articles were then screened as full text articles against the inclusion and exclusion criteria, and a final list for data extraction determined. Bibliographies of prior reviews and the included articles were screened, and any additional relevant references were screened in the same way and added to the final list.

Data were extracted by independent reviewers (U.L, SH.C, J.B, M.V) and compared for disparities, which were subsequently resolved. The following data were extracted from the included articles: study type, location, participant demographics (age, sex), disease characteristics including disease duration and expanded disability status scale, number of participants, populations assessed, diagnostic methods, MRI protocols and lesion classification, MRI features of each population (including brain, spine and orbits) with frequency of each lesion or feature. The Newcastle Ottawa scale (NOS) was used for quality assessment of the studies [16].

### Statistical analysis

#### Network meta-analysis

The network meta-analysis was conducted using the STATA^®^ v17.0 software package (StataCorp LLC, College Station, TX, US). Features reported by > 10 studies were included in the network meta-analysis to ensure adequate statistical power. Demographic variables were used as baseline variables and were adjusted for covariates during regression analyses.

First, pairwise meta-analyses were performed. Random-effects model meta-analyses were conducted to obtain effect sizes for outcomes and dichotomous outcomes presented as risk ratio (RR) and continuous outcomes as standardised mean difference with 95% confidence interval (CI) separately.

Second, random-effects network meta-analyses of dichotomous outcomes using a Bayesian framework with Markov chain Monte Carlo methods were used [17]. All analyses were run on 4 chains with 20,000 iterations per chain, including a burn-in period of 1000 runs. Surface under the cumulative ranking curve (SUCRA) probabilities were used to rank the various populations for an outcome [18]. Principal coordinate analysis (PCoA) based on SUCRA values to display the overall ranking distribution across the three demyelinating disease parameters via dimension reduction was undertaken. Inconsistency between direct and indirect evidence, which could lead to inconsistency of the model, was assessed by the node splitting method and inconsistency plot performed. Gelman–Rubin plots were generated to assess convergence and variance in the chosen studies [19]. A two-sided *P* value of < 0.05 was regarded as statistically significant.

Covariate data presented as median (range or interquartile range) were converted to mean (standard deviation) using previously published methods [20] to permit inclusion as covariates, with the exception of EDSS which was instead presented according to its originally published format, as normal distribution could not be assumed.

#### Meta-analysis of MRI feature frequencies

To assess less commonly reported MRI features, all extracted frequency-related variables were individually meta-analysed, to generate a pooled proportion for each population (MS, AQP4-Ab + ve NMOSD and MOGAD). Extracted variables with < 2 reporting studies were not able to be meta-analysed and were, therefore, not included within the remaining statistical analyses. These meta-analyses were completed in R using JASP (2024), with random-effects models and continuity correction, using the restricted maximum likelihood method. Pooled proportions were used to calculate an estimated ‘n’ using the total ‘N’ for each MRI feature from the included studies. From these values, odds ratios, sensitivity, and specificity were calculated for each comparison, with 95% CI.

#### Pairwise comparisons

Pairwise comparisons were made between each combination of the three diseases. The odds ratios from all extracted variables with pooled proportions were ranked by the size of the odds ratio and were plotted for visual representation with 95% CI. Significant odds ratios favouring a particular disease were colour coded (purple = AQP4-Ab + ve NMOSD, green = MOGAD, red = MS).

#### Pooled case-case comparison

The pooled case-case comparison calculated an odds ratio from the pooled proportion of the index group against the combined proportions of the other two groups. This analysis design has been supported for the study of rare diseases [21, 22]. A pooled case-case comparison was conducted where at least one significant pairwise analysis result, and the pooled proportion of the index population was > 1.5 times larger (or smaller) than both other cohorts. This was based on the notion that, for variables fulfilling these criteria, lesion frequencies in the comparison groups were more likely to reflect either background healthy control frequencies and/or non-specific inflammatory changes.

#### Sensitivity, specificity, and PPV

Sensitivity, specificity, and positive predictive value (PPV) were calculated for each of the variables which underwent pooled case-case comparisons. To calculate PPV, we utilised two separate estimates for the relative frequency of the three conditions to reflect typical distributions from populations with largely European ancestry and East Asian ancestry. The following estimates were used based on relative differences in population prevalence figures in both regions [23–26]: Western Ancestry MS = 97%, AQP4-Ab + ve NMOSD = 1% and MOGAD = 2%; East Asian Ancestry MS = 33%, AQP4-Ab + ve NMOSD = 33% and MOGAD = 33%. Variables included in pooled case-case comparisons and those significant in the network meta-analysis were ranked according to their PPV.

#### Meta-regression

A meta-regression was performed on subcortical white matter T2 lesions, as this was a feature hypothesised to be either related to comorbid conditions such as migraine (which is more common in females) or vascular disease (age-related).

## Results

From an initial search of 2359 articles, 66 articles were selected for this review, which are summarised in Supplementary Table 2. Total populations were as follows: 2933 MS, 3296 AQP4-Ab + ve NMOSD, 1559 MOGAD and 561 healthy controls. The disposition of articles assessed is shown in Supplementary Fig. 1. Populations were analysed from 27 countries across the Americas, Europe, Asia and Australasia. Twenty-nine papers included orbital MRI analysis, forty-seven papers included spinal MRI analysis, and thirty-eight papers included brain MRI analysis. Thirty-four papers (52%) looked specifically at MRIs obtained during an acute attack or relapse, four papers (6%) specifically assessed MRIs obtained in the chronic phase, and thirteen papers (20%) included MRIs obtained at any time. Fifteen papers (22%) did not clearly specify the phase of disease under investigation. Thirty-three papers (50%) had reviewers who were blinded to disease phenotype during the MRI analysis process.

### Network meta-analysis

**Fig. 1:**
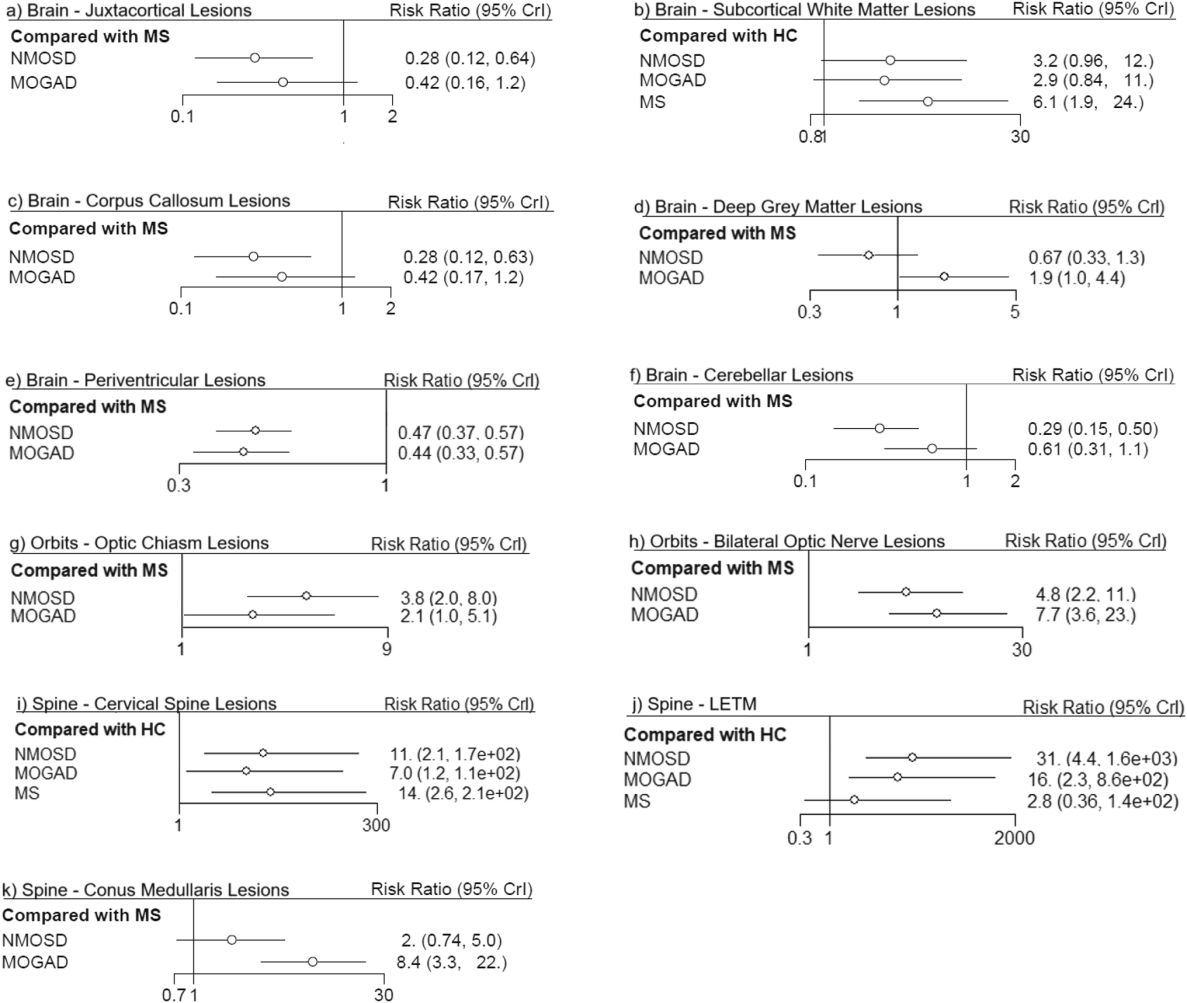
Forest plots from network meta-analysis comparing MS, NMOSD, MOGAD ± HC

Supplementary Fig. 2 shows the eligible comparison networks between each of the populations for variables which underwent network meta-analysis. The number of studies and sample sizes for each MRI feature are given in Supplementary Table 3. Forest plots from the network meta-analysis are shown in Fig. 1, with a visualisation of the rank probability for each of these MRI features given in Supplementary Fig. 3. The network meta-analysis demonstrated that, whilst only periventricular lesions were significantly associated with MS, juxtacortical, corpus callosum, cerebellar and subcortical T2 lesions were all more common in MS. Conus medullaris lesions were significantly associated with MOGAD, whilst optic chiasm, bilateral optic nerve T2 lesions, and longitudinally extensive transverse myelitis (LETM) were common in both AQP4-Ab + ve NMOSD and MOGAD.

### Pairwise comparisons

**Fig. 2:**
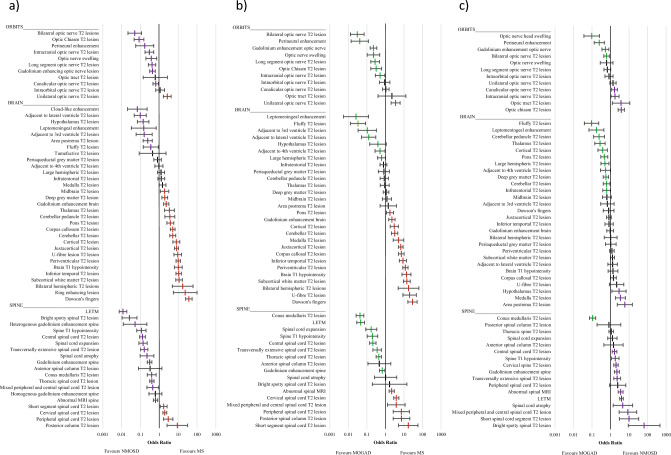
Odds ratio plots (with 95% confidence intervals) for all extracted variables in individual comparisons; **a** MS vs NMOSD; **b** MS vs MOGAD; **c** NMOSD vs MOGAD

Odds ratios with 95% CI for pairwise comparisons (MS vs AQP4-Ab + ve NMOSD; MS vs MOGAD; AQP4-Ab + ve NMOSD vs MOGAD) of all extracted lesions are shown in Fig. 2. Supplementary Table 4 displays the frequencies, sensitivity, specificity and odds ratios with 95% CI for all pairwise population comparisons between MS, AQP4-Ab + ve NMOSD and MOGAD. Spinal lesions of most types were more commonly associated with a diagnosis of AQP4-Ab + ve NMOSD compared to either MS or MOGAD, whereas peri-ependymal lesions of various locations, and specific gadolinium (Gd) enhancement patterns were more frequently associated with MOGAD. Cerebral lesions of the white and cortical grey matter were more commonly seen in MS.

### Pooled case-case comparisons

**Fig. 3:**
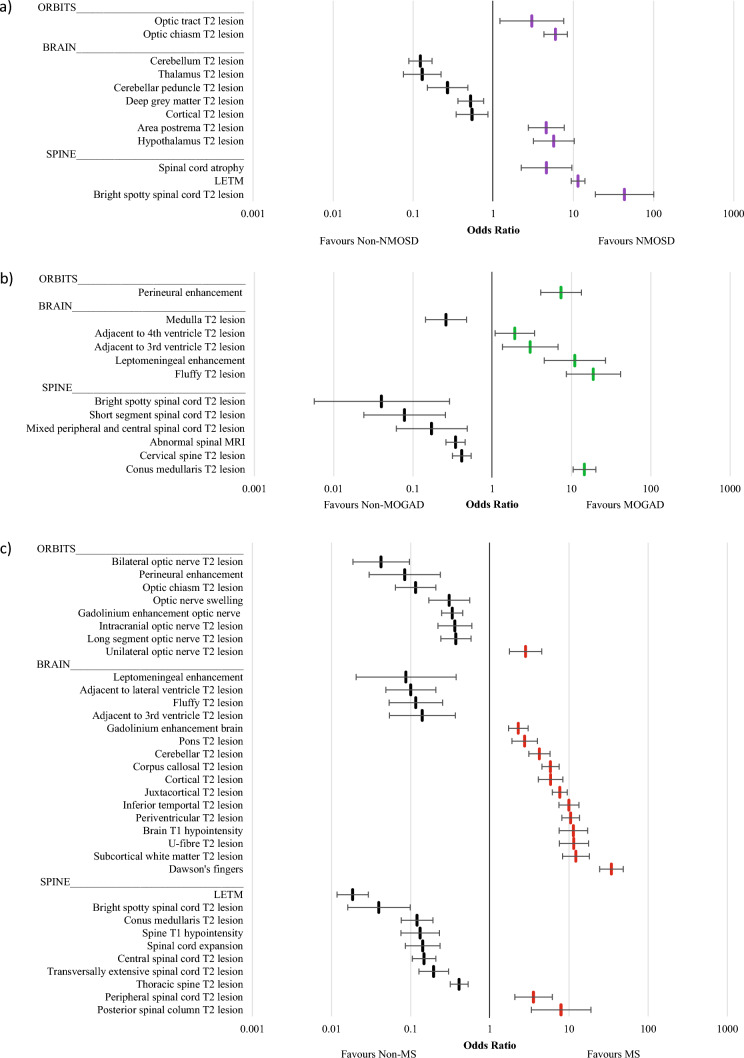
Odds ratio plots (with 95% confidence intervals) of eligible variables for combination cohort analysis **a** NMOSD vs non-NMOSD **b** MOGAD vs non-MOGAD **c** MS vs non-MS

Odds ratios for MRI features identified as being distinctly more common in one condition as compared to the other two conditions are shown in Fig. 3. Frequencies and odds ratios are displayed in Supplementary Tables 5–7. MS had the largest variety of associated lesions, often related to the brainstem and cerebrum. The strongest associated features for MS were Dawson’s fingers (OR 34.11; 95%CI 24.16–48.14), subcortical white matter T2 lesions (OR 12.16; 95%CI 8.22–17.99), and U-fibre T2 lesions (OR 11.44; 95%CI 7.46–17.52). Both AQP4-Ab + ve NMOSD and MOGAD had lower numbers of associated lesions in the case-case comparison analysis. The strongest associations with AQP4-Ab + ve NMOSD were LETM (OR 11.38; 95%CI 9.33–13.87), optic chiasm T2 lesions (OR 5.95; 95%CI 4.25–8.33), and hypothalamic T2 lesions (OR 5.65; 95%CI 3.13–10.21). For MOGAD, the strongest associated features were fluffy T2 lesions (OR 18.78; 95%CI 8.51–41.46) and conus medullaris T2 lesions (OR 14.50; 95%CI 10.39–20.23).

### Sensitivity, specificity and PPV

Sensitivity, specificity and PPV for all variables which underwent pooled case-case comparisons were calculated and are displayed in Table 1. Typical MRI features of MS have been previously defined as periventricular lesions, cortical/juxtacortical lesions, infratentorial lesions, gadolinium enhancing lesions or cervical/thoracic spinal cord lesions [10, 27, 28]. Of these, periventricular, cortical and gadolinium enhancing brain lesions were associated with MS (Fig. 3). Juxtacortical lesions were found to be suggestive of MS in the pooled case-case comparison analysis, but there was an unclear distinction between MS and MOGAD within the network meta-analysis (Fig. 1). Dawson’s fingers are a well-known feature for MS [29], and were supported by this review, yielding the highest PPV of the MS group of 0.998 in Western populations (Table 1). U-fibre lesions were also found to be strongly associated with MS with a PPV of 0.996 in the West.

**Table 1:** Positive predictive values for disease-associated lesions

				PPV	
Cohort	Lesion	Sens	Spec	Western	Far east
MS	Dawson’s fingers	0.600 (0.560–0.638)	0.958 (0.944–0.969)	0.998 (0.997–0.998)	0.875 (0.839–0.905)
	U-fibre T2 lesion	0.379 (0.325–0.435)	0.949 (0.929–0.965)	0.996 (0.994–0.997)	0.787 (0.718–0.842)
	Posterior spinal column T2 lesion	0.373 (0.258–0.499)	0.930 (0.866–0.969)	0.994 (0.099–0.997)	0.724 (0.556–0.846)
	Inferior temporal T2 lesion	0.541 (0.499–0.582)	0.894 (0.869–0.916)	0.994 (0.992–0.995)	0.715 (0.666–0.759)
	Cortical T2 lesion	0.440 (0.386–0.495)	0.881 (0.848–0.909)	0.992 (0.989–0.994)	0.646 (0.580–0.706)
	*Cerebellar T2 lesion*	0.279 (0.232–0.329)	0.916 (0.898–0.931)	0.991 (0.998–0.993)	0.620 (0.559–0.677)
	Brain T1 hypointensity	0.781 (0.726–0.830)	0.760 (0.702–0.813)	0.991 (0.988–0.993)	0.616 (0.560–0.670)
	*Corpus callosal T2 lesion*	0.560 (0.514–0.605)	0.820 (0.793–0.846)	0.990 (0.988–0.992)	0.605 (0.565–0.644)
	*Juxtacortical T2 lesion*	0.719 (0.684–0.753)	0.748 (0.721–0.774)	0.989 (0.988–0.990)	0.584 (0.556–0.612)
	Peripheral spinal cord T2 lesion	0.436 (0.334–0.542)	0.821 (0.759–0.872)	0.987 (0.982–0.991)	0.545 (0.451–0.636)
	**Periventricular T2 lesion**	0.869 (0.841–0.894)	0.611 (0.580–0.641)	0.986 (0.985–0.987)	0.524 (0.503–0.544)
	Pons T2 lesion	0.342 (0.275–0.415)	0.840 (0.808–0.868)	0.986 (0.981–0.989)	0.513 (0.446–0.580)
	**Subcortical white matter T2 lesion**	0.920 (0.888–0.946)	0.513 (0.482–0.543)	0.984 (0.983–0.985)	0.482 (0.465–0.499)
	Unilateral optic nerve T2 lesion	0.635 (0.524–0.737)	0.617 (0.576–0.657)	0.982 (0.978–0.985)	0.450 (0.403–0.497)
	Gadolinium enhancement brain	0.520 (0.468–0.572)	0.677 (0.630–0.721)	0.981 (0.978–0.984)	0.442 (0.401–0.484)
NMOSD	Bright spotty spinal cord T2 lesion	0.430 (0.371–0.491)	0.988 (0.937–0.999)	0.272 (0.050–0.725)	0.948 (0.721–0.992)
	Area postrema T2 lesion	0.209 (0.161–0.265)	0.945 (0.919–0.965)	0.037 (0.024–0.058)	0.652 (0.541–0.749)
	Hypothalamic T2 lesion	0.181 (0.136–0.233)	0.962 (0.940–0.978)	0.046 (0.027–0.077)	0.703 (0.570–0.804)
	**Optic chiasm T2 lesion**	0.269 (0.239–0.302)	0.942 (0.923–0.957)	0.045 (0.033–0.059)	0.694 (0.627–0.755)
	Spinal cord atrophy	0.189 (0.136–0.253)	0.951 (0.913–0.977)	0.038 (0.020–0.072)	0.658 (0.495–0.790)
	**LETM**	0.730 (0.703–0.756)	0.808 (0.784–0.830)	0.037 (0.033–0.042)	0.652 (0.623–0.679)
	Optic tract T2 lesion	0.100 (0.064–0.146)	0.964 (0.824–0.987)	0.028 (0.012–0.064)	0.580 (0.365–0.768)
MOGAD	**Conus medullaris T2 lesion**	0.330 (0.285–0.380)	0.970 (0.958–0.975)	0.183 (0.133–0.214)	0.844 (0.788–0.868)
	Fluffy lesion	0.755 (0.611–0.867)	0.859 (0.794–0.910)	0.099 (0.067–0.143)	0.725 (0.634–0.800)
	Perineural enhancement	0.465 (0.357–0.576)	0.894 (0.848–0.930)	0.082 (0.055–0.122)	0.684 (0.583–0.770)
	Peri-ependymal 3rd ventricle T2 lesion	0.158 (0.075–0.279)	0.941 (0.915–0.961)	0.052 (0.026–0.099)	0.569 (0.395–0.727)
	*Bilateral optic nerve T2 lesions*	0.401 (0.333–0.472)	0.816 (0.787–0.843)	0.043 (0.034–0.053)	0.518 (0.462–0.574)
	Peri-ependymal 4th ventricle T2 lesion	0.318 (0.209–0.444)	0.804 (0.762–0.842)	0.032 (0.022–0.047)	0.444 (0.348–0.545)
NMOSD/ MOGAD	**LETM**	0.632 (0.608–0.656)	0.969 (0.953–0.981)	0.390 (0.293–0.496)	0.976 (0.963–0.984)
	Peri-ependymal lateral ventricle T2 lesion	0.232 (0.197–0.270)	0.971 (0.943–0.987)	0.197 (0.109–0.330)	0.939 (0.884–0.969)
	T1 hypointensity spine	0.509 (0.455–0.563)	0.881 (0.813–0.930)	0.116 (0.076–0.174)	0.892 (0.838–0.930)
	Spinal cord expansion	0.518 (0.457–0.578)	0.868 (0.807–0.916)	0.108 (0.075–0.154)	0.884 (0.836–0.920)
	Central spinal cord T2 lesion	0.564 (0.531–0.597)	0.840 (0.792–0.881)	0.099 (0.077–0.126)	0.873 (0.839–0.900)
	Whole spinal cord T2 lesion	0.630 (0.584–0.674)	0.752 (0.671–0.822)	0.073 (0.055–0.096)	0.831 (0.785–0.869)
	Long segment optic nerve T2 lesion	0.413 (0.369–0.459)	0.793 (0.718–0.856)	0.058 (0.042–0.080)	0.795 (0.735–0.844)
	Optic nerve swelling	0.613 (0.516–0.704)	0.675 (0.563–0.774)	0.055 (0.040–0.076)	0.785 (0.722–0.838)
	Thoracic spine T2 lesion	0.475 (0.445–0.506)	0.731 (0.682–0.776)	0.052 (0.044–0.061)	0.774 (0.741–0.804)
	Gadolinium enhancement optic nerve	0.619 (0.569–0.668)	0.649 (0.594–0.701)	0.052 (0.044–0.061)	0.774 (0.743–0.802)

Lesion types listed in **bold** were significantly associated with the disease in both the network meta-analysis and disease vs. all-others analysis. Lesion types listed in *italics* were significantly associated with the disease in either the network meta-analysis or the disease vs. all other analysis, but not both. Lesion types in regular text were significantly associated, but analysis was only completed for this variable in the disease vs. all-others analysis

The 2015 international panel for NMO diagnosis (IPND) criteria for AQP4-Ab + ve NMOSD included MRI requirements for the diagnosis of AQP4-Ab + ve NMOSD, in cases without AQP4-IgG, or with an unknown AQP4-IgG status [4]. These included optic nerve T2 lesions (including optic chiasm), LETM, lesions involving the area postrema and peri-ependymal brainstem lesions. All these features, except for peri-ependymal brainstem lesions, were found to be associated in the current data (Fig. 3). Importantly, our analysis found that LETM was the only variable to appear in two sections of Table 1—implying that LETM is still suggestive of both AQP4-Ab + ve NMOSD and MOGAD but is distinctly more common in AQP4-Ab + ve NMOSD than MOGAD. MRI features which are thought to be characteristic or highly suggestive of NMOSD were outlined in the elaborated NMOSD radiological features of the 2015 IPND criteria [4]. Features described as characteristic or highly suggestive of NMOSD, which were also found to be significantly associated with AQP4-Ab + ve NMOSD in this review, included optic tract T2 lesions, hypothalamic T2 lesions and spinal cord atrophy.

The 2023 diagnostic criteria for MOGAD included a number of MRI features to support a diagnosis of MOGAD [9]. Our findings supported the consensus, that of these recognised features, bilateral optic nerve involvement, perineural optic nerve Gd-enhancement, conus medullaris lesions, leptomeningeal Gd-enhancement and fluffy T2 lesions may be reliable MRI features for the diagnosis of MOGAD (Fig. 3).

### Meta-regression analysis

Meta-regression analysis for subcortical white matter T2 lesions is shown in Supplementary Table 9 and shows that these lesions were significantly lower in AQP4-Ab + ve NMOSD, MOGAD and HC compared to MS, and that this relationship was not significantly influenced by sex or age.

## Discussion

This review analysed the specificity/sensitivity and PPV of various MRI characteristics when directly comparing MS, AQP4-Ab + ve NMOSD and MOGAD, identified MRI features that may be specific for MS, AQP4-Ab + ve NMOSD and MOGAD, and may prove useful in aiding diagnostic suspicion for diagnosis of these diseases. Some of these features found in this review have already been identified and utilised in consensus-based diagnostic criteria, as noted above. However, there were some features for which this study raises questions over their diagnostic utility or have not been previously mentioned as being diagnostically useful.

Infratentorial lesions are stated in the diagnostic criteria as typical MS features (10, 28). Some of these locations were supported by our results, particularly pontine and cerebellar lesions. However, caution should be used in utilising the peri-ependymal lesions of the 4th ventricle, as this was found to be most specific to MOGAD. Other infratentorial lesions—cerebellar peduncles and medulla—were found to be non-specific. A selection of MRI features not mentioned in diagnostic criteria were identified within our review to support an MS diagnosis (see Table 1). The strongest example of this was posterior spinal column lesions, with a PPV of 0.994 in Western populations. Other distinct features, such as T1 brain hypointensities (black holes) and unilateral optic nerve T2 lesions, yielded high PPVs in the West (0.991 and 0.982 respectively) and therefore may be valuable diagnostic features.

Juxtacortical lesions and U-fibre lesions were found to be reliable features of MS. However, these two lesion types had unclear definitions, with some papers only referring to one [30–33], some separating them [11, 34–36], and others indicating that U-fibre lesions are a subtype of juxtacortical lesions [37–41]. There was heterogeneity in the definitions used where these were given. Juxtacortical lesions were defined in the MS diagnostic criteria revisions in 2017 [10], as lesions which abut the cortex without any intervening white matter. As both juxtacortical and U-fibre lesions were significantly associated with MS, even with discrepancies in definitions, it is likely that in combination, these lesions would be significantly associated with MS.

Subcortical white matter lesions are difficult to interpret, as they are present in demyelinating diseases, vascular disease [42], migraine [43], and may be present in healthy or asymptomatic populations as an age-related phenomenon [44]. Our results suggested that subcortical white matter lesions were most strongly associated with MS. Due to the relatively small sample size of healthy control populations when analysing subcortical white matter lesions, caution should be taken regarding its frequency of occurrence. Interestingly, there was no significant difference found between AQP4-Ab + ve NMOSD, MOGAD and healthy control populations for subcortical white matter lesion frequency in the network meta-analysis, which suggests that subcortical white matter lesions are non-specific. The meta-regression found that age at time of study and sex did not appear to significantly account for the heterogeneity found in cortical white matter lesions between the groups. This suggests that non-specific subcortical white matter lesions are not explained by co-existing migraine and age-related vascular disease. However, over and above this ‘background noise’, subcortical white matter lesions are more common in MS, suggesting that they arise as a consequence of disease-related inflammation in this condition. Further targeted research is needed to investigate subcortical white matter lesions in various disease and control populations.

The 2015 IPND criteria [4] identified peri-ependymal lesions of either the 3rd or the 4th ventricle as neuroimaging features of AQP4-Ab + ve NMOSD. This review found that both these features were instead more strongly associated with a diagnosis of MOGAD—therefore their occurrence in AQP4-Ab + ve NMOSD cannot be supported by this review. This misclassification may have been due to earlier studies including seronegative AQP4-Ab + ve NMOSD cases prior to the widespread availability of MOG antibody testing. Inclusion of these seronegative cases (some of which may have therefore been MOGAD cases) may have led to a false association of these MRI features with AQP4-Ab + ve NMOSD. Lesions of the thalamus have similarly been identified as an MRI characteristic of AQP4-Ab + ve NMOSD [4]. However, this review found that these lesions were instead more suggestive of either MS or MOGAD. The current findings do support the relative specificity of bright spotty spinal cord lesions for AQP4-Ab + ve NMOSD [11, 45–50].

Deep grey matter lesions and lesions involving the cerebellar peduncle were noted within the proposed 2023 consensus criteria for MOGAD [9]. They were not found to be significantly associated in this review. Both features were useful for ruling out AQP4-Ab + ve NMOSD, but the distinction between MOGAD and MS was less clear.

This review suggests some additional MRI features that should be considered as supportive of a diagnosis of MOGAD. Peri-ependymal lesions adjacent to the 3rd or 4th ventricles, as stated previously, should be considered further for utility in the diagnosis of MOGAD. ‘Fluffy’ lesions were also associated with a diagnosis of MOGAD. This is a relatively novel MRI feature where, in the few studies assessing its occurrence, the authors utilised the same definition of “a poorly demarcated lesion” to characterise the feature [30, 35].

Some MRI features were identified in this study as being clearly associated with antibody-mediated disease but were not useful in distinguishing between AQP4-Ab + ve NMOSD and MOGAD (see Table 1). From this list, it is most important to highlight spinal cord swelling/expansion and lesions involving the central spinal cord on axial imaging.

Attention must be drawn to the fact that whilst this review identified several features that were associated with either an AQP4-Ab + ve NMOSD or MOGAD diagnosis, the PPV of these features were so low in the West that no combination of these features in clinical practice would achieve a PPV approaching 1, negating any ability for clinical certainty based on MRI features alone. PPVs were comparatively higher in the MS group, with all features in the MS section Table 1 achieving a PPV of > 90% in the West. In the Far East, PPVs which are of much higher diagnostic utility were evident, particularly in AQP4-Ab + ve NMOSD and MOGAD populations. The PPVs of MS features were substantially lower in the Far East compared with the West (albeit still high enough to create diagnostic suspicion).

This review and network meta-analysis collated a comprehensive list of MRI features of demyelinating disease and quantified their distributions and diagnostic utility from the existing literature. There were numerous strengths of this paper, including: the use of standardised guidelines for developing the search strategy; large overall sample size for many variables; comprehensive inclusion of MRI features; use of standardised lesion definitions wherever possible; inclusion of multiple countries, populations and centres; comprehensive approach with robust statistical analyses; and having a high proportion (50%) of included articles utilising blinded reviewers for MRI analysis.

The main limitations of this study included: lack of data for some features of interest, such as specific corpus callosal lesion features (> 1/2 length of corpus callosum, diffuse, heterogenous or oedematous lesions) noted to be specific to AQP4-Ab + ve NMOSD, ovoid, ring and open-ring enhancement patterns in brain indicated to be suggestive of MS, H-sign on axial spinal imaging, and extensive middle cerebellar peduncle lesions, suggested to be specific for MOGAD; heterogeneity across studies (MRI technique, magnet size, population, ethnicity, referral patterns, antibody assay methods); lack of standardised definitions for some MRI features; and lack of suitable controls for comparison (healthy controls, other demyelinating diseases including transverse myelitis and optic neuritis, and other common disease states such as migraine and vascular disease). The present analysis can only provide insight in relation to the comparison between MS, NMOSD and MOGAD, and is of no value in distinguishing other conditions, e.g. vascular disease.

The principal values and outcomes of this study were: to act as an aid for diagnosis of demyelinating disease in the acute setting prior to availability of antibody results which may influence acute treatment (e.g. plasma exchange); to aid confirmation of MS diagnosis, which by definition is antibody negative; and to assist with potential identification of true seronegative AQP4-related NMOSD and MOGAD.

Future research would benefit from use of prospective cohort designs (recruitment prior to antibody assay results) to avoid selection bias, inclusion of relevant control populations (including vascular disease, migraine, and healthy controls) to determine accurate specificity of lesions, inclusion of all relevant MRI features, and use of patient level data (such as in the PAMRINO study) [51].

## Conclusion

This network meta-analysis has helped to quantify the MRI feature distribution between MS, AQP4-Ab + ve NMOSD and MOGAD, and provide guidance for the diagnostic utility of various MRI characteristics for each of these diseases. Whilst many features were consistent with consensus-driven diagnostic criteria, there were some features whose utility in their respective criteria was questioned. In addition, this review highlighted some features which have not previously been identified in diagnostic criteria, which should warrant further targeted investigation. Overall, the MRI features typical of MS had good predictive value in both Western and Asian populations. The features associated with AQP4-Ab + ve NMOSD and MOGAD were of lower utility particularly in Western populations and this is likely to make diagnosis of seronegative disease purely on the basis of MRI features difficult in this setting. A combination of MRI and paraclinical findings (e.g. CSF findings, serum NfL and GFAP levels) may prove more helpful but it seems likely that antibody assays will continue to have a central role in the diagnosis of these disorders.

## Supplementary Information

Below is the link to the electronic supplementary material.

Supplementary file1 (PDF 7200 KB)

Supplementary file2 (PDF 890 KB)

Supplementary file3 (DOCX 33 KB)
