# Supplementary material for: Characteristics of MRI lesions in AQP4 antibody-positive NMOSD, MOGAD, and multiple sclerosis: a systematic review and meta-analysis
Source: J Neurol. 2025 Aug 7;272(9):560. doi: 10.1007/s00415-025-13303-w (PMC12331836; doi:10.1007/s00415-025-13303-w)
Supplement: Supplementary file 1 — Supplementary file1 (PDF 7200 KB) [file 415_2025_13303_MOESM1_ESM.pdf]

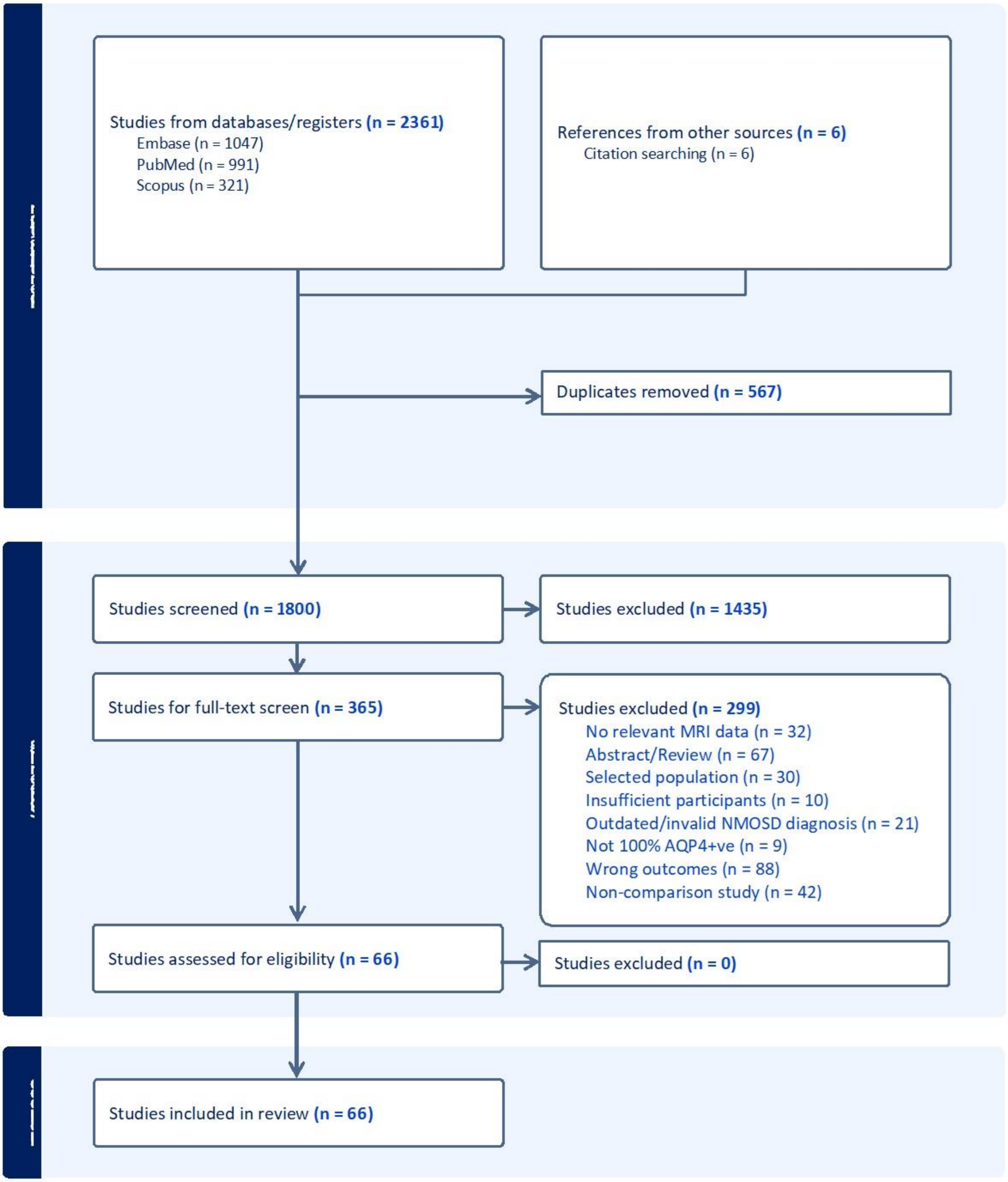

Supplementary Figure 1. PRISMA diagram for review.

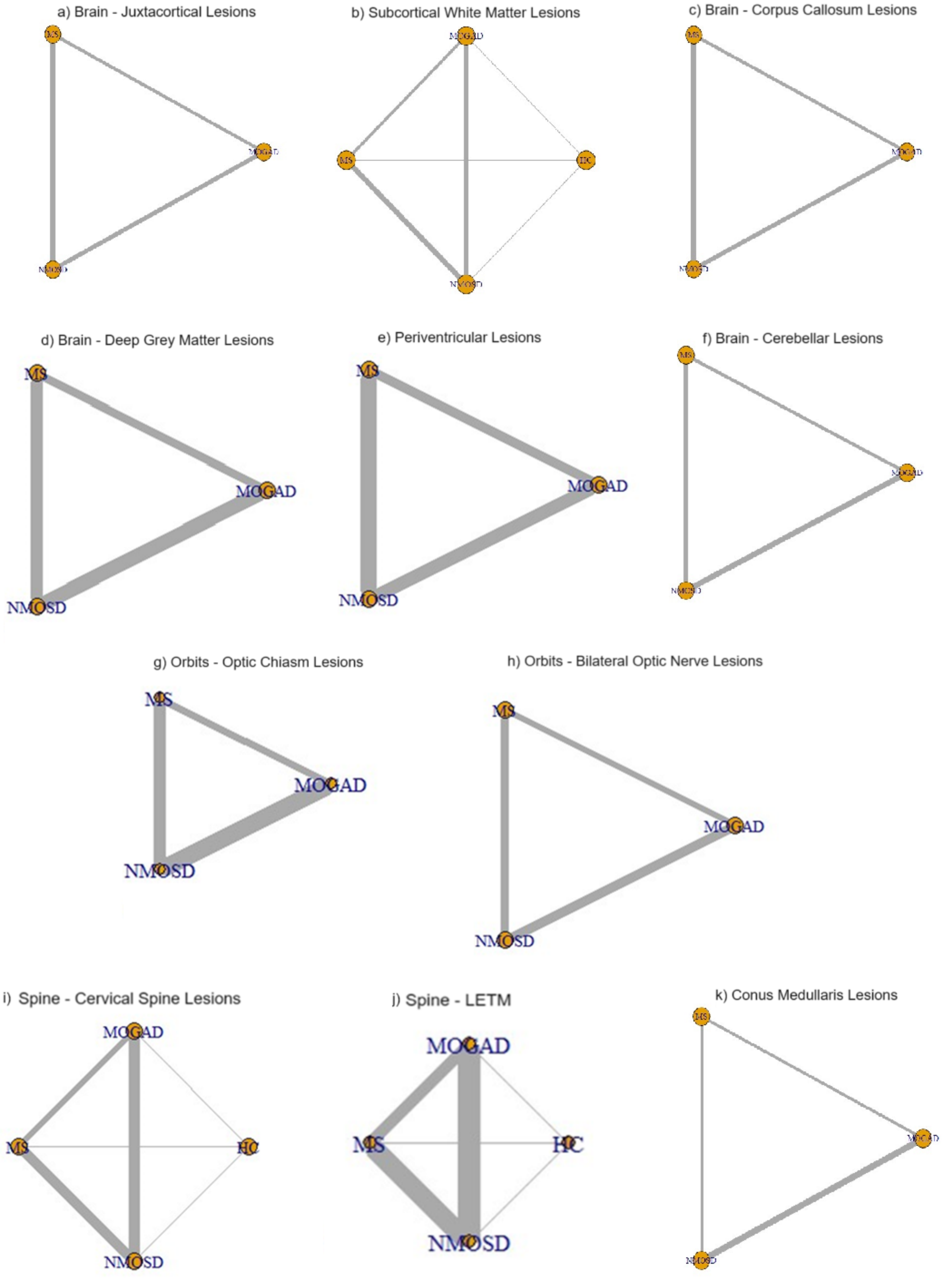

Supplementary figure 2. Network graphs for analysed variables. The width of the line is proportional to the number of studies.

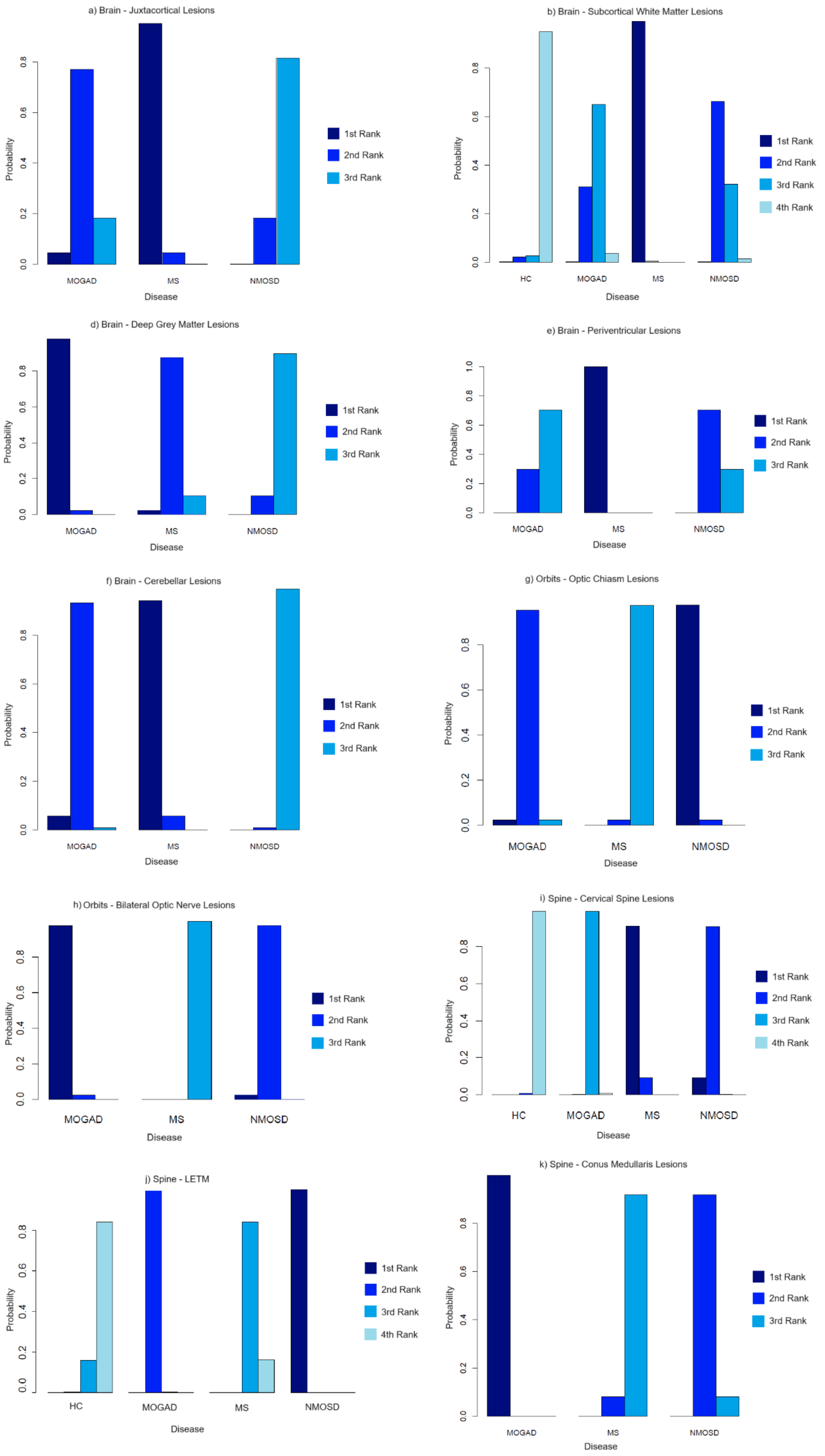

Supplementary figure 3. Rank probability plots for analysed variables.

Figure 5 a) **Brain – Juxtacortical Lesions**		
NMOSD		
-0.41 (-1.45, 0.51)	MOGAD	
-1.27 (-2.12, -0.45)	-0.87 (-1.81, 0.51)	MS

Figure 5 b) **Brain – Subcortical White Matter**		
NMOSD		
0.10 (-0.35, 0.57)	MOGAD	
-0.65 (-1.14, -0.27)	-0.75 (-1.35, -0.27)	MS
1.15 (-0.04, 2.47)	1.05 (-0.17, 2.38)	1.80 (0.62, 3.17)

Figure 5 c) **Brain – Corpus Callosum Lesions**		
NMOSD		
-0.41 (-1.44, 0.51)	MOGAD	
-1.27 (-2.11, -0.46)	-0.86 (-1.80, 0.18)	MS

Figure 5 d) **Brain - Deep Grey Matter Lesions**		
NMOSD		
-1.05 (-1.89, -0.45)	MOGAD	
-0.39 (-1.10, 0.26)	0.64 (0.02, 1.51)	MS

Figure 5 e) **Brain – Periventricular Lesions**		
NMOSD		
0.07 (-0.20, 0.36)	MOGAD	
-0.76 (-0.98, -0.56)	-0.83 (-1.13, -0.57)	MS

Figure 5 f) **Brain – Cerebellum Lesions**		
NMOSD		
-0.75 (-1.41, -0.15)	MOGAD	
-1.24 (-1.90, -0.69)	-0.49 (-1.17, 0.14)	MS

Figure 5 g) **Orbits - Optic Chiasm Lesions**		
NMOSD		
0.58 (0.02, 1.12)	MOGAD	
1.33 (0.69, 2.09)	0.76 (0.01, 1.64)	MS

Figure 5 h) **Orbits - Bilateral Optic Nerve Lesions**		
NMOSD		
-0.46 (-1.24, -0.01)	MOGAD	
1.56 (0.81, 2.42)	2.03 (1.27, 3.13)	MS

Figure 5 i) **Spine - Cervical Spine Lesions**			
NMOSD			
0.48 (0.18, 0.86)	MOGAD		
-0.20 (-0.52, 0.10)	-0.67 (-1.12, -0.34)	MS	
2.59 (0.92, 5.17)	2.11 (0.40, 4.68)	2.79 (1.12, 5.38)	HC

Figure 5 j) **Spine - LETM**			
NMOSD			
0.62 (0.32, 1.00)	MOGAD		
2.41 (1.98, 2.89)	1.78 (1.28, 2.30)	MS	
3.51 (1.45, 7.30)	2.88 (0.80, 6.69)	1.09 (-1.02, 4.93)	HC

Figure 5 k) **Spine – Conus Medullaris Lesion**		
NMOSD		
-1.44 (-2.08, -0.89)	MOGAD	
0.68 (-0.30, 1.62)	2.13 (1.21, 3.07)	MS

Supplementary figure 4. Pairwise meta-analysis between the groups. a-f) indicate primary outcome variables, g-k) indicate secondary outcome variables. Results are the effect estimate between groups (95% confidence interval). Significant results are highlighted in a darker yellow.

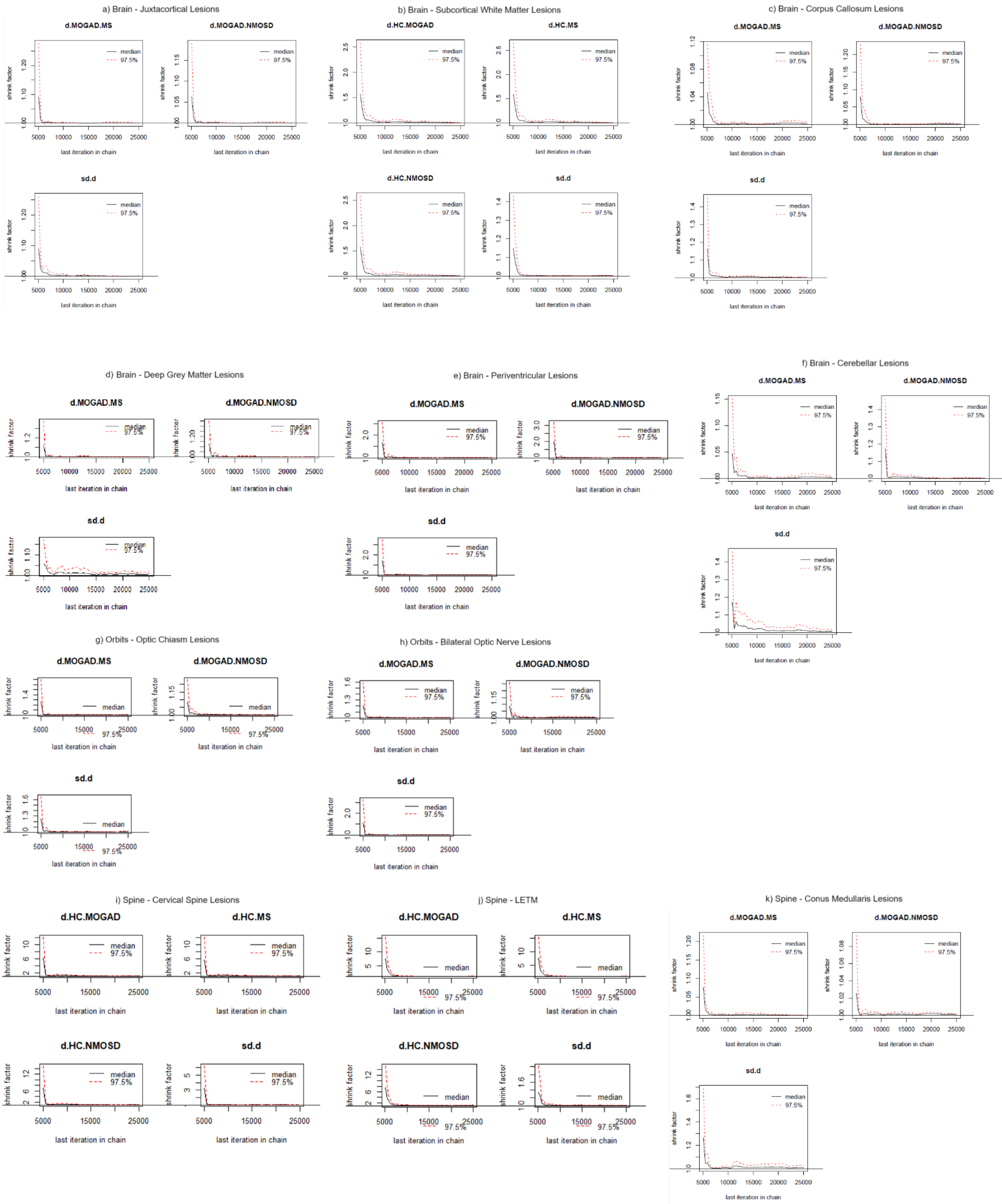

Supplementary figure 5. Gelman plots

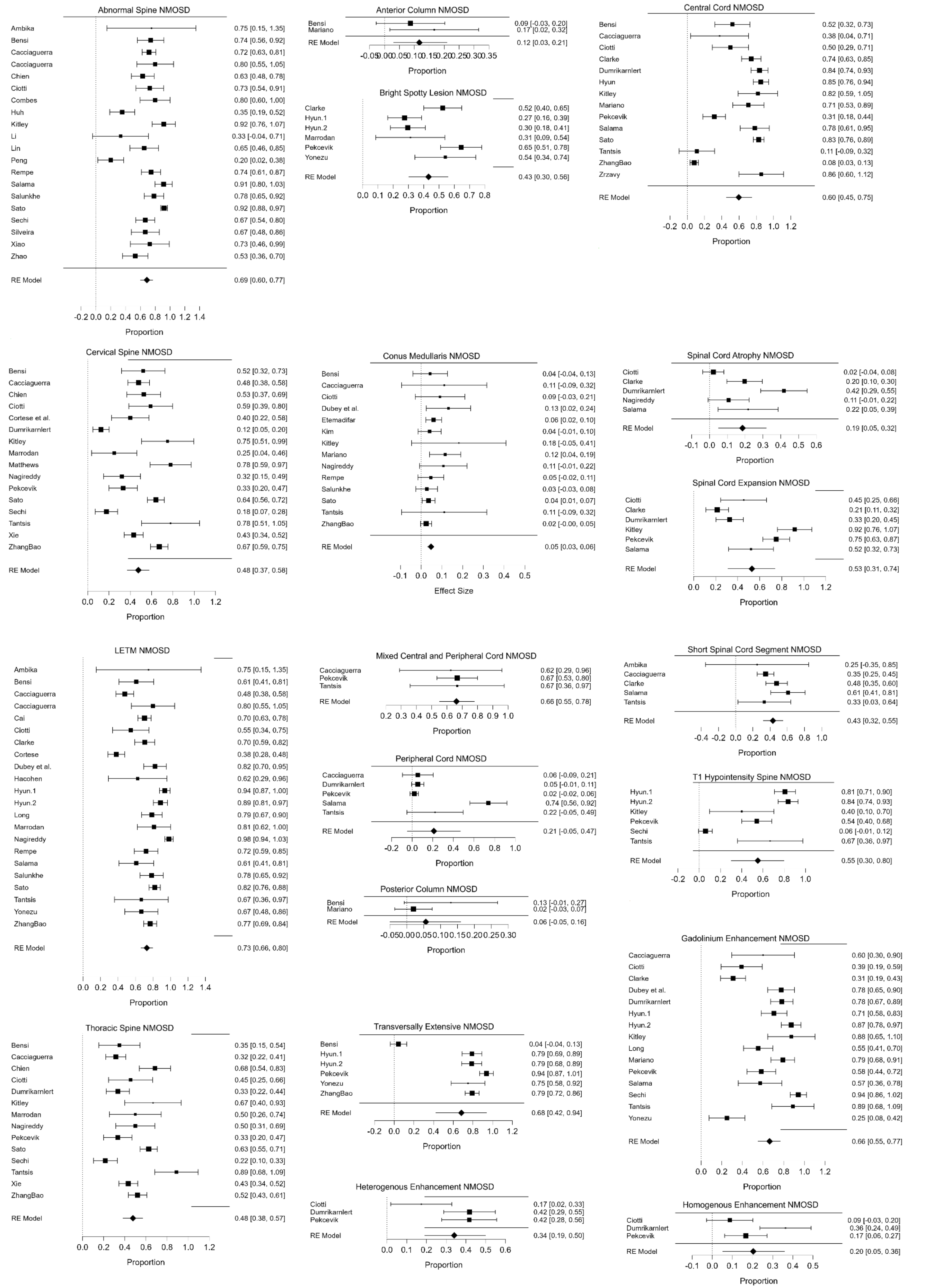

Figure 6 – Forest plots for individual meta-analyses: NMOSD Spine

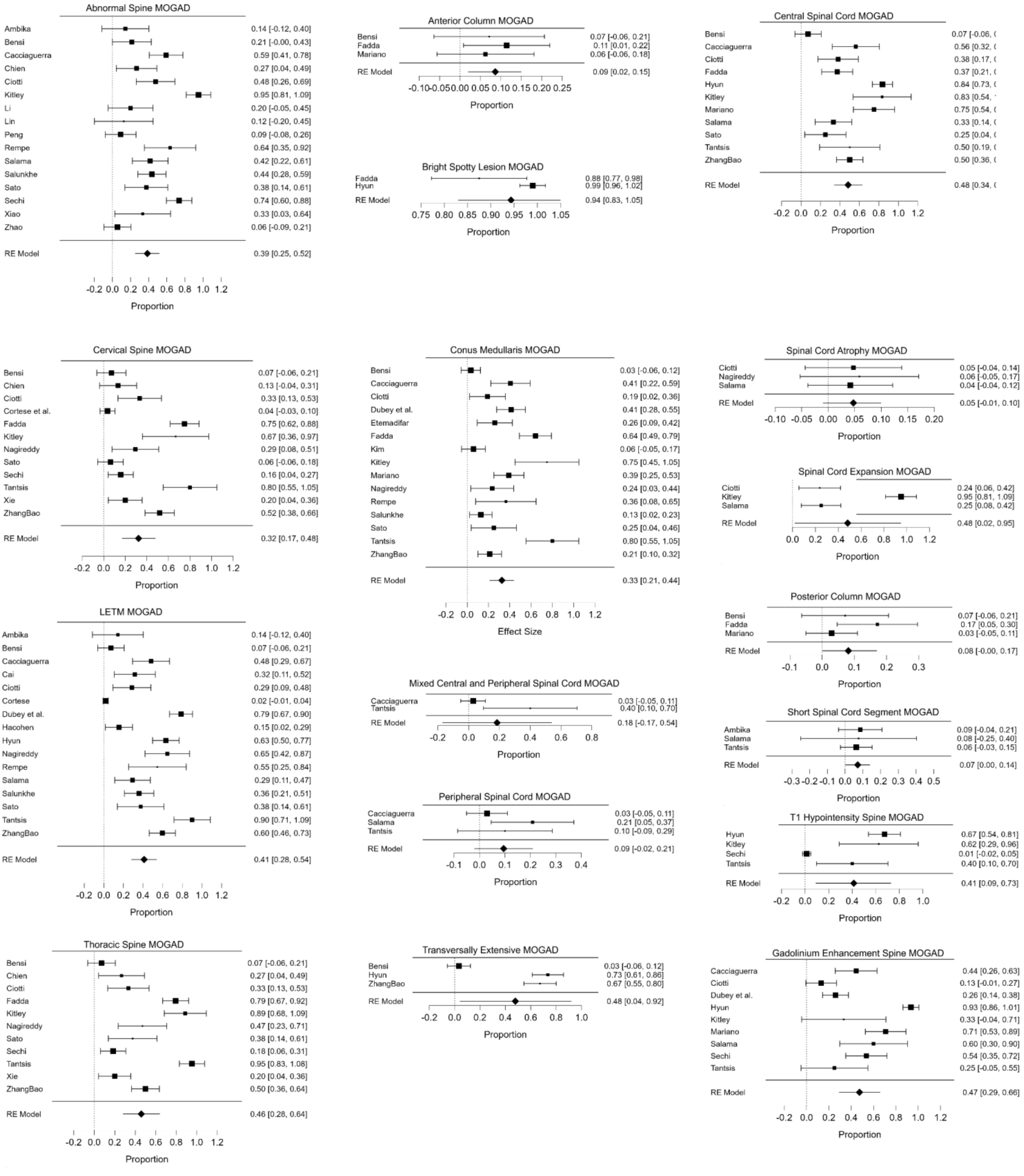

Supplementary figure 7 – Forest plots for individual meta-analyses: MOGAD Spine

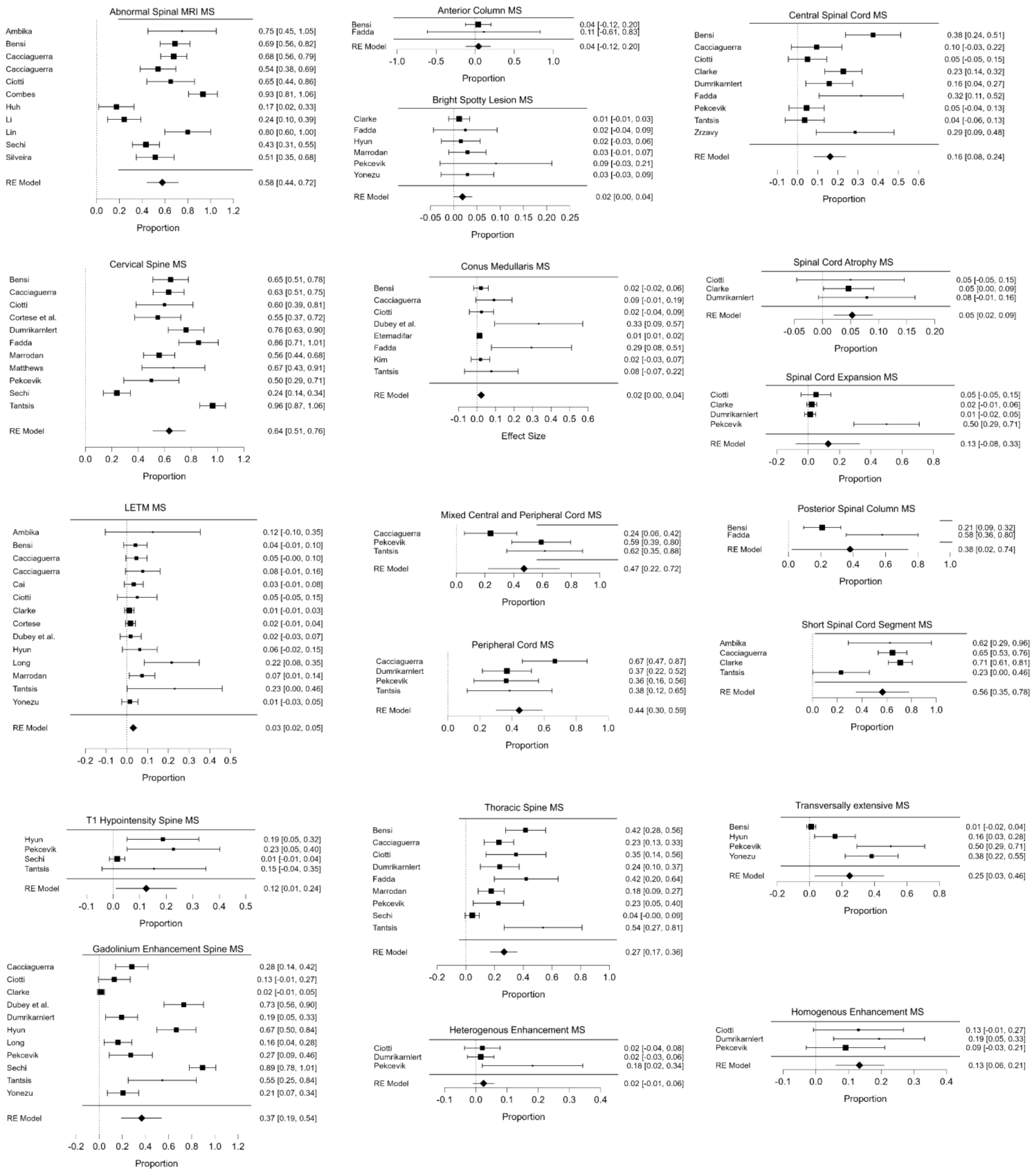

Supplementary figure 8 – Forest plots for individual meta-analyses: MS Spine

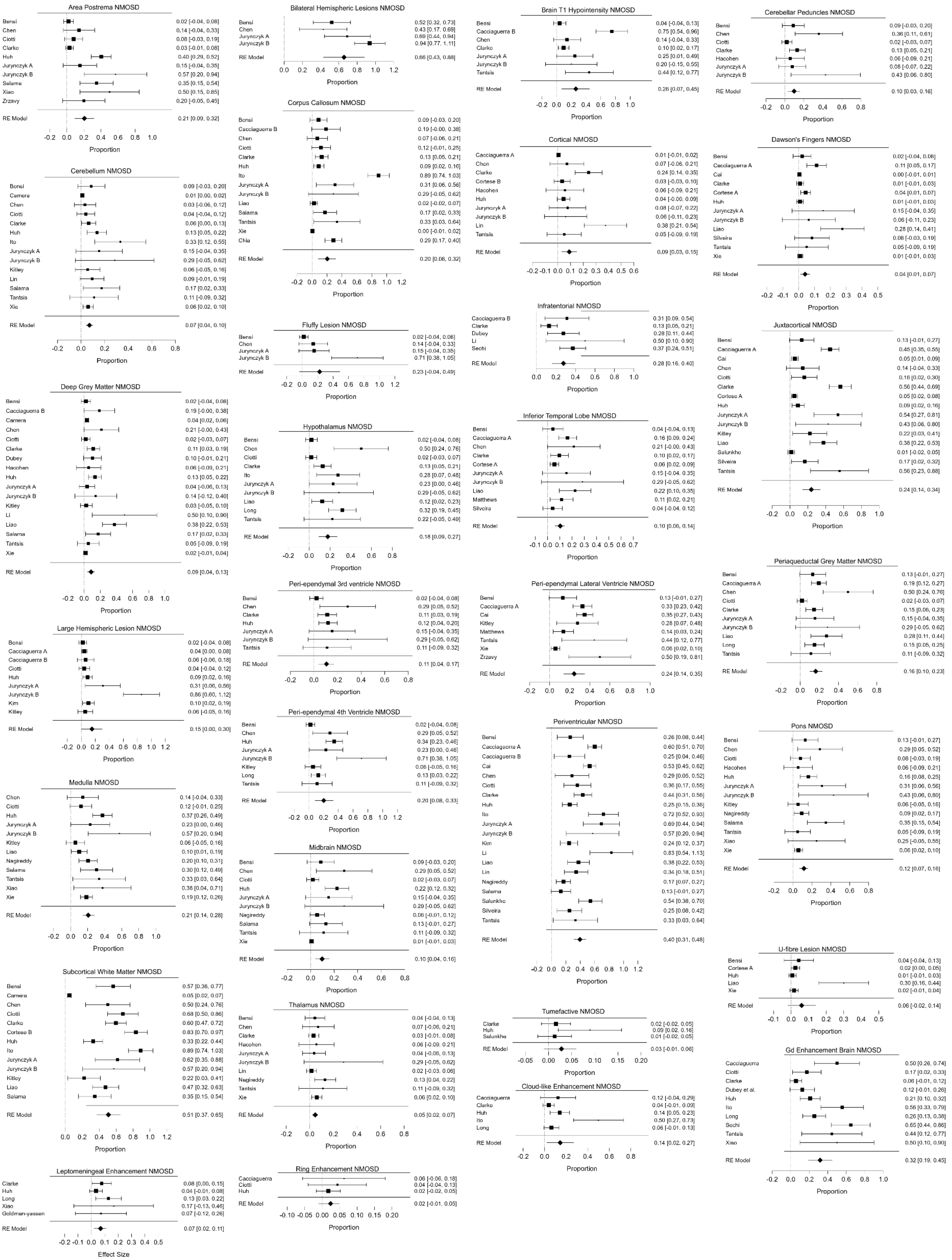

Supplementary figure 9 – Forest plots for individual meta-analyses: NMOSD Brain

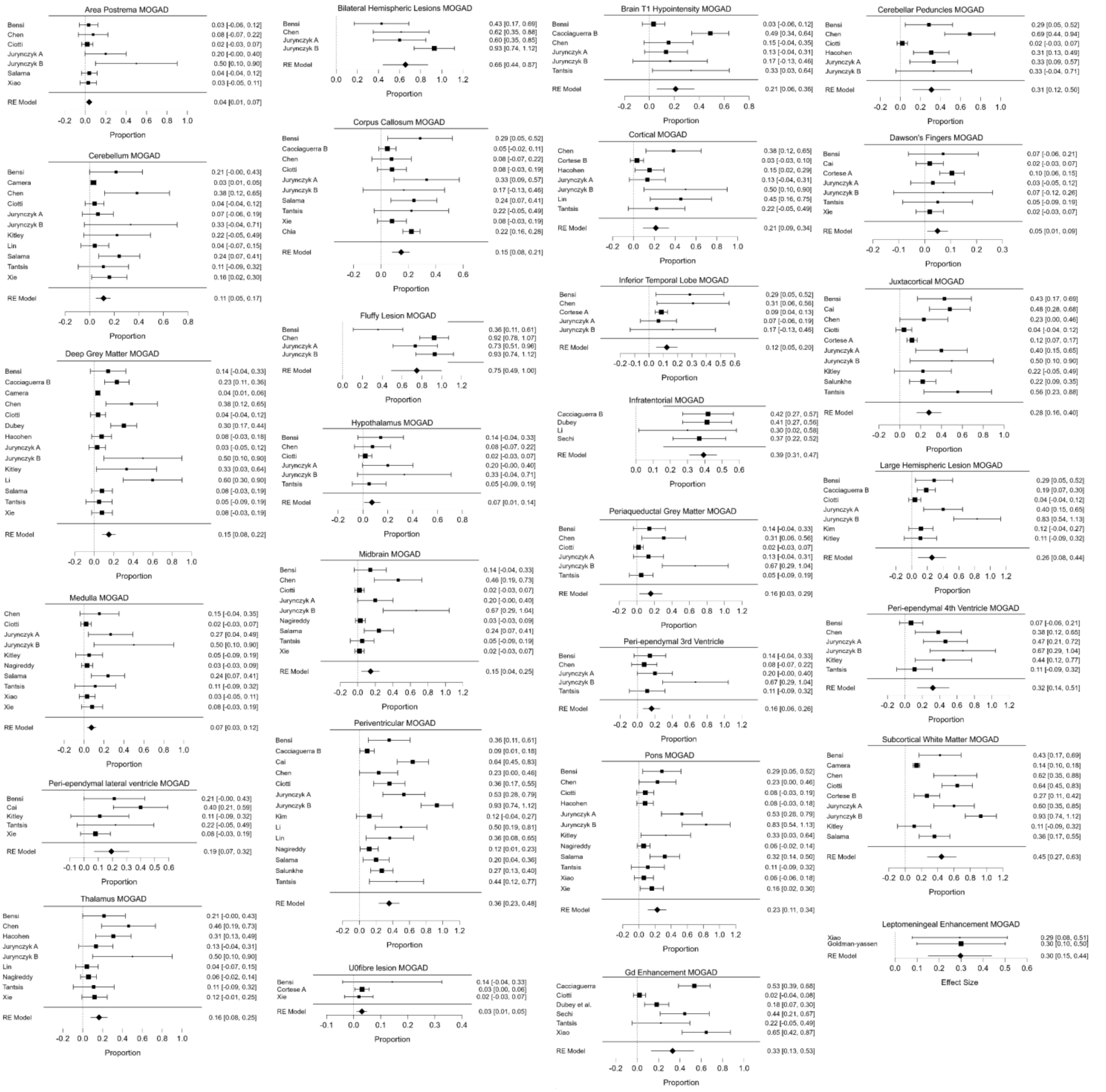

Supplementary figure 10 – Forest plots for individual meta-analyses: MOGAD Brain

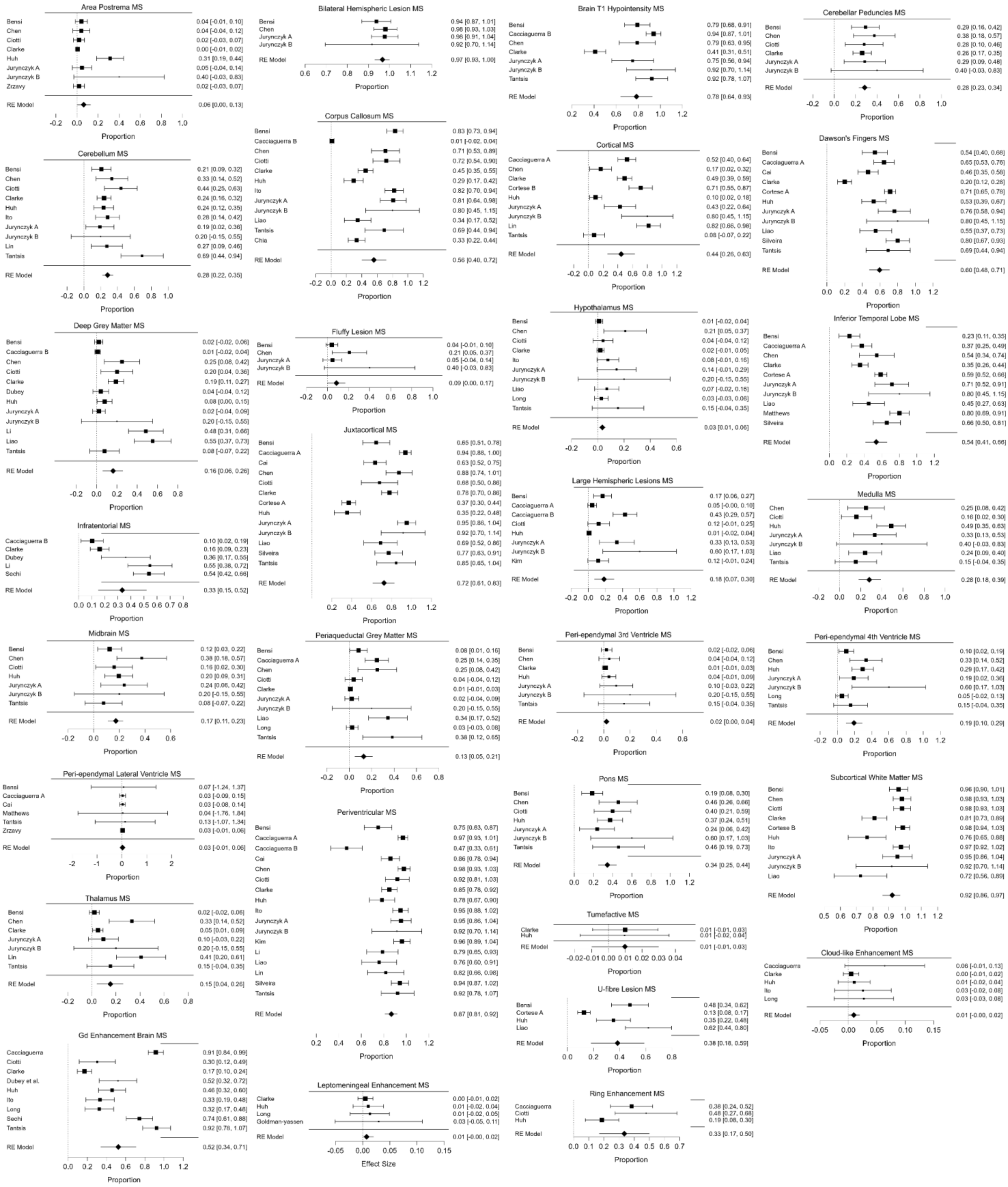

Supplementary figure 11 – Forest plots for individual meta-analyses: MS Brain

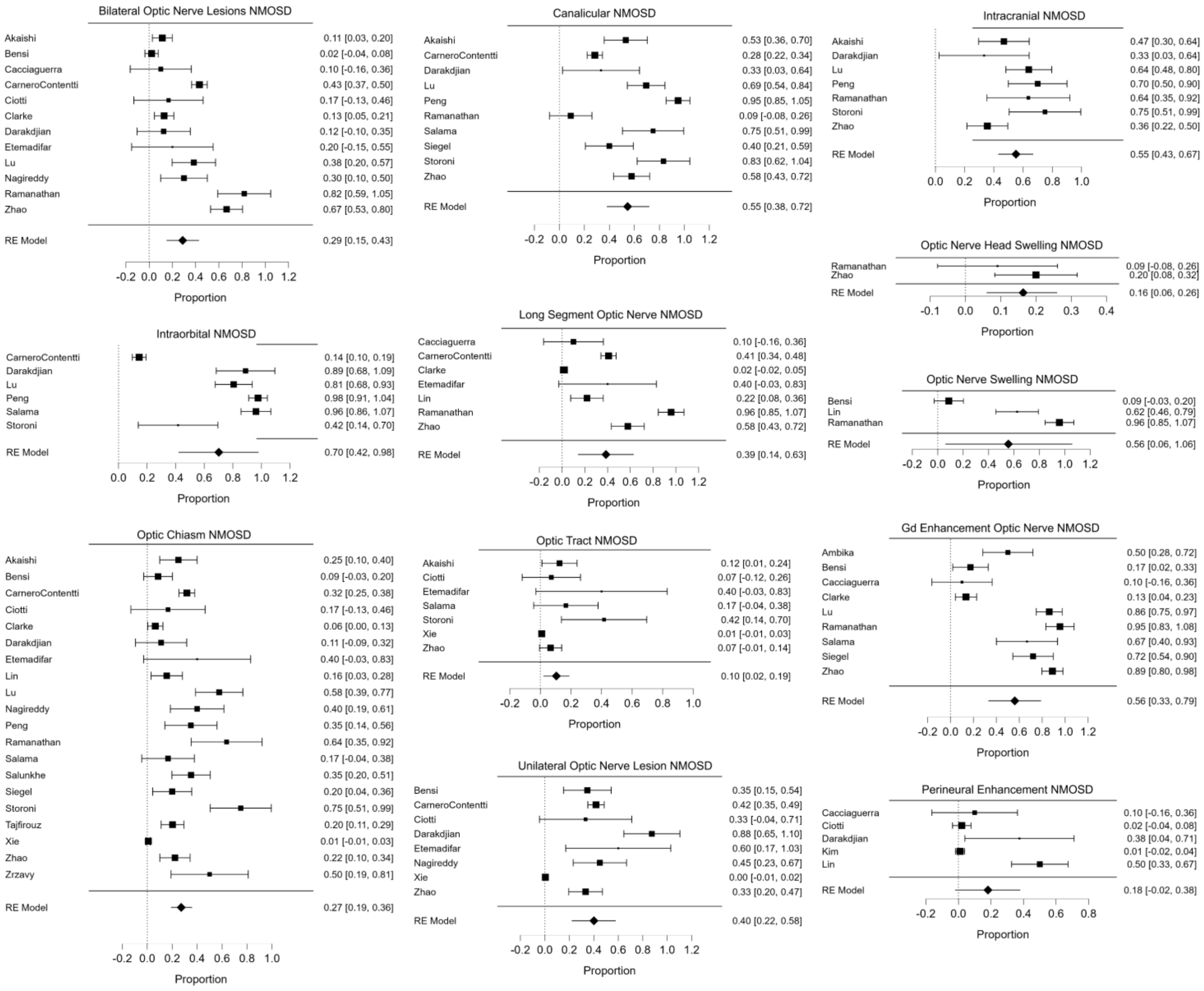

Supplementary figure 12 – Forest plots for individual meta-analyses: NMOSD Orbits

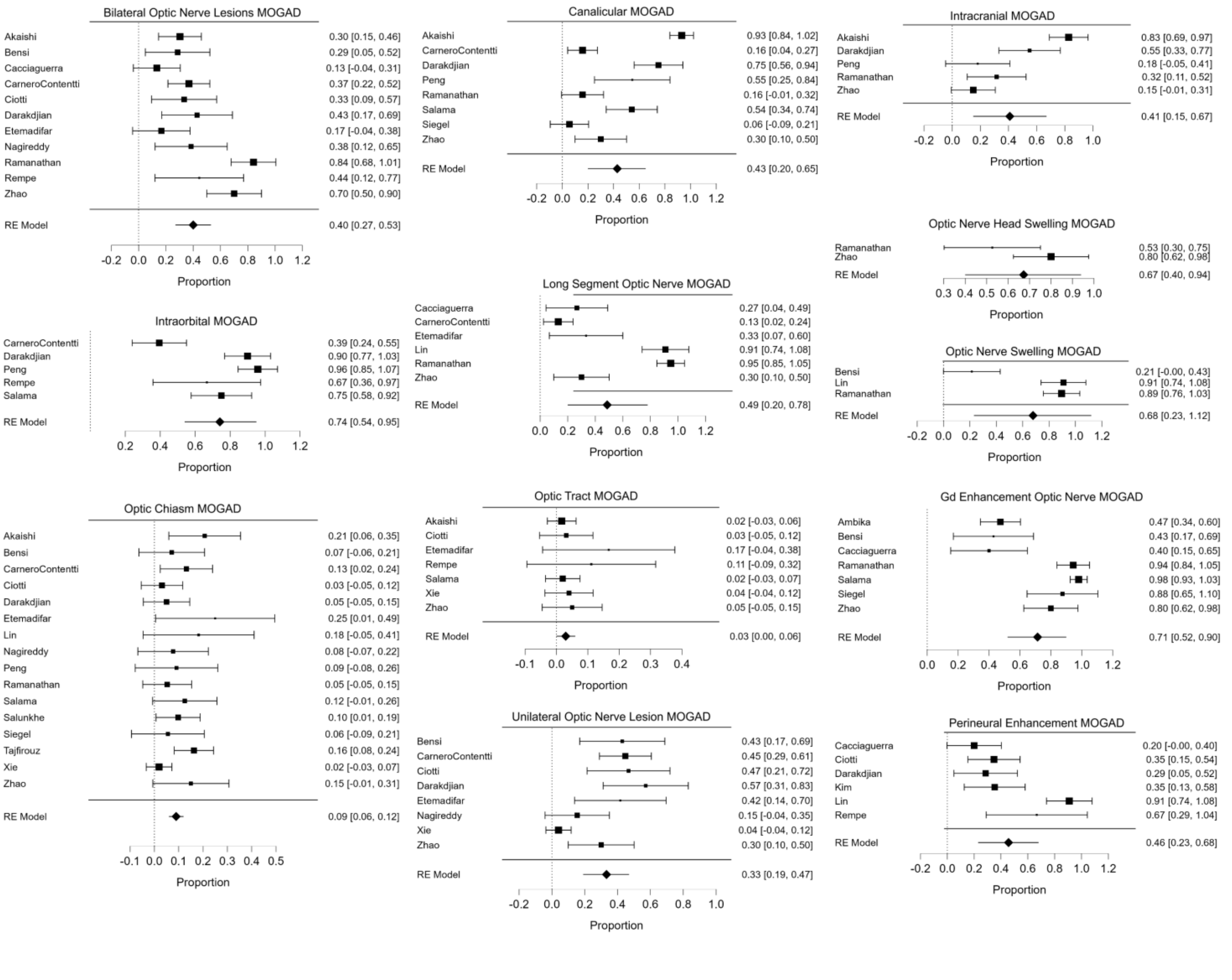

Supplementary figure 13 – Forest plots for individual meta-analyses: MOGAD Orbits

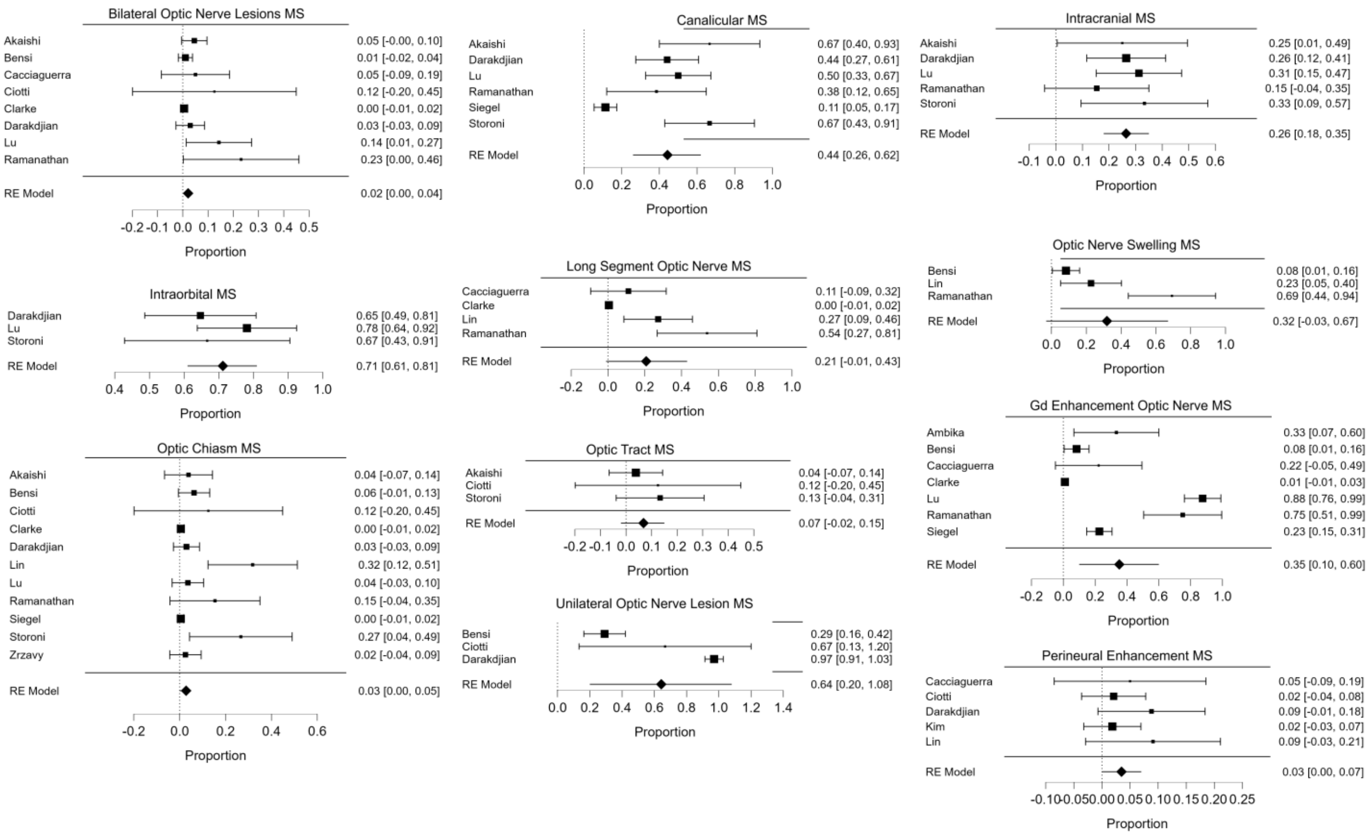

Supplementary figure 14 – Forest plots for individual meta-analyses: MS Orbits

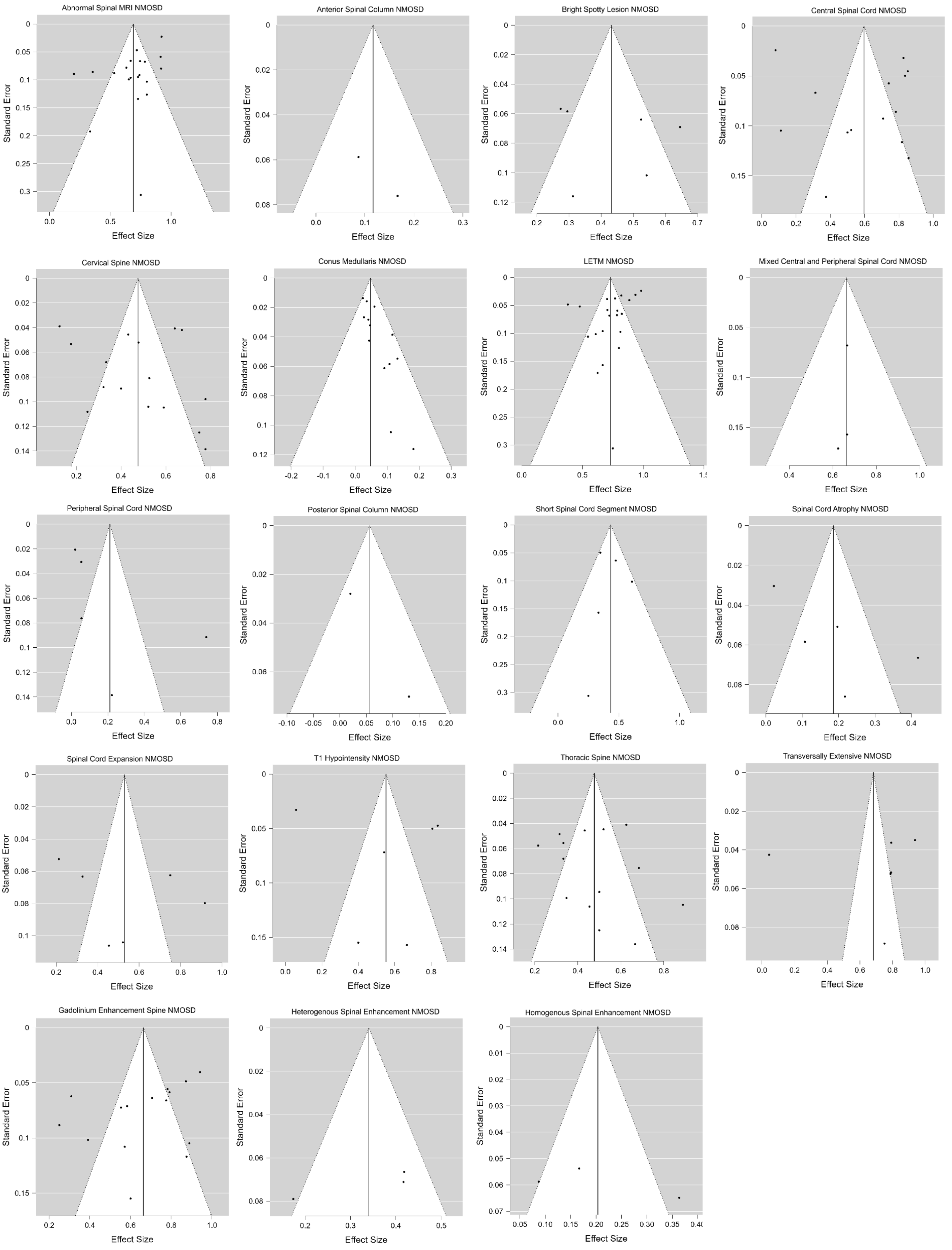

Supplementary figure 15 – Funnel plots for individual meta-analyses: NMOSD Spine

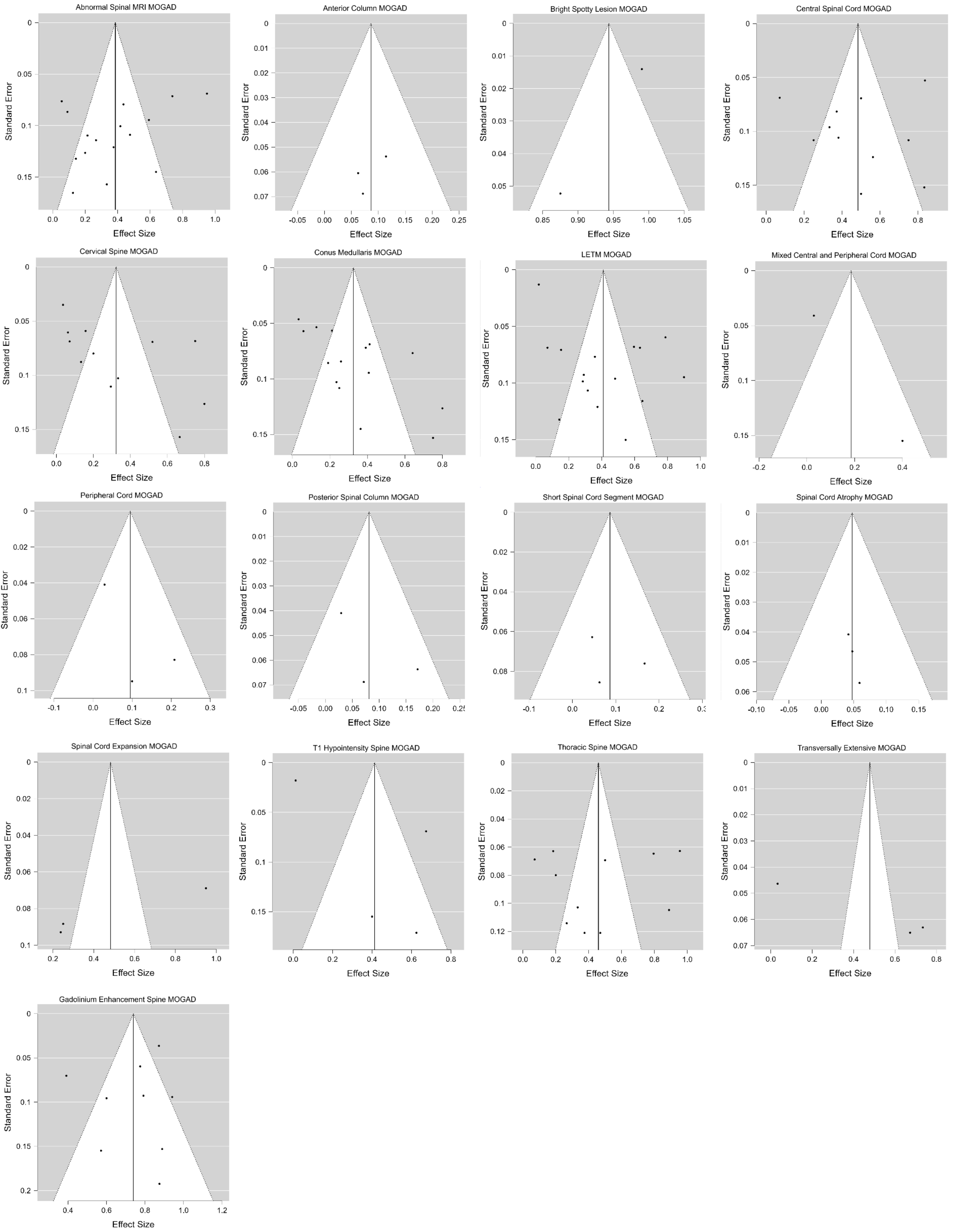

Supplementary figure 16 – Funnel plots for individual meta-analyses: MOGAD Spine

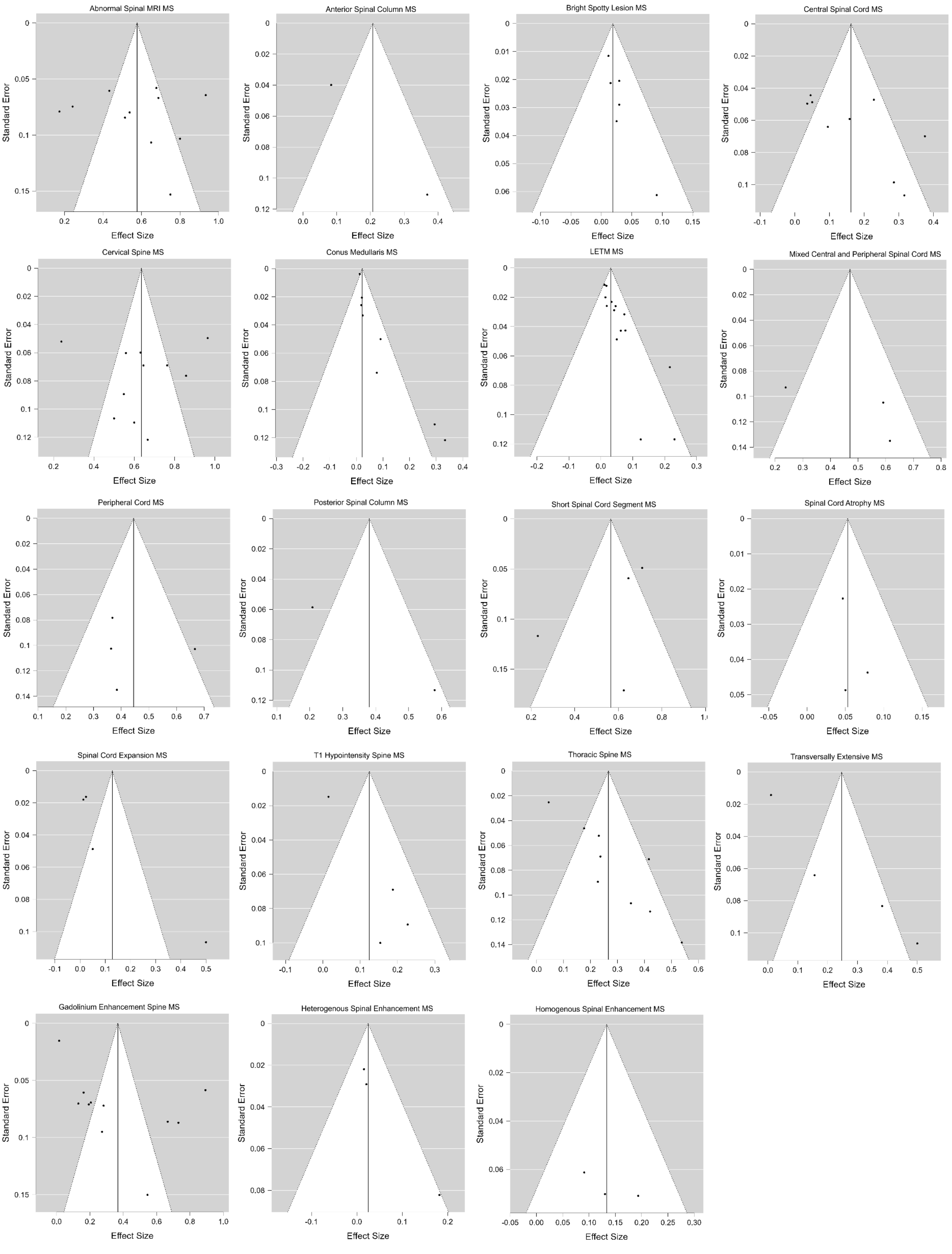

Supplementary figure 17 – Funnel plots for individual meta-analyses: MS Spine

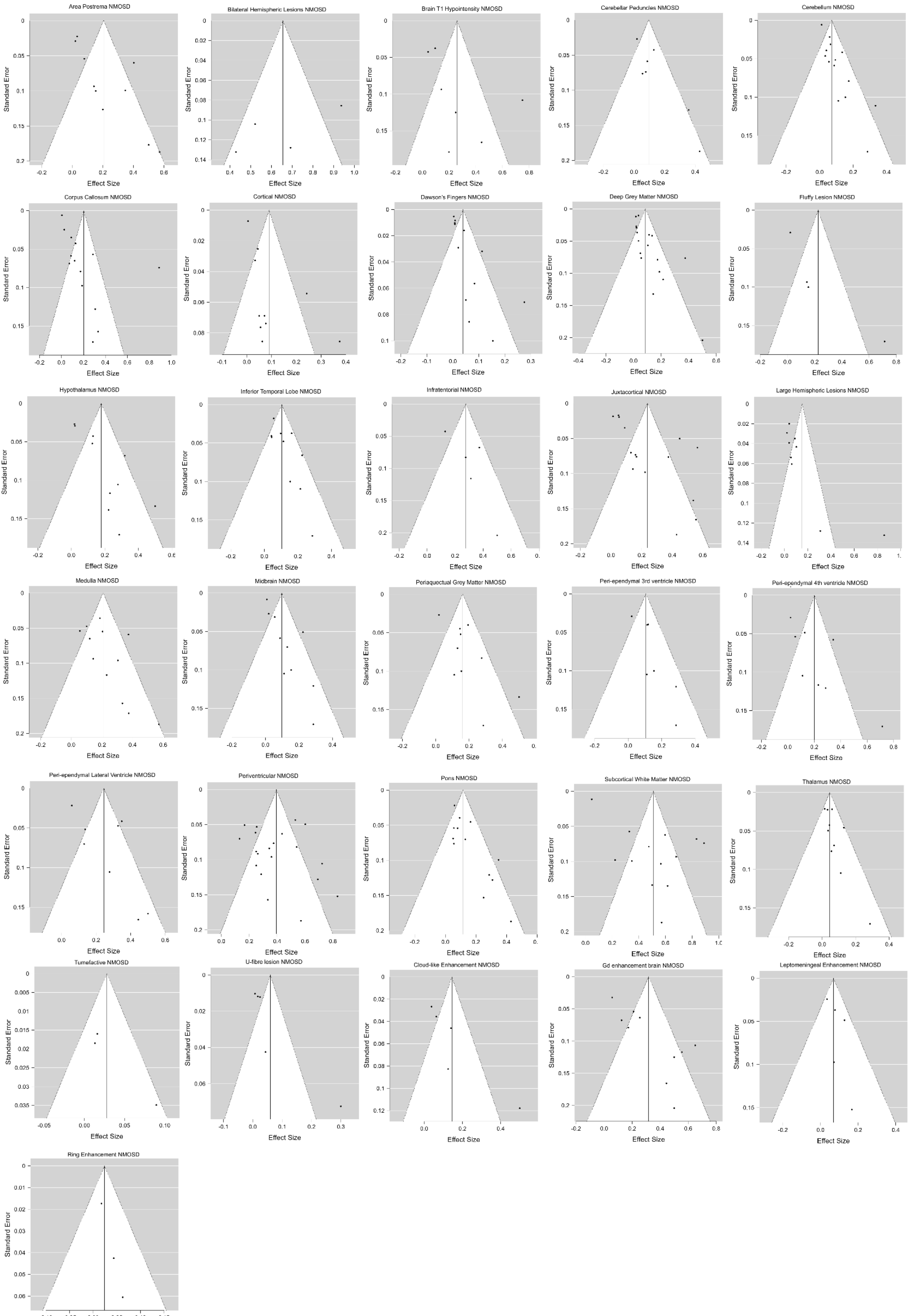

Supplementary figure 18 – Funnel plots for individual meta-analyses: NMOSD Brain

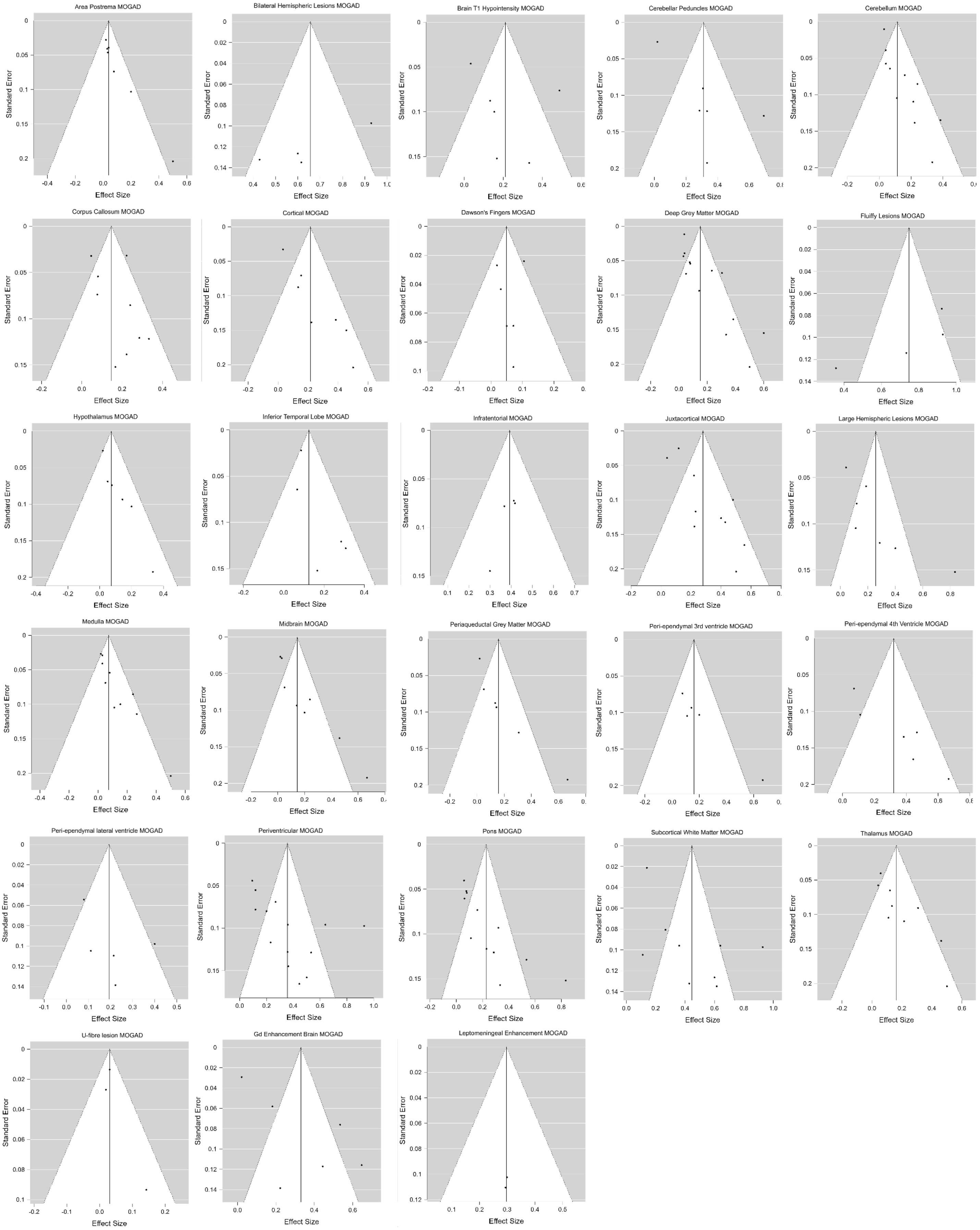

Supplementary figure 19 – Funnel plots for individual meta-analyses: MOGAD Brain

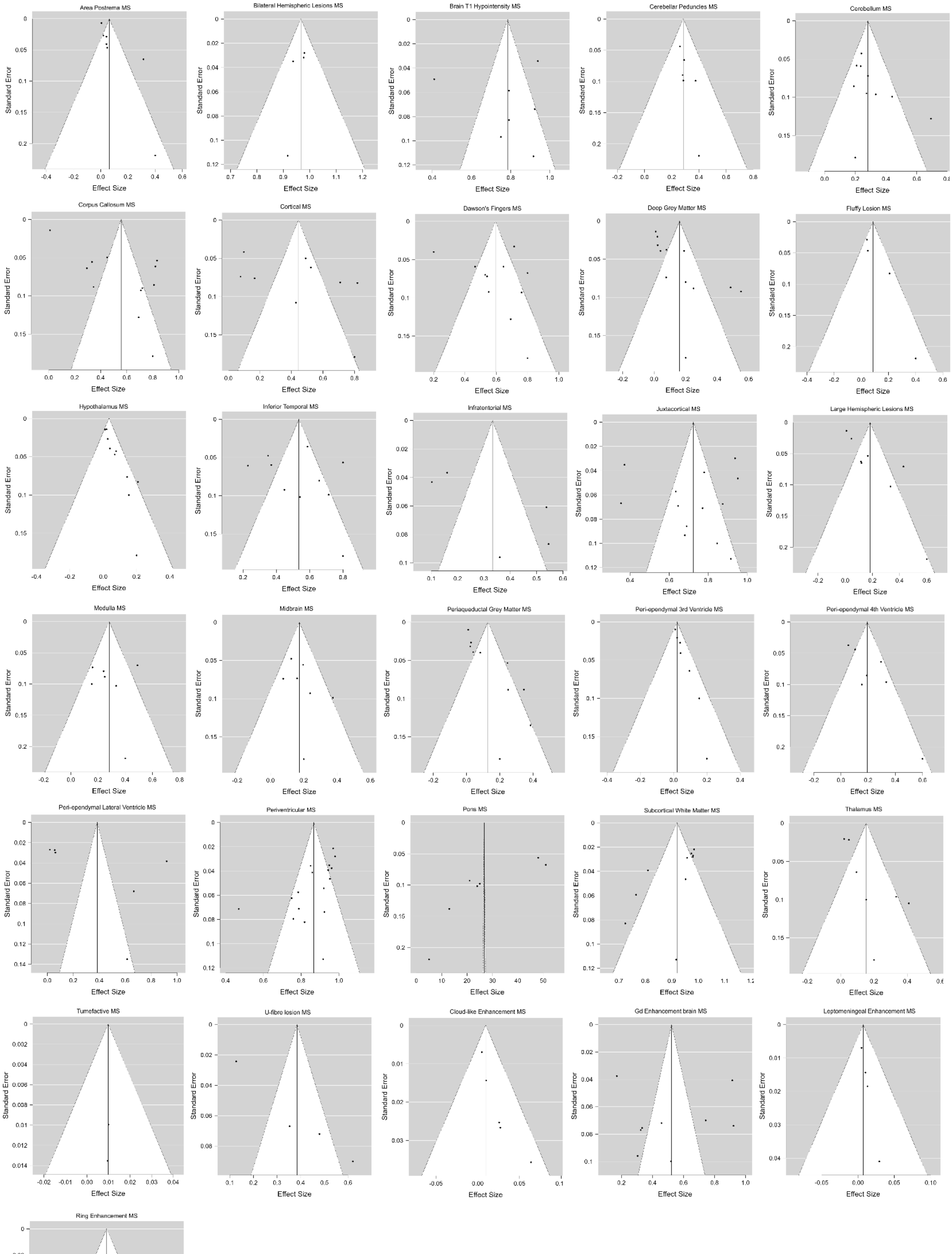

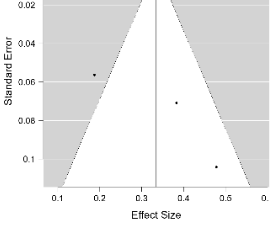

Supplementary figure 20 – Funnel plots for individual meta-analyses: MS Brain

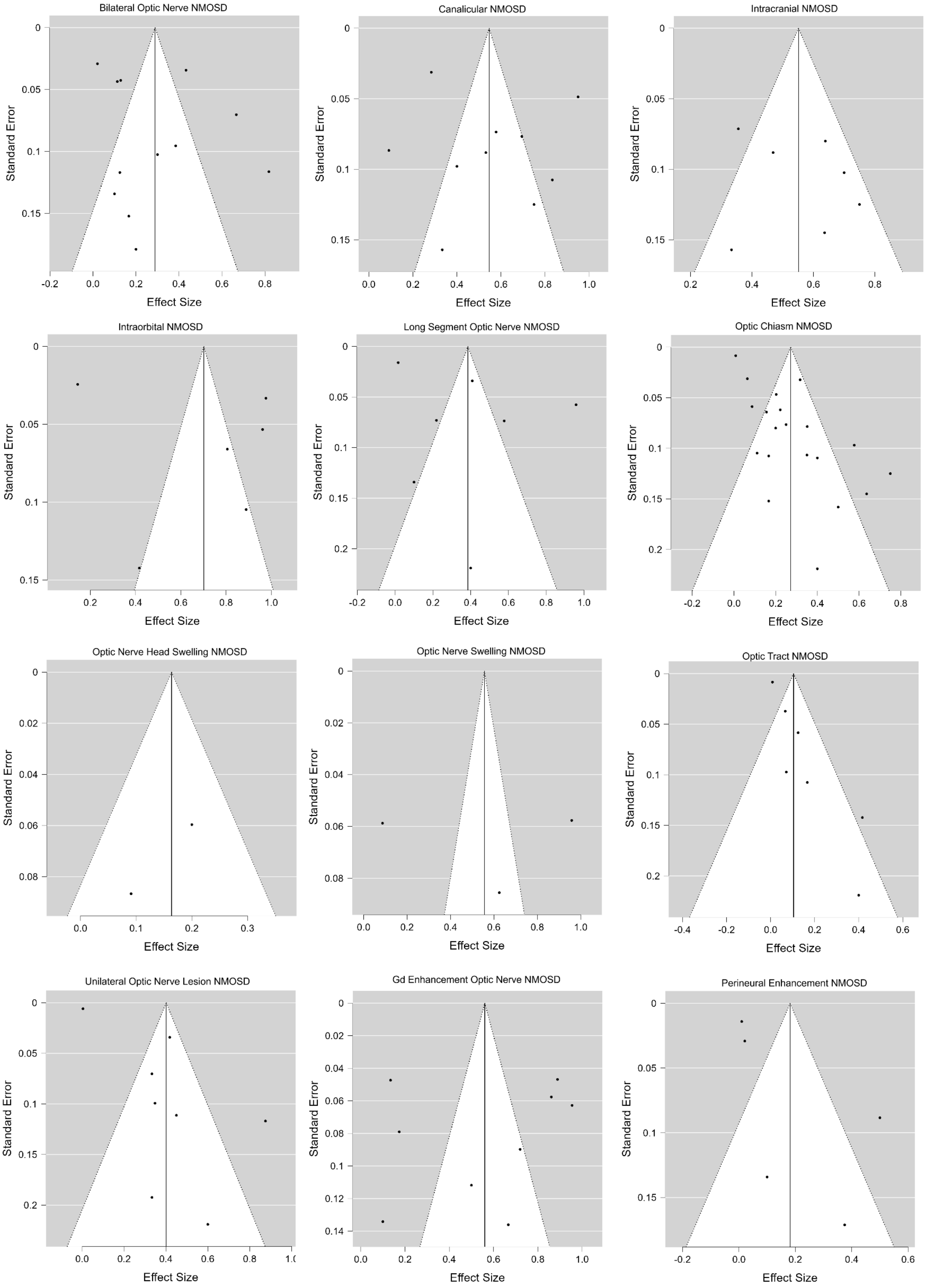

Supplementary figure 21 – Funnel plots for individual meta-analyses: NMOSD Orbits

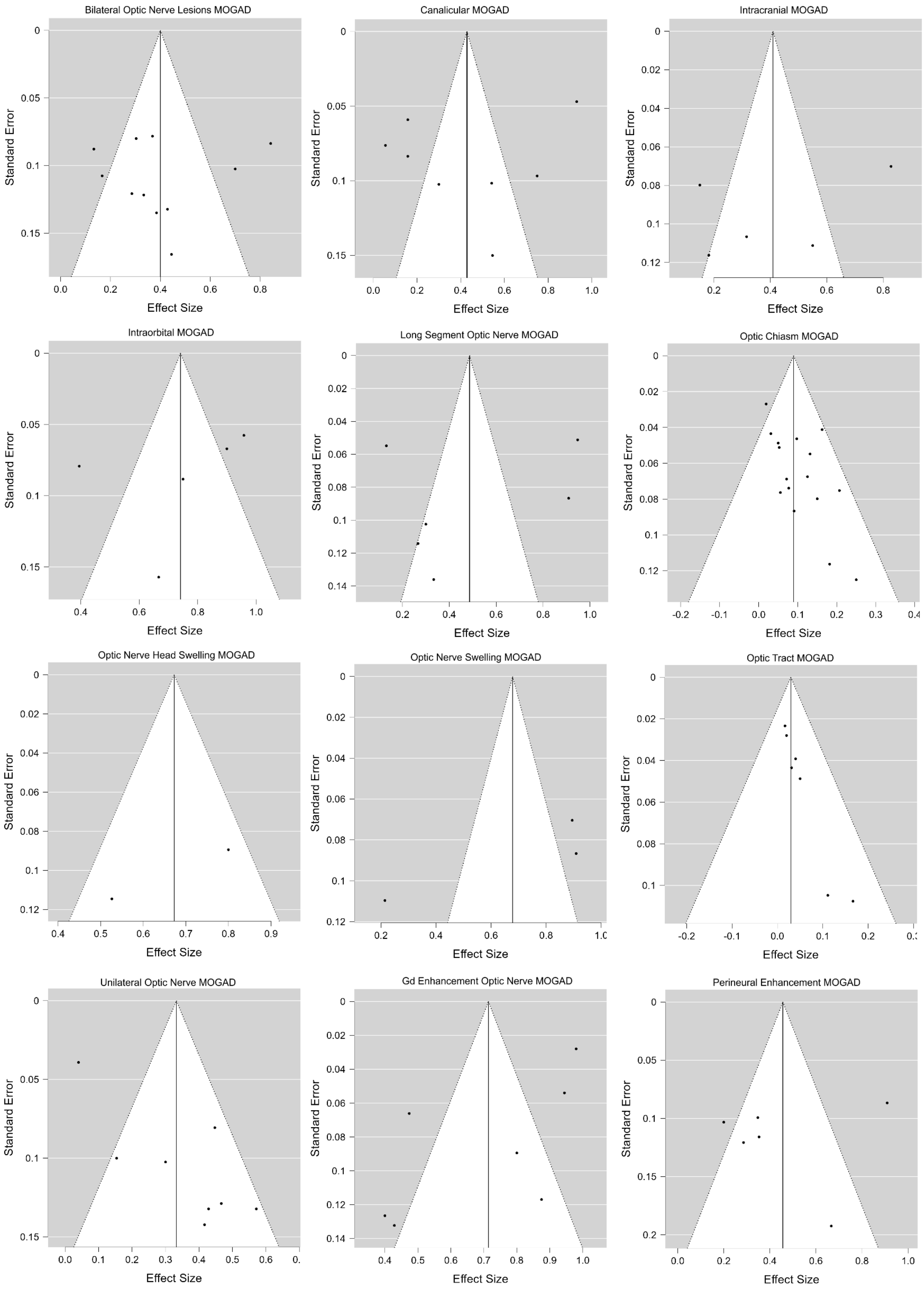

Supplementary figure 22 – Funnel plots for individual meta-analyses: MOGAD Orbits

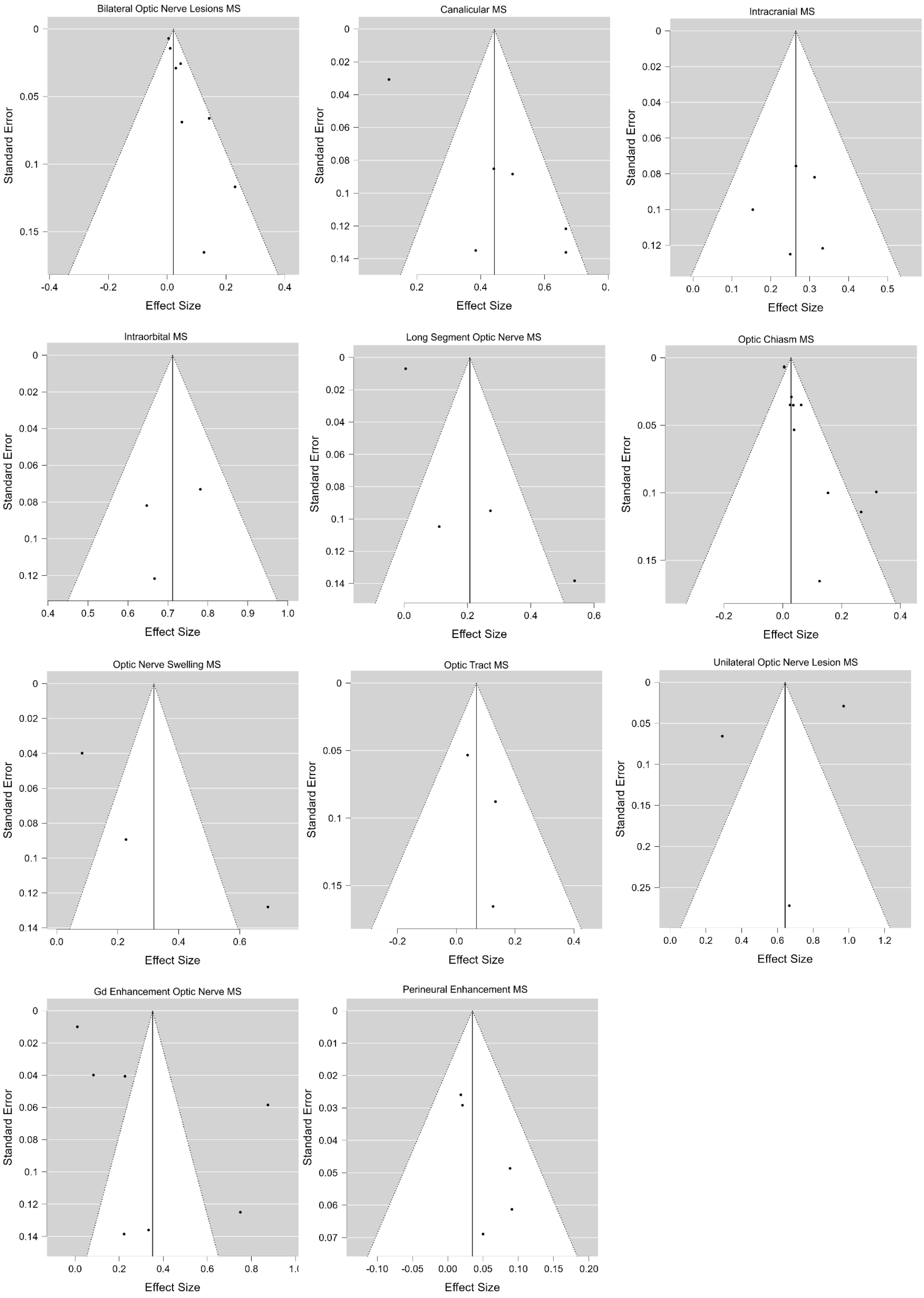

Supplementary figure 23 – Funnel plots for individual meta-analyses: MS Orbits
