# Supplementary material for: Characteristics of MRI lesions in AQP4 antibody-positive NMOSD, MOGAD, and multiple sclerosis: a systematic review and meta-analysis
Source: J Neurol. 2025 Aug 7;272(9):560. doi: 10.1007/s00415-025-13303-w (PMC12331836; doi:10.1007/s00415-025-13303-w)
Supplement: Supplementary file 2 — Supplementary file2 (PDF 890 KB) [file 415_2025_13303_MOESM2_ESM.pdf]

Supplementary Table 1. Search Strategy

((MRI) OR (Magnetic resonance) OR (magnetic resonance imaging) OR (MR))<br><br>*AND*	((MOG) OR (MOGAD) OR (MOG-ad) OR (Myelin Oligodendrocyte Glycoprotein) OR (Myelin Oligodendrocyte glycoprotein associated disease)) AND ((NMO) OR (NMOSD) OR (Neuromyelitis optica) OR (Neuromyelitis optica spectrum disorder))<br><br>*OR*	((MOG) OR (MOGAD) OR (MOG-ad) OR (Myelin Oligodendrocyte Glycoprotein) AND (Myelin Oligodendrocyte glycoprotein associated disease)) AND ((MS) OR (Multiple Sclerosis))<br><br>*OR*	((NMO) OR (NMOSD) OR (Neuromyelitis optica) OR (Neuromyelitis optica spectrum disorder) OR (AQP4) OR (AQP4-NMOSD)) AND ((MS) OR (Multiple Sclerosis))<br><br>*AND*	((Features) OR (Patterns) OR (findings) OR (characteristics) OR (Lesions))<br><br>*AND*	((Cohort study) OR (Case control))

MRI = magnetic resonance imaging; MOG = myelin oligodendrocyte glycoprotein; MOGAD = myelin oligodendrocyte glycoprotein antibody associated disease; NMO = neuromyelitis optica; NMOSD = neuromyelitis optica spectrum disorder; AQP4 = aquaporin-4; MS = multiple sclerosis

Supplementary Table 2. Study Characteristics

Author[ref]	Orbits/ Spine/ Brain	Phase	Study Design	Location	Cohort	Sample size	Female n (%)	Age at Time of Study (yrs) Mean (SD)	Age of Onset (yrs) Mean (SD)	Duration (months) of Disease Mean (SD)	EDSS*	Quality Score
Akaishi et al. 2016[1]	Orbits	Acute	Retrospective	Japan	NMOSD	53	52 (98)					7
					MOG	33	17 (52)					
					MS	66	53 (80)					
Ambika et al. 2021[2]	Orbits	Unknown	Retrospective	India	NMOSD	20		30.06 (14.72)				6
	Spine				MOGAD	57		29.6 (15.4)				
					MS	12		31.08 (9.75)				
Bensi et al. 2018[3]	Orbits	Unknown	Retrospective	Argentina	NMOSD	23	16 (70)		42.4 (10.11)	69.5 (45.1)		7
	Spine				MOGAD	14	9 (64)		30.2 (22.6)	17.5 (10.57)		
	Brain				MS	48	34 (71)		33.65 (7.17)	82.5 (47.02)		
Cacciaguerra et al. 2019[4]	Spine	Chronic	Retrospective	Italy,	NMOSD	116	91 (78)	44.5 (13.5)		71.4 (66.7)	4 [2.9-6.5]	8
	Brain			Serbia, UK	MS	65	46 (71)	40.2 (11.3)		83.2 (46.4)	1.5 [1.5-2.5]	
Cacciaguerra et al. 2023[5]	Orbits	Unknown	Retrospective	USA	NMOSD	16	16 (100)	37.5 (7.36)			4 [3.5-7.5]	8
	Spine				MOGAD	43	23 (53)	21.75 (6.62)			3.5 [2-4]	
	Brain				MS	49	31 (63)	34.75 (4.69)			3 [2.5-4.5]	
Cai et al. 2019[6]	Brain	Acute	Retrospective	China	NMOSD	31						7
					MS	33						
Cai et al. 2021[7]	Spine	Acute	Retrospective	China	NMOSD	143	130 (91)		43.1 (15.1)			7
	Brain	(3 months)			MOGAD	25	9 (36)		36.3 (18.4)			
					MS	73	45 (62)		36 (13.2)			

Author[ref]	Orbits/ Spine/ Brain	Phase	Study Design	Location	Cohort	Sample size	Female n (%)	Age at Time of Study (yrs) Mean (SD)	Age of Onset (yrs) Mean (SD)	Duration (months) of Disease Mean (SD)	EDSS*	Quality Score
Camera et al. 2021[8]	Orbits	Unknown	Retrospective	UK	NMOSD	222	189 (85)	51.75 (14.99)	43.25 (14.63)	111.5 (44.96)		8
					MOGAD	182	113 (62)	40.75 (13.5)	33.25 (13.5)	92 (44.75)		
	Spine											
	Brain											
Carnero Contentti et al. 2021[9]	Orbits	Acute	Retrospective		NMOSD	208	175 (84)		38.5 (14.6)	36.6 (37.5)	4 (0-9)	7
					MOGAD	38	30 (79)		32.2 (14.2)	19.3 (23.1)	2 (0-6)	
Chen et al. 2021[10]	Brain	Acute	Retrospective	China	NMOSD	14	13 (93)		42.25 (12.33)	32 (35.22)		7
					MOGAD	13	4 (31)		41.25 (9.89)	1.25 (0.90)		
					MS	24	14 (58)		32.75 (10.01)	42.5 (36.97)		
Chia et al. 2023[11]	Brain	Relapse	Retrospective		NMOSD	18	16 (89)	49.75 (20.60)				6
					MOGAD	38	20 (53)	23.25 (12.62)				
					MS	24	19 (80)	33.25 (8.73)				
Chien et al. 2019[12]	Spine	Unknown	Retrospective	Germany	NMOSD	38	35 (92)	48.1 (14.5)		118.3 (72.8)	4 (0-7.6)	8
					MOGAD	15	11 (73)	41.7 (12.7)		69.6 (47)	2.5 (1-6)	
					HC	24	18 (75)	41.3 (13.5)				
Ciotti et al. 2022[13]	Orbits	Acute	Case-control	USA	NMOSD	26		43.171 (13.65)		105.6 (69.6)	3 [2-5]	8
	Spine	(6 months)			MOGAD	26		45.375 (19.22)		40.3 (33.9)	2 [1-3]	
	Brain				MS	26		44.2198 (16.24)		113.06 (153.42)	2 [2-6]	
Clarke et al. 2021[14]	Orbits	All	Cross-sectional	Australia, NZ	NMOSD	67	60 (90)	50.5 (14.02)	45 (15.30)	152.4 (109.6)	4 (0-9)	7
	Spine				MS	100	85 (85)	45.25 (11.406)	32.25 (10.61)	204.3 (103)	2 (0-9)	
	Brain											

Author(ref)	Orbits/ Spine/ Brain	Phase	Study Design	Location	Cohort	Sample size	Female n (%)	Age at Time of Study (yrs) Mean (SD)	Age of Onset (yrs) Mean (SD)	Duration (months) of Disease Mean (SD)	EDSS*	Quality Score
Combes et al. 2017[15]	Spine	All			NMOSD	15	12 (80)	46.5 (16.13)		79.5 (50.12)	4 (2-7.5)	6
					MS	15	11 (73)	43 (13.25)		105.5 (66.24)	2 (0-5)	
					HC	17	13 (76)	50.75 (15.89)				
Cortese et al. 2023[16]	Spine Brain	Chronic	Prospective	UK	NMOSD	162	132 (81)	50.65 (14.14)	42.87 (13.69)	102 (98.4)	3.5 (0-8)	8
					MOGAD	162	99 (61)	40.59 (14.09)	34.43 (14.33)	69.6 (90)	2 (0-7.5)	
					MS	189	132 (70)	39.66 (10.44)	32.27 (8.57)	93.6 (81.6)	2 (0-8)	
					HC	152	91 (60)	37.38 (11.43)				
Cortese et al. 2023[17]	Spine Brain	Chronic	Prospective	UK	NMOSD	30	24 (80)	49.4 (12.2)	40.6 (12.9)	106.8 (97.2)	4.5 (1.5-6.5)	8
					MOGAD	30	20 (67)	36.9 (16.7)	31.7 (17.9)	63.6 (66)	2 (1-6.5)	
					MS	31	19 (61)	45.7 (11.8)	34.9 (9.9)	130.8 (81.6)	2 (1-7.5)	
					HC	34	24 (71)	34.7 (11.8)				
Darakdjian et al. 2022[18]	Orbits	Acute	Retrospective	Argentina	NMOSD	8	7 (88)					7
					MOGAD	14	7 (50)					
					MS	34	25 (74)					
Dubey et al. 2019[19]	Spine Brain	Relapse	Retrospective	USA	NMOSD	46	39 (85)	47.25 (13.54)				8
					MOGAD	54	24 (44)	31.5 (15.4)				
					MS	26	20 (77)	36.75 (10.43)				
Dumrikarnlert et al. 2017[20]	Spine	Unknown	Retrospective	Thailand	NMOSD	55	53 (96)		38 (15.2)		4 (0-9)	7
					MS	38	33 (87)		38 (15.6)		2 (0-7.5)	
Etemadifar et al. 2019[21]	Orbits	Acute	Cross-sectional	Canada	NMOSD	5	4 (80)	30.8 (7)				8
					MOGAD	12	7 (58)	31.3 (5.9)				
					MS	78	60 (77)	28.4 (6.9)				

Author[ref]	Orbits/ Spine/ Brain	Phase	Study Design	Location	Cohort	Sample size	Female n (%)	Age at Time of Study (yrs) Mean (SD)	Age of Onset (yrs) Mean (SD)	Duration (months) of Disease Mean (SD)	EDSS*	
Etemadifar et al. 2021[22]	Spine	All	*Subset 1 – all participants*		NMOSD	150	117 (78)	39.93 (11.59)				8
					MOGAD	27	19 (70)	28.7 (5.23)				
					MS	863	652 (76)	32.47 (9.12)				
			Subset 2 – conus involvement only		NMOSD	9	7 (78)	38.78 (13.97)			1.5 (1-2.5)	
					MOGAD	7	5 (71)	29 (5.66)			2 (1.5-2.5)	
					MS	11	8 (73)	38.64 (14.17)			1.5 (1-4)	
Fadda et al. 2021[23]	Spine	Acute	Retrospective	Canada	MOGAD	40	23 (58)		7.37 (5.91)		1.25 [0.5-2]	8
					MS	21	14 (67)		13.89 (1.32)		1 [0-2]	
Goldman-Yassen 2024[24]	Brain	All	Retrospective	USA	NMOSD	6	6 (100)		12.06 (4.58)			8
					MOGAD	20	14 (70)		8.36 (5.58)			
					MS	16	12 (75)		14.31 (2.84)			
Hacohen et al. 2017[25]	Orbits Spine Brain	All	Retrospective	UK Ireland	NMOSD	8	7 (88)	9.56 (4.91)			2 [1.25-3.38]	8
					MOGAD	26	16 (620	6 (3.14)			1 [0-2]	
Huh et al. 2014[26]	Spine Brain	All	Retrospective	Korea	NMOSD	67	57 (85)	43.87 (12.31)	38.34 (12.98)			7
					MS	51	34 (66)	35.2 (9.11)	31.41 (9.14)			
Hyun et al. 2015[27]	Spine	Acute	Retrospective	Korea	NMOSD	59	51 (86)	38.3 (11.3)	35.9 (11.4)			8
					MS	31	16 (52)	36.1 (9.3)	34.2 (9.8)			
Hyun et al. 2022[28]	Spine	Acute	Retrospective	Korea	NMOSD	61	53 (87)	38.82 (12.15)			3.5 [2.5-8]	7
					MOGAD	49	29 (59)	36.42 (10.69)			3.5 [2-7]	
Ito et al. 2009[29]	Brain	All	Prospective	Japan	NMOSD	18	18 (100)		37.6			7
					MS	39	32 (82)		29.7			

Author[ref]	Orbits/ Spine/ Brain	Phase	Study Design	Location	Cohort	Sample size	Female n (%)	Age at Time of Study (yrs) Mean (SD)	Age of Onset (yrs) Mean (SD)	Duration (months) of Disease Mean (SD)	EDSS*	Quality Score
Jurynczyk et al. 2017[30]	Brain	Unknown	Prospective	UK	NMOSD	13	11 (85)	47.6		69.75 (77.64)		8
			Subset 1 – Adults		MOGAD	15	8 (53)	34.9		68.5 (77.77)		
					MS	21	15 (71)	32.2		51 (36.53)		
					NMOSD	7	6 (86)	9.8		17 (23.67)		
			Subset 2 - Paediatrics		MOGAD	6	3 (50)	9.1		0.75 (0.40)		
					MS	5	2 (40)	14.4		6.25 (9.03)		
Kim et al. 2015[31]	Orbits	Unknown	Prospective	Korea	NMOSD	49	42 (86)		43.41 (13.96)			8
	Spine				MOGAD	17	10 (59)		42.69 (19.3)			
	Brain				MS	26	14 (54)		32.8 (10)			
Kitley et al. 2014[32]	Spine	All	Prospective	UK	NMOSD	9	4 (44)		44.86 (14.8)		5.5 (1-8.5)	8
	Brain				MOGAD	20	18 (90)		32.29 (17.1)		6 (4-8.5)	
Li et al. 2022[33]	Spine	Acute	Retrospective	China	NMOSD	6			26.5 (9.2)	40.6 (34.3)	6 [2.8-8.3]	7
	Brain				MOGAD	10			40.8 (14.8)	19.6 (10.3)	4.5 [2.5-6.7]	
					MS	33			37.3 (14.5)	24 (18.6)	4 [3-5.9]	
Liao et al. 2014[34]	Brain	Relapse	Retrospective	Taiwan	NMOSD	25	22 (88)		37.8 (13.6)			7
					MS	29	22 (76)		33.7 (9.2)			
Lin et al. 2023[35]	Orbits	All	Retrospective	Taiwan	NMOSD	43	39 (91)		43.4 (21.9)			7
	Spine		Cohort		MOGAD	11	8 (72)		41.8 (15.3)			
	Brain				MS	31	25 (81)		32.5 (14.3)			
Long et al. 2014[36]	Spine	Acute	Retrospective	China	NMOSD	47	45 (96)		34.3 (14)		2.5 (0-6.5)	6
	Brain				MS	37	20 (54)		31.5 (12.2)		4.5 (1-10)	
Lu et al. 2018[37]	Orbits	Acute	Retrospective	China	NMOSD	26	21 (81)	36.1 (12.8)		12.8 (7)		7

					MS	28	18 (64)	39.8 (14.7)		11.4 (5.9)		

Author[ref]	Orbits/ Spine/ Brain	Phase	Study Design	Location	Cohort	Sample size	Female n (%)	Age at Time of Study (yrs) Mean (SD)	Age of Onset (yrs) Mean (SD)	Duration (months) of Disease Mean (SD)	EDSS*	Quality Score
Mariano et al. 2019[38]	Spine	Acute	Retrospective cross-sectional		NMOSD	69	57 (83)		48 (15)	113.75 (59.93)	7 (3-9.5)	7
					MOGAD	46	24 (52)		34 (11)	114.88 (89.22)	5.5 (1-9)	
Marrodan et al. 2020[39]	Spine	Acute	Retrospective	Argentina	NMOSD	16	12 (75)		46.5 (10.3)			7
					MS	68	42 (62)		35.8 (11.9)			
Masuda et al. 2022[40]		Acute + F/U	Retrospective Cohort		NMOSD	36	32 (89)	55 (10.15)		167.3 (120.8)	2 (0-7.5)	8
					MS	60	46 (77)	41.25 (10.81)		155.2 (88.9)	4.5 (1-9)	
Matthews et al. 2013[41]	Brain	All	Cross-sectional		NMOSD	44	39 (89)	47.5 (4.40)		101.3 (76.8)	4.9 +/- 2.1	7
					MS	50	34 (68)	43.75 (8.67)		147.6 (81.6)	1.83 +/- 1.3	
Matthews et al. 2015[42]	Spine	Unknown	Prospective	UK	NMOSD	18	15 (83)	47 (15.39)		78.25 (47.80)		8
					MS	15	11 (73)	40 (11.52)		102 (62.21)		
					HC	17	13 (76)	48 (15.61)				
Nagireddy et al. 2021[43]	Orbits Spine Brain	Unknown	Prospective	India	NMOSD	28	18 (64)	28.5 (8.20)	27.25 (8.20)	27.6 (21.6)		6
					MOGAD	22	11 (50)	19.25 (8.64)	18 (8.38)	21.6 (7.2)		
Papadopoulou et al. 2019[44]	Spine	Unknown	Cross-sectional		NMOSD	39	36 (92)	50.1 (14.1)		105.6 (96)	4 (0-7)	7
					HC	37	32 (86)	47.8 (12.5)				
Pekcevik et al. 2016[45]	Spine	Acute	Retrospective	USA	NMOSD	48	44 (92)	44.4 (16.4)				7
					MS	22	17 (77)	41.6 (13)				
Peng et al. 2018[46]	Orbits Spine	Acute	Retrospective	China	NMOSD	20	19 (95)		33.65 (11.29)			7
					MOG	11	7 (64)		31.55 (13.03)			

Author[ref]	Orbits/ Spine/ Brain	Phase	Study Design	Location	Cohort	Sample size	Female n (%)	Age at Time of Study (yrs) Mean (SD)	Age of Onset (yrs) Mean (SD)	Duration (months) of Disease Mean (SD)	EDSS*	Quality Score
Ramanathan et al. 2016[47]	Orbits	Acute	Retrospective	Australia	NMOSD	11	9 (81)		23.5 (15.76)			7
				USA	MOGAD	19	15 (79)		21.5 (14.10)			
					MS	13	12 (92)		28.75 (9.89)			
Rempe et al. 2021[48]	Orbits	All	Retrospective	USA	NMOSD	43			40.5 (17.36)			7
	Spine				MOGAD	11			15.5 (10.09)			
Salama et al. 2020[49]	Orbits	Acute + F/U	Retrospective	USA	NMOSD	25	18 (72)	40.5 (12.72)	40.5 (12.72)		5.5 (0-8)	8
	Spine				MOGAD	26	18 (69)	40.5 (16.40)	36.13 (15.77)		2 (0-4)	
	Brain											
Salunkhe et al. 2023[50]	Orbits	All	Prospective Cohort	India	NMOSD	37	30 (81)	29.5 (10.33)		60.25 (40.15)	7.75 (1-8.5)	7
	Spine				MOGAD	41	17 (41)	30.5 (12.9)		53.75 (35.25)	7.25 (3-8)	
	Brain											
Sato et al. 2014[51]	Spine	Unknown	Prospective	Brazil	NMOSD	139	122 (88)	48.75 (12.82)	39 (14.16)		5.8 (1-8.5)	7
				Japan	MOGAD	16	6 (38)	40 (18.12)	37 (18.97)		1.5 (0-8)	
Sechi et al. 2021[52]	Spine	Acute + F/U	Retrospective	USA	NMOSD	51	41 (80)		48.5 (15.10)			7
	Brain				MOGAD	38	17 (45)		31.5 (16.82)			
					MS	67	51 (76)		37.75 (9.56)			
Siegel et al. 2022[53]	Orbits	Acute	Retrospective	USA	NMOSD	25	21 (84)	52 (14.9)				7
					MOGAD	8	5 (63)	51 (8.6)				
					MS	106	83 (78)	45 (13.9)				
Silveira et al. 2020[54]	Orbits	Acute	Retrospective	Argentina	NMOSD	24	22 (92)		37.7 (3)			7
	Spine			Brazil	MS	35	27 (77)		35.4 (5)			
	Brain											

Author[ref]	Orbits/ Spine/ Brain	Phase	Study Design	Location	Cohort	Sample size	Female n (%)	Age at Time of Study (yrs) Mean (SD)	Age of Onset (yrs) Mean (SD)	Duration (months) of Disease Mean (SD)	EDSS*	Quality Score
Storoni et al. 2013[55]	Orbits	Acute	Retrospective	UK	NMOSD	12	10 (83)	39 (7.36)				7
					MS	15	11 (73)	34 (4.61)				
Tajfirouz et al. 2022[56]	Orbits	Acute	Retrospective	USA	NMOSD	74	63 (85)		43.25 (14.85)			7
				Thailand	MOGAD	80	50 (63)		36.25 (15.92)			
Tantsis et al. 2019[57]	Spine	Acute	Retrospective	Australia	NMOSD	9	8 (89)		11.958 (0.95)			8
	Brain				MOGAD	10	6 (60)		10.668 (2.73)			
					MS	13	8 (62)		14.375 (1.50)			
Tzanetakos et al. 2022[58]	Brain	Unknown	Prospective		MOGAD	11	8 (73)	43.62 (25.45)		41.00 (45.38)	1 [0-1]	6
					MS	12	6 (50)	38.9 (6.29)		73.56 (68.98)	1.5 [1-3]	
Xiao et al. 2022[59]	Orbits	All	Retrospective		NMOSD	11	1 (9)		37.2 (13.8)		2 (1-6.5)	7
	Spine				MOGAD	17	12 (71)		32.2 (12.3)		1.5 (1-9.5)	
	Brain											
Xie et al. 2021[60]	Orbits	Acute	Retrospective	China	NMOSD	118	77 (65)	44.64 (14.83)			3 [2-6]	7
	Spine		Cohort		MOGAD	25	10 (40)	35.84 (14.7)			3 [2-4]	
	Brain											
Yang et al. 2020[61]	Spine	Unknown	Retrospective		NMOSD	52	47 (90)	40.25 (12.18)		116.05 (92.94)		7
					MOGAD	24	11 (46)	32.5 (14.38)		50.725 (32.68)		
Yonezu et al. 2014[62]	Spine	Relapse	Retrospective	Japan	NMOSD	24	22 (92)	54.1 (12.2)	41.8 (15.2)			7
					MS	34	28 (82)	38.9 (9.6)	27.6 (8.1)			
ZhangBao et al. 2021[63]	Spine	Unknown	Retrospective		NMOSD	125	109 (87)		38.62 (14.65)	45.77 (39.33)		7
					MOGAD	130	62 (48)		28.89 (16.22)	47.45 (45.77)		
Zhao et al. 2018[64]	Orbits	Acute	Retrospective	China	NMOSD	45	42 (93)	39.7 (15.2)	35.6 (15.7)			8

Author[ref]	Orbits/ Spine/ Brain	Phase	Study Design	Location	Cohort	Sample size	Female n (%)	Age at Time of Study (yrs) Mean (SD)	Age of Onset (yrs) Mean (SD)	Duration (months) of Disease Mean (SD)	EDSS*	Quality Score
	Spine				MOGAD	20	14 (70)	22 (17.5)	20.2 (17.4)			
Zheng et al. 2021[65]	Brain	Relapse + chronic	Retrospective		NMOSD	236	208 (88)	41.09 (13.21)	36.87 (13.16)	50.88 (59.34)		7
					MS	236	151 (64)	36.97 (11.35)	33.75 (11.62)	38.83 (51.16)		
					HC	280	160 (57)	38.9 (12.35)				
Zrzavy et al. 2022[66]	Orbits	Acute + F/U	Retrospective	Austria	NMOSD	10	8 (80)		32.5 (19.8)			8
	Spine				MS	25	20 (80)		28.9 (9.8)			
	Brain											

*EDSS is represented as either mean+/-SD, median (range) or median [IQR]

SD = standard deviation; yrs = years; EDSS = Expanded Disability Status Scale; MOGAD = myelin oligodendrocyte glycoprotein antibody associated disease; NMOSD = neuromyelitis optica spectrum disorder; MS = multiple sclerosis; HC = healthy controls

Supplementary Table 3. Study number and total sample size for network meta-analysed variables

Lesion type	No. of studies	Combined sample size			
		NMOSD	MOGAD	MS	HC
**Optic chiasm T2 lesions**	19	787	383	405	
**Bilateral optic nerve T2 lesions**	13	473	202	305	
**Juxtacortical T2 lesions**	13	732	319	677	
**Subcortical white matter lesions**	12	678	403	377	30
**Corpus callosum T2 lesions**	12	499	346	477	
**Deep grey matter T2 lesions**	19	822	533	425	
**Periventricular T2 lesions**	17	749	289	658	
**Cerebellar T2 lesions**	13	768	418	348	
**Cervical spine T2 lesions**	17	841	285	409	30
**LETM**	22	1105	486	65	
**Conus medullaris T2 lesions**	11	581	316	959	

No. = Number; NMOSD = Neuromyelitis spectrum disorder; MOGAD = myelin oligodendrocyte glycoprotein associated disease; MS = multiple sclerosis; HC = healthy controls; LETM = longitudinally extensive transverse myelitis.

Supplementary table 4. Frequency data, sensitivity and specificity results, and odds ratios with 95% confidence intervals.

Lesion	NMOSD n/N	NMOSD Sens.	MOGAD n/N	MOGAD Sens.	MS n/N	MS Sens.	MS vs NMOSD OR (95% CI)	MS Spec.	NMOSD Spec.	MS vs MOGAD OR (95% CI)	MS Spec.	MOGAD Spec.	NMOSD vs MOGAD OR (95% CI)	NMOSD Spec.	MOGAD Spec.
Orbits															
**Bilateral optic nerve T2 lesions**	137/473	0.290	81/202	0.401	6/305	0.020	0.05 (0.02-0.11)	0.710	0.980	0.03 (0.01-0.07)	0.599	0.980	0.61 (0.43-0.86)	0.599	0.710
**Canalicular optic nerve T2 lesion**	226/410	0.551	73/170	0.429	93/212	0.439	0.64 (0.46-0.89)	0.449	0.561	1.04 (0.69-1.56)	0.571	0.561	1.63 (1.14-2.34)	0.571	0.449
**Gd-enhancing optic nerve lesion**	128/229	0.559	111/157	0.707	112/319	0.351	0.43 (0.30-0.60)	0.441	0.649	0.22 (0.15-0.34)	0.293	0.649	0.53 (0.34-0.81)	0.293	0.441
**Intracranial optic nerve T2 lesion**	91/165	0.552	41/99	0.414	28/106	0.264	0.29 (0.17-0.50)	0.448	0.736	0.51 (0.28-0.91)	0.586	0.736	1.74 (1.05-2.88)	0.586	0.448
**Intraorbital optic nerve T2 lesion**	209/299	0.699	76/103	0.738	58/81	0.716	1.09 (0.63-1.87)	0.301	0.284	0.90 (0.47-1.72)	0.262	0.284	0.83 (0.50-1.37)	0.262	0.301
**Long segment optic nerve T2 lesion**	144/369	0.390	56/115	0.487	30/145	0.207	0.41 (0.26-0.64)	0.610	0.793	0.27 (0.16-0.47)	0.513	0.793	0.67 (0.44-1.03)	0.513	0.610
**Optic Chiasm T2 lesion**	212/787	0.269	34/383	0.089	12/405	0.030	0.08 (0.05-0.15)	0.731	0.970	0.31 (0.16-0.61)	0.911	0.970	3.78 (2.57-5.57)	0.911	0.731
**Optic nerve swelling**	38/67	0.567	30/44	0.667	27/83	0.325	0.37 (0.19-0.72	0.433	0.675	0.23 (0.10-0.49)	0.318	0.675	0.61 (0.28-1.36)	0.333	0.839

	NMOSD		MOGAD		MS		MS vs NMOSD			MS vs MOGAD			NMOSD vs MOGAD		
**Lesion**	**n/N**	**Sens.**	**n/N**	**Sens.**	**n/N**	**Sens.**	**OR (95% CI)**	**MS Spec.**	**NMOSD Spec.**	**OR (95% CI)**	**MS Spec.**	**MOGAD Spec.**	**OR (95% CI)**	**NMOSD Spec.**	**MOGAD Spec.**
**Optic nerve head swelling**	9/56	0.100	26/39	0.682									0.10 (0.04-0.25)	0.318	0.433
**Optic tract T2 lesion**	23/231	0.176	4/137	0.029	2/32	0.034	0.60 (0.14-2.69)	0.900	0.938	2.22 (0.39-12.67)	0.971	0.938	3.68 (1.24-10.87)	0.971	0.900
**Perineural enhancement**	21/119	0.401	40/86	0.465	4/117	0.635	0.17 (0.05-0.50)	0.824	0.966	0.04 (0.01-0.12)	0.535	0.966	0.25 (0.13-0.46)	0.535	0.824
**Unilateral optic nerve T2 lesion**	174/434	0.401	50/151	0.331	54/85	0.635	2.60 (1.61-4.21)	0.599	0.365	3.52 (2.02-6.14)	0.669	0.365	1.35 (0.92-2.0)	0.669	0.599
Brain															
**Adjacent to 3rd ventricle T2 lesion**	22/196	0.112	9/57	0.158	5/262	0.019	0.15 (0.06-0.41)	0.888	0.981	0.10 (0.03-0.32)	0.842	0.981	0.67 (0.29-1.56)	0.842	0.888
**Adjacent to 4th ventricle T2 lesion**	40/199	0.201	21/66	0.318	38/199	0.191	0.94 (0.57-1.54)	0.799	0.809	0.51 (0.27-0.95)	0.682	0.809	0.54 (0.29-1.01)	0.682	0.799
**Adjacent to lateral ventricle T2 lesion**	108/452	0.239	16/82	0.195	8/273	0.029	0.10 (0.05-0.20)	0.761	0.971	0.12 (0.05-0.30)	0.805	0.971	1.30 (0.72-2.33)	0.805	0.761
**Area postrema T2 lesion**	53/253	0.209	5/116	0.043	18/302	0.060	0.24 (0.14-0.42)	0.791	0.940	1.41 (0.51-3.88)	0.957	0.940	5.88 (2.28-15.15)	0.957	0.791

Lesion	NMOSD n/N	NMOSD Sens.	MOGAD n/N	MOGAD Sens.	MS n/N	MS Sens.	MS vs NMOSD OR (95% CI)	MS vs NMOSD MS Spec.	MS vs NMOSD NMOSD Spec.	MS vs MOGAD OR (95% CI)	MS vs MOGAD MS Spec.	MS vs MOGAD MOGAD Spec.	NMOSD vs MOGAD OR (95% CI)	NMOSD vs MOGAD NMOSD Spec.	NMOSD vs MOGAD MOGAD Spec.
**Bilateral hemispheric T2 lesions**	38/58	0.655	32/49	0.653	98/101	0.970	17.19 (4.83-61.22)	0.345	0.030	17.35 (4.77-63.08)	0.347	0.030	1.01 (0.45-2.25)	0.347	0.345
**Brain T1 hypointensity**	37/141	0.262	21/101	0.208	203/260	0.781	10.01 (6.22-16.12)	0.738	0.219	13.57 (7.72-23.83)	0.792	0.219	1.36 (0.74-2.49)	0.792	0.738
**Cerebellar peduncle T2 lesion**	15/154	0.097	31/100	0.310	62/223	0.278	3.57 (1.94-6.55)	0.903	0.722	0.86 (0.51-1.43)	0.690	0.722	0.24 (0.12-0.47)	0.690	0.903
**Cerebellar T2 lesion**	54/768	0.070	46/418	0.110	97/348	0.279	5.11 (3.56-7.34)	0.930	0.721	3.13 (2.13-4.60)	0.890	0.721	0.61 (0.40-0.92)	0.890	0.930
**Cloud-like enhancement**	27/190	0.142			3/273	0.011	0.07 (0.02-0.22)	0.858	0.989						
**Corpus callosum T2 lesion**	100/499	0.200	52/346	0.150	267/477	0.560	5.07 (3.82-6.74)	0.800	0.440	7.19 (5.09-10.16)	0.850	0.440	1.42 (0.98-2.05)	0.850	0.800
**Cortical T2 lesion**	31/344	0.090	23/110	0.209	146/332	0.440	7.93 (5.17-12.16)	0.910	0.560	2.97 (1.79-4.94)	0.791	0.560	0.37 (0.21-0.68)	0.791	0.910
**Dawson's fingers**	30/761	0.039	13/261	0.050	376/627	0.600	36.50 (24.50-54.37)	0.961	0.400	28.58 (16.0-51.05)	0.950	0.400	0.78 (0.40-1.52)	0.950	0.961
**Deep grey matter T2 lesion**	74/822	0.090	80/533	0.150	68/425	0.160	1.93 (1.35-2.74)	0.910	0.840	1.08 (0.76-1.53)	0.850	0.840	0.56 (0.40-0.78)	0.850	0.910
**Fluffy T2 lesion**	13/58	0.224	37/49	0.755	9/98	0.092	0.35 (0.14-0.88)	0.776	0.908	0.03 (0.01-0.08)	0.245	0.908	0.09 (0.04-0.23)	0.245	0.776
**Gd-enhancement brain**	87/272	0.320	51/155	0.329	193/371	0.520	2.31 (1.66-3.19)	0.680	0.480	2.21 (1.49-3.27)	0.671	0.480	0.96 (0.63-1.46)	0.671	0.680

	NMOSD		MOGAD		MS		MS vs NMOSD			MS vs MOGAD			NMOSD vs MOGAD		
**Lesion**	**n/N**	**Sens.**	**n/N**	**Sens.**	**n/N**	**Sens.**	**OR (95% CI)**	**MS Spec.**	**NMOSD Spec.**	**OR (95% CI)**	**MS Spec.**	**MOGAD Spec.**	**OR (95% CI)**	**NMOSD Spec.**	**MOGAD Spec.**
**Hypothalamus T2 lesion**	47/260	0.181	6/84	0.071	10/342	0.029	0.14 (0.07-0.28)	0.819	0.971	0.39 (0.14-1.11)	0.929	0.971	2.87 (1.18-4.97)	0.929	0.819
**Inferior temporal T2 lesion**	49/487	0.101	25/210	0.119	306/566	0.541	10.52 (7.50-14.76)	0.899	0.459	8.71 (5.56-13.65)	0.881	0.459	0.83 (0.50-1.38)	0.881	0.899
**Infratentorial T2 lesion**	46/164	0.280	53/137	0.387	90/274	0.328	1.25 (0.82-1.92)	0.720	0.672	0.78 (0.51-1.19)	0.613	0.672	0.62 (0.38-1.0)	0.613	0.720
**Juxtacortical T2 lesion**	176/732	0.240	89/319	0.279	487/677	0.719	8.10 (6.38-10.28)	0.760	0.281	6.62 (4.92-8.91)	0.721	0.281	0.82 (0.61-1.10)	0.721	0.760
**Large hemispheric T2 lesion**	48/317	0.151	34/129	0.264	52/291	0.179	1.22 (0.79-1.87)	0.849	0.821	0.61 (0.37-1.0)	0.736	0.821	0.50 (0.30-0.82)	0.736	0.849
**Leptomeningeal enhancement**	11/162	0.068			2/188	0.011	0.15 (0.03-0.68)	0.932	0.989						
**Medulla T2 lesion**	83/396	0.210	13/180	0.072	47/168	0.280	1.46 (0.97-2.22)	0.790	0.720	4.99 (2.59-9.63)	0.928	0.720	3.41 (1.84-6.29)	0.928	0.790
**Midbrain T2 lesion**	35/354	0.099	25/169	0.148	32/187	0.171	1.88 (1.12-3.15)	0.901	0.829	1.19 (0.67-2.10)	0.852	0.829	0.63 (0.36-1.10)	0.852	0.901
**Periaqueductal grey matter T2 lesion**	52/328	0.159	13/84	0.155	35/268	0.131	0.80 (0.50-1.27)	0.841	0.869	0.82 (0.41-1.64)	0.845	0.869	1.03 (0.53-1.99)	0.845	0.841
**Periventricular T2 lesion**	300/749	0.401	104/289	0.360	572/658	0.869	9.95 (7.60-13.04)	0.599	0.131	11.83 (8.50-16.46)	0.640	0.131	1.19 (0.90-1.57)	0.640	0.599

	NMOSD		MOGAD		MS		MS vs NMOSD			MS vs MOGAD			NMOSD vs MOGAD		
**Lesion**	**n/N**	**Sens.**	**n/N**	**Sens.**	**n/N**	**Sens.**	**OR (95% CI)**	**MS Spec.**	**NMOSD Spec.**	**OR (95% CI)**	**MS Spec.**	**MOGAD Spec.**	**OR (95% CI)**	**NMOSD Spec.**	**MOGAD Spec.**
**Pons T2 lesion**	47/389	0.121	50/217	0.230	64/187	0.342	3.79 (2.46-5.82)	0.879	0.658	1.74 (1.12-2.69)	0.770	0.658	0.46 (0.30-0.71)	0.770	0.879
**Ring enhancing lesion**	2/96	0.021			39/118	0.331	23.20 (5.43-99.13)	0.979	0.669						
**Subcortical white matter T2 lesion**	346/678	0.510	181/403	0.449	347/377	0.920	11.10 (7.42-16.59)	0.490	0.080	14.19 (9.31-21.62)	0.551	0.080	1.28 (1.0-1.64)	0.551	0.490
**Thalamus T2 lesion**	17/343	0.050	25/154	0.162	35/233	0.150	3.39 (1.85-6.21)	0.950	0.850	0.91 (0.52-1.60)	0.838	0.850	0.27 (0.14-0.51)	0.838	0.950
**Tumefactive T2 lesions**	5/167	0.030			2/152	0.013	0.43 (0.08-2.26)	0.970	0.987						
**U-fibre lesion T2 lesion**	25/411	0.061	6/202	0.030	120/317	0.379	9.41 (5.91-14.95)	0.939	0.621	19.90 (8.56-46.25)	0.970	0.621	2.12 (0.85-5.24)	0.970	0.939
Spine															
**Abnormal MRI spine**	451/654	0.690	103/265	0.389	213/368	0.579	0.62 (0.47-0.81)	0.310	0.421	2.16 (1.57-2.98)	0.611	0.421	3.49 (2.60-4.70)	0.611	0.310
**Anterior spinal column T2 lesion**	6/47	0.128	6/65	0.092	3/67	0.045	0.32 (0.08-1.35)	0.872	0.955	0.46 (0.11-1.93)	0.908	0.955	1.44 (0.43-4.78)	0.908	0.872
**Bright spotty spinal T2 lesion**	117/272	0.430	85/90	0.944	5/263	0.019	0.03 (0.01-0.06)	0.570	0.981	0.00 (0.00-0.00)	0.056	0.981	0.04 (0.02-0.11)	0.056	0.570
**Central spinal cord T2 lesion**	368/613	0.600	124/259	0.479	45/282	0.160	0.13 (0.09-0.18)	0.400	0.840	0.21 (0.14-0.31)	0.521	0.840	1.64 (1.22-2.19)	0.521	0.400

	NMOSD		MOGAD		MS		MS vs NMOSD			MS vs MOGAD			NMOSD vs MOGAD		
**Lesion**	**n/N**	**Sens.**	**n/N**	**Sens.**	**n/N**	**Sens.**	**OR (95% CI)**	**MS Spec.**	**NMOSD Spec.**	**OR (95% CI)**	**MS Spec.**	**MOGAD Spec.**	**OR (95% CI)**	**NMOSD Spec.**	**MOGAD Spec.**
**Cervical spinal cord T2 lesion**	404/841	0.480	91/285	0.319	262/409	0.641	1.93 (1.51-2.46)	0.520	0.359	3.80 (2.76-5.24)	0.681	0.359	1.97 (1.48-2.62)	0.681	0.520
**Conus medullaris T2 lesion**	38/752	0.050	131/396	0.291	21/1037	0.020	0.39 (0.23-0.67)	0.950	0.980	0.04 (0.03-0.07)	0.709	0.980	0.11 (0.07-0.16)	0.709	0.950
**Gd-enhancement spine**	343/520	0.660	106/226	0.469	128/346	0.370	0.30 (0.23-0.40)	0.340	0.630	0.66 (0.47-0.93)	0.531	0.630	2.19 (1.60-3.02)	0.531	0.340
**Heterogenous Gd-enhancement spine**	43/126	0.341			2/78	0.026	0.05 (0.01-0.22)	0.659	0.974						
**Homogenous Gd-enhancement spine**	25/126	0.198			10/76	0.132	0.61 (0.28-1.36)	0.802	0.868						
**LETM**	807/1105	0.730	199/486	0.410	2/65	0.031	0.01 (0.01-0.02)	0.270	0.969	0.05 (0.03-0.07)	0.591	0.969	3.91 (3.12-4.89)	0.591	0.270
**Mixed periph and central spinal T2 lesion**	43/65	0.662	5/27	0.185	26/56	0.464	0.44 (0.21-0.92)	0.338	0.536	3.81 (1.26-11.50)	0.815	0.536	8.60 (2.87-25.80)	0.815	0.338
**Peripheral spinal cord T2 lesion**	30/144	0.208	5/51	0.098	41/94	0.436	2.94 (1.66-5.21)	0.792	0.564	7.12 (2.59-19.52)	0.902	0.564	2.42 (0.88-6.63)	0.902	0.792
**Posterior column T2 lesion**	3/48	0.063	5/66	0.076	25/67	0.373	8.93(2.51-31.77)	0.938	0.627	7.26 (2.57-20.49)	0.924	0.627	0.81 (0.18-3.58)	0.924	0.938

	NMOSD		MOGAD		MS		MS vs NMOSD			MS vs MOGAD			NMOSD vs MOGAD		
**Lesion**	**n/N**	**Sens.**	**n/N**	**Sens.**	**n/N**	**Sens.**	**OR (95% CI)**	**MS Spec.**	**NMOSD Spec.**	**OR (95% CI)**	**MS Spec.**	**MOGAD Spec.**	**OR (95% CI)**	**NMOSD Spec.**	**MOGAD Spec.**
**Short segment spinal cord T2 lesion**	80/187	0.428	3/43	0.070	96/172	0.558	1.69 (1.11-2.57)	0.572	0.442	16.84 (5.02-56.55)	0.930	0.442	9.97 (2.98-33.38)	0.930	0.572
**Spinal cord atrophy**	36/190	0.189	3/62	0.048	7/144	0.049	0.22 (0.09-0.51)	0.811	0.951	1.00 (0.26-4.02)	0.952	0.951	4.60 (1.36-15.50)	0.952	0.811
**Spinal cord expansion**	117/221	0.529	26/55	0.473	22/167	0.132	0.13 (0.08-0.23)	0.471	0.868	0.17 (0.08-0.34)	0.527	0.868	1.25 (0.69-2.27)	0.527	0.471
**T1 hypointensity spine**	133/241	0.552	42/103	0.408	16/134	0.119	0.11 (0.06-0.20)	0.448	0.881	0.20 (0.10-0.38)	0.592	0.881	1.79 (1.12-2.86)	0.592	0.448
**Thoracic spinal cord T2 lesion**	381/793	0.480	118/257	0.459	97/360	0.269	0.40 (0.30-0.52)	0.520	0.731	0.43 (0.31-0.61)	0.541	0.731	1.09 (0.82-1.44)	0.541	0.520
**Transversly extensive spinal T2 lesion**	233/343	0.679	56/116	0.483	34/137	0.248	0.16 (0.10-0.24)	0.321	0.752	0.35 (0.21-0.60)	0.517	0.752	2.27 (1.48-3.49)	0.517	0.321

Sens = sensitivity; Spec = specificity; OR = odds ratio; CI = confidence interval; MOGAD = myelin oligodendrocyte glycoprotein antibody associated disease; NMOSD = neuromyelitis optica spectrum disorder; MS = multiple sclerosis; Gd = gadolinium

Supplementary table 5. Comparison of MS vs non-MS pooled cohorts including frequency data, sensitivity and specificity, and odds ratio with 95% confidence intervals

	MS	non-MS	Predicting MS		Predicting non-MS		MS vs non-MS
**Lesion**	**n/N**	**n/N**	**Sens.**	**Spec.**	**Sens.**	**Spec.**	**OR (95% CI)**
Orbits							
**Bilateral optic nerve T2 lesion**	6/305	218/675	0.019	0.677	0.323	0.980	0.04 (0.02-0.10)
**Gd-enhancement optic nerve**	112/319	239/386	0.351	0.381	0.619	0.649	0.33 (0.24-0.45)
**Intracranial optic nerve T2 lesion**	28/106	132/264	0.264	0.500	0.500	0.736	0.36 (0.22-0.59)
**Long segment optic nerve T2 lesion**	30/145	200/484	0.207	0.587	0.413	0.793	0.37 (0.24-0.58)
**Optic chiasm T2 lesion**	12/405	246/1170	0.030	0.790	0.210	0.970	0.11 (0.06-0.21)
**Optic nerve swelling**	27/83	68/111	0.325	0.387	0.613	0.675	0.30 (0.17-0.55)
**Perineural enhancement**	4/117	61/205	0.034	0.702	0.298	0.966	0.08 (0.03-0.24)
**Unilateral optic nerve T2 lesion**	54/85	224/585	0.635	0.617	0.383	0.365	2.81 (1.75-4.50)
Brain							
**Adjacent to 3rd ventricle T2 lesion**	5/262	31/253	0.019	0.877	0.122	0.981	0.14 (0.05-0.36)
**Adjacent to lateral ventricle T2 lesion**	8/273	124/534	0.029	0.768	0.232	0.971	0.10 (0.05-0.21)
**Brain T1 hypointensity**	203/260	58/242	0.781	0.760	0.240	0.219	11.30 (7.45-17.13)
**Cerebellar T2 lesion**	97/348	100/1186	0.279	0.916	0.084	0.721	4.20 (3.07-5.73)
**Corpus callosal T2 lesion**	267/477	152/845	0.560	0.820	0.180	0.440	5.80 (4.51-7.46)
**Cortical T2 lesion**	146/332	54/454	0.440	0.881	0.119	0.560	5.81 (4.07-8.31)
**Dawson's fingers**	376/627	43/1022	0.600	0.958	0.042	0.400	34.11 (24.16-48.14)
**Fluffy T2 lesion**	9/98	50/107	0.092	0.533	0.467	0.908	0.12 (0.05-0.25)
**Gd-enhancement brain**	193/371	138/427	0.520	0.677	0.323	0.480	2.27 (1.70-3.03)

Lesion	MS	non-MS	Predicting MS		Predicting non-MS		MS vs non-MS
	n/N	n/N	Sens.	Spec.	Sens.	Spec.	OR (95% CI)
**Inferior temporal T2 lesion**	306/566	74/697	0.541	0.894	0.106	0.459	9.91 (7.40-13.27)
**Juxtacortical T2 lesion**	487/677	265/1051	0.719	0.748	0.252	0.281	7.60 (6.11-9.45)
**Periventricular T2 lesion**	572/658	404/1038	0.869	0.611	0.389	0.131	10.44 (8.06-13.52)
**Pons T2 lesion**	64/187	97/606	0.342	0.840	0.160	0.658	2.73 (1.88-3.96)
**Subcortical white matter T2 lesion**	347/377	527/1081	0.920	0.512	0.488	0.080	12.16 (8.22-17.99)
**U-fibre T2 lesion**	120/317	31/613	0.379	0.949	0.051	0.621	11.44 (7.46-17.52)

Lesion	MS n/N	non-MS n/N	Predicting MS Sens.	Predicting MS Spec.	Predicting non-MS Sens.	Predicting non-MS Spec.	MS vs non-MS OR (95% CI)
Spine							
**Bright spotty spinal cord T2 lesion**	5/263	202/362	0.019	0.442	0.558	0.981	0.02 (0.01-0.04)
**Central spinal cord T2 lesion**	45/282	492/872	0.160	0.436	0.564	0.840	0.15 (0.10-0.21)
**Conus medullaris T2 lesion**	21/1037	169/1148	0.020	0.853	0.147	0.980	0.12 (0.08-0.19)
**LETM**	20/653	1006/1591	0.031	0.368	0.632	0.969	0.02 (0.01-0.03)
**Peripheral spinal cord T2 lesion**	41/94	35/195	0.436	0.821	0.179	0.564	3.54 (2.05-6.12)
**Posterior spinal column T2 lesion**	25/67	8/114	0.373	0.930	0.070	0.627	7.89 (3.30-18.88)
**Spinal cord expansion**	22/167	143/276	0.132	0.482	0.518	0.868	0.14 (0.09-0.23)
**T1 hypointensity spine**	16/134	175/344	0.119	0.491	0.519	0.881	0.13 (0.07-0.23)
**Thoracic spine T2 lesion**	97/360	499/1050	0.269	0.525	0.475	0.731	0.41 (0.31-0.53)
**Transversally extensive spinal cord T2 lesion**	34/137	289/459	0.248	0.370	0.630	0.752	0.19 (0.13-0.30)

MS = multiple sclerosis; Sens = sensitivity; Spec = specificity; OR = odds ratio; CI = confidence interval

Supplementary table 6. Comparison of NMOSD vs non-NMOSD pooled cohort including frequency data, sensitivity and specificity, and odds ratio with 95% confidence intervals

Lesion	NMOSD n/N	Non-NMOSD n	Predicting NMOSD Sens.	Predicting NMOSD Spec.	Predicting non-NMOSD Sens.	Predicting non-NMOSD Spec.	NMOSD vs non-NMOSD OR (95% CI)
Orbits							
**Optic chiasm T2 lesion**	212/787	46/788	0.269	0.942	0.058	0.731	5.95 (4.25-8.33)
**Optic tract T2 lesion**	23/231	6/169	0.100	0.965	0.036	0.900	3.00 (1.20-7.55)
Brain							
**Area postrema T2 lesion**	53/253	23/418	0.209	0.945	0.055	0.791	4.55 (2.71-7.64)
**Cerebellar peduncle T2 lesion**	15/154	93/323	0.097	0.712	0.288	0.903	0.27 (0.15-0.48)
**Cerebellum T2 lesion**	54/768	169/442	0.070	0.618	0.382	0.930	0.12 (0.09-0.17)
**Cortical T2 lesion**	31/344	60/387	0.090	0.845	0.155	0.910	0.54 (0.34-0.86)
**Deep grey matter T2 lesion**	74/822	57/356	0.090	0.840	0.160	0.910	0.52 (0.36-0.75)
**Hypothalamus T2 lesion**	47/260	16/426	0.181	0.962	0.038	0.819	5.65 (3.13-10.21)
**Thalamus T2 lesion**	17/343	93/323	0.050	0.712	0.288	0.950	0.13 (0.07-0.22)
Spine							
**LETM**	807/1105	219/1139	0.730	0.808	0.192	0.270	11.38 (9.33-13.87)
**Spinal cord Atrophy**	36/190	10/206	0.189	0.951	0.049	0.811	4.58 (2.20-9.52)

NMOSD = neuromyelitis spectrum disorder; Sens = sensitivity; Spec = specificity; OR = odds ratio; CI = confidence interval

Supplementary table 7. Comparison of MOGAD vs non-MOGAD pooled cohort including frequency data, sensitivity and specificity, and odds ratio with 95% confidence intervals

	MOGAD	Non-MOGAD	Predicting MOGAD		Predicting non-MOGAD		MOGAD vs non-MOGAD
**Lesion**	**n/N**	**n/N**	**Sens.**	**Spec.**	**Sens.**	**Spec.**	**OR (95% CI)**
Orbits							
**Perineural Gd-enhancement**	40/86	25/236	0.465	0.894	0.106	0.535	7.34 (4.06-13.28)
Brain							
**Adjacent to 3rd ventricle T2 lesion**	9/57	27/458	0.158	0.941	0.059	0.842	2.99 (1.33-6.74)
**Adjacent to 4th ventricle T2 lesion**	21/66	78/398	0.318	0.804	0.196	0.682	1.91 (1.08-3.40)
**Fluffy T2 lesion**	37/49	22/156	0.755	0.859	0.141	0.245	18.78 (8.51-41.46)
**Medulla T2 lesion**	13/180	130/564	0.072	0.769	0.231	0.928	0.26 (0.14-0.47)
Spine							
**Abnormal spinal MRI**	103/265	664/1022	0.389	0.350	0.650	0.611	0.34 (0.26-0.45)
**Bright spotty spinal cord T2 lesion**	85/90	122/535	0.944	0.772	0.228	0.056	57.55 (22.84-145.03)
**Cervical spine T2 lesion**	91/285	666/1250	0.319	0.467	0.533	0.681	0.41 (0.31-0.54)
**Conus medullaris T2 lesion**	131/396	59/1789	0.331	0.967	0.033	0.669	14.50 (10.39-20.23)
**Mixed peripheral and central cord T2 lesion**	5/27	69/121	0.185	0.430	0.570	0.815	0.17 (0.06-0.48)
**Short segment spinal cord T2 lesion**	3/43	176/359	0.070	0.510	0.490	0.930	0.08 (0.02-0.26)

MOGAD = myelin oligodendrocyte glycoprotein antibody associated disease; Sens = sensitivity; Spec = specificity; OR = odds ratio; CI = confidence interval; Gd = gadolinium

Supplementary Table 8. Heterogeneity data for individual variable meta-analyses

Lesion Type	Group	No. Studies	Est prop	(95% CI)	I^2	Tau^2	Q	df	p value
**Orbits**									
**Optic chiasm T2 lesion**	NMOSD	20	0.27	(0.19-0.36)	91.79	0.03	246.13	19	<0.001
	MOGAD	16	0.09	(0.06-0.12)	28.68	0.00	18.85	15	0.221
	MS	11	0.03	(0.00-0.05)	57.04	0.00	22.08	10	0.015
**bilateral optic nerve T2 lesion**	NMOSD	12	0.29	(0.15-0.43)	93.65	0.05	167.56	11	<0.001
	MOGAD	11	0.40	(0.27-0.53)	78.62	0.04	53.35	10	<0.001
	MS	8	0.02	(0.00-0.04)	27.87	0.00	11.27	7	0.127
**Long segment optic nerve T2 lesion**	NMOSD	7	0.39	(0.14-0.63)	97.79	0.10	359.80	6	<0.001
	MOGAD	6	0.49	(0.20-0.78)	95.14	0.12	150.19	5	<0.001
	MS	4	0.21	(0.00-0.43)	87.34	0.04	23.65	3	<0.001
**Canalicular T2 lesion**	NMOSD	10	0.55	(0.38-0.72)	93.00	0.07	178.85	9	<0.001
	MOGAD	8	0.43	(0.20-0.65)	93.94	0.10	178.55	7	<0.001
	MS	6	0.44	(0.26-0.62)	85.23	0.04	54.36	5	<0.001
**Optic tract T2 lesion**	NMOSD	7	0.10	(0.02-0.19)	70.47	0.01	19.41	6	0.004
	MOGAD	7	0.03	(0.00-0.06)	0.00	0.00	4.90	6	0.821
	MS	3	0.07	(0.00-0.15)	0.00	0.00	0.98	2	0.613
**Unilateral optic nerve T2 lesion**	NMOSD	8	0.40	(0.22-0.58)	95.55	0.06	251.17	7	<0.001
	MOGAD	8	0.33	(0.19-0.47)	76.88	0.03	44.71	7	<0.001
	MS	3	0.64	(0.20-1.08)	96.41	0.13	90.11	2	<0.001
**Intraorbital T2 lesion**	NMOSD	6	0.70	(0.42-0.98)	98.01	0.12	525.94	5	<0.001
	MOGAD	5	0.74	(0.54-0.95)	88.11	0.05	36.87	4	<0.001
	MS	3	0.71	(0.61-0.81)	4.90	0.00	1.66	2	0.435
**Intracranial T2 lesion**	NMOSD	7	0.55	(0.43-0.67)	61.65	0.02	16.26	6	0.012
	MOGAD	5	0.41	(0.15-0.67)	89.76	0.08	50.74	4	,0.001
	MS	5	0.26	(0.18-0.35)	0.00	0.00	1.90	4	0.754

Lesion Type	Group	No. Studies	Est prop	(95% CI)	I^2	Tau^2	Q	df	p value
**optic nerve swelling**	NMOSD	3	0.56	(0.06-1.06)	97.78	0.19	112.99	2	<0.001
	MOGAD	3	0.68	(0.23-1.12)	95.01	0.15	31.38	2	<0.001
	MS	3	0.32	(0.00-0.67)	93.05	0.09	21.49	2	<0.001
**optic nerve head swelling**	NMOSD	2	0.16	(0.06-0.26)	6.99	0.00	1.08	1	0.3
	MOGAD	2	0.67	(0.40-0.94)	71.80	0.03	3.55	1	0.06
**Orbital Gd enhancement**	NMOSD	9	0.56	(0.33-0.79)	95.74	0.11	232.85	8	<0.001
	MOGAD	7	0.71	(0.52-0.90)	92.62	0.06	78.74	6	<0.001
	MS	7	0.35	(0.10-0.60)	98.20	0.11	271.97	6	<0.001
**Perineural enhancement**	NMOSD	5	0.18	(0.00-0.38)	96.14	0.04	34.53	4	<0.001
	MOGAD	6	0.46	(0.23-0.68)	84.04	0.07	38.36	5	<0.001
	MS	5	0.04	(0.00-0.07)	0.00	0.00	2.72	4	0.606
**Brain**									
**Periventricular T2 lesion**	NMOSD	20	0.40	(0.31-0.48)	0.83	0.03	110.14	19	<0.001
	MOGAD	14	0.36	(0.23-0.48)	86.69	0.05	97.87	13	<0.001
	MS	17	0.87	(0.81-0.92)	0.86	0.01	80.15	16	<0.001
**Deep grey matter T2 lesion**	NMOSD	17	0.09	(0.04-0.13)	81.59	0.00	47.37	16	<0.001
	MOGAD	14	0.15	(0.08-0.22)	83.76	0.01	51.25	13	<0.001
	MS	12	0.16	(0.06-0.26)	95.02	0.03	88.13	11	<0.001
**Juxtacortical T2 lesion**	NMOSD	15	0.24	(0.14-0.34)	95.43	0.03	174.16	14	<0.001
	MOGAD	10	0.28	(0.16-0.40)	82.17	0.02	39.62	9	<0.001
	MS	13	0.72	(0.61-0.83)	92.25	0.04	223.70	12	<0.001
**Cerebellar T2 lesion**	NMOSD	14	0.07	(0.04-0.10)	53.25	0.00	35.20	13	<0.001
	MOGAD	11	0.11	(0.05-0.17)	60.67	0.01	22.71	10	0.012
	MS	10	0.28	(0.22-0.35)	38.86	0.00	17.22	9	0.045
**Subcortical white matter T2 lesion**	NMOSD	13	0.51	(0.37-0.65)	93.28	0.06	433.42	12	<0.001
	MOGAD	9	0.45	(0.27-0.63)	91.14	0.07	109.96	8	<0.001

Lesion Type	Group	No. Studies	Est prop	(95% CI)	I^2	Tau^2	Q	df	p value
	MS	10	0.92	(0.86-0.97)	83.34	0.01	35.83	9	<0.001
**Corpus callosal T2 lesion**	NMOSD	14	0.20	(0.08-0.32)	96.76	0.05	199.39	13	<0.001
	MOGAD	10	0.05	(0.00-0.11)	0.00	0.00	0.90	9	1
	MS	12	0.56	(0.40-0.72)	95.96	0.07	596.46	11	<0.001
**Pons T2 lesion**	NMOSD	13	0.12	(0.07-0.16)	37.42	0.00	22.53	12	0.032
	MOGAD	12	0.23	(0.11-0.34)	84.85	0.03	45.81	11	<0.001
	MS	7	0.35	(0.25-0.44)	49.31	0.01	12.08	6	0.06
**Medulla T2 lesion**	NMOSD	12	0.21	(0.14-0.28)	63.51	0.01	28.51	11	0.003
	MOGAD	10	0.07	(0.03-0.12)	40.69	0.00	17.19	9	0.046
	MS	7	0.28	(0.18-0.39)	56.33	0.01	14.17	6	0.028
**Dawson's fingers T2 lesion**	NMOSD	12	0.04	(0.01-0.07)	87.70	0.00	34.76	11	<0.001
	MOGAD	7	0.05	(0.01-0.09)	38.47	0.00	8.14	6	0.228
	MS	11	0.60	(0.48-0.71)	88.18	0.03	128.19	10	<0.001
**Cortical T2 lesion**	NMOSD	10	0.09	(0.03-0.15)	84.96	0.01	40.92	9	<0.001
	MOGAD	7	0.22	(0.09-0.34)	69.30	0.02	19.51	6	0.003
	MS	9	0.45	(0.26-0.63)	94.02	0.08	130.30	8	<0.001
**Inferior temporal T2 lesion**	NMOSD	10	0.10	(0.06-0.14)	49.32	0.00	17.35	9	0.044
	MOGAD	5	0.12	(0.05-0.20)	32.43	0.00	5.81	4	0.214
	MS	10	0.54	(0.41-0.66)	88.59	0.03	79.91	9	<0.001
**Thalamus T2 lesion**	NMOSD	10	0.05	(0.02-0.07)	7.23	0.00	8.74	9	0.461
	MOGAD	9	0.16	(0.08-0.25)	58.94	0.01	18.78	8	0.016
	MS	7	0.15	(0.04-0.26)	88.78	0.02	24.60	6	<0.001
**Hypothalamus T2 lesion**	NMOSD	10	0.18	(0.09-0.27)	83.21	0.02	41.19	9	<0.001
	MOGAD	6	0.07	(0.01-0.14)	26.80	0.00	6.69	5	0.245
	MS	10	0.04	(0.01-0.06)	20.42	0.00	13.52	9	0.14
**Midbrain T2 lesion**	NMOSD	10	0.10	(0.04-0.16)	77.89	0.01	32.67	9	<0.001
	MOGAD	9	0.15	(0.04-0.25)	89.09	0.02	30.19	8	<0.001

Lesion Type	Group	No. Studies	Est prop	(95% CI)	I^2	Tau^2	Q	df	p value
**Periaqueductal grey matter T2 lesion**	MS	7	0.17	(0.11-0.23)	13.97	0.00	7.58	6	0.27
	NMOSD	10	0.16	(0.10-0.23)	64.67	0.01	30.68	9	<0.001
	MOGAD	6	0.16	(0.03-0.29)	76.78	0.02	17.45	5	0.004
**Area postrema T2 lesion**	MS	10	0.13	(0.05-0.21)	91.16	0.01	49.05	9	<0.001
	NMOSD	10	0.21	(0.09-0.32)	88.43	0.02	59.41	9	<0.001
	MOGAD	7	0.04	(0.01-0.07)	0.07	0.00	8.32	6	0.215
**Large hemispheric T2 lesions**	MS	8	0.06	(0.00-0.13)	88.80	0.01	28.02	7	<0.001
	NMOSD	9	0.15	(0.00-0.30)	96.64	0.05	44.73	8	<0.001
	MOGAD	7	0.26	(0.08-0.44)	88.70	0.05	33.72	6	<0.001
**Peri-ependymal 4th ventricle T2 lesion**	MS	8	0.18	(0.07-0.30)	92.82	0.02	58.26	7	<0.001
	NMOSD	8	0.20	(0.80-0.33)	86.27	0.03	42.38	7	<0.001
	MOGAD	6	0.32	(0.14-0.51)	71.60	0.04	18.10	5	0.003
**Peri-ependymal lateral ventricle T2 lesion**	MS	7	0.19	(0.10-0.29)	70.41	0.01	20.64	6	0.002
	NMOSD	8	0.24	(0.14-0.35)	85.19	0.02	63.63	7	<0.001
	MOGAD	5	0.19	(0.07-0.32)	54.00	0.01	8.84	4	0.065
**Peri-ependymal 3rd ventricle T2 lesion**	MS	6	0.38	(0.07-0.70)	99.11	0.15	502.83	5	<0.001
	NMOSD	7	0.11	(0.04-0.17)	44.66	0.00	10.75	6	0.097
	MOGAD	5	0.16	(0.06-0.26)	20.12	0.00	8.56	4	0.073
**Brain T1 hypointensity**	MS	7	0.02	(0.00-0.04)	6.30	0.00	5.94	6	0.43
	NMOSD	7	0.26	(0.07-0.45)	90.43	0.05	42.41	6	<0.001
	MOGAD	6	0.21	(0.06-0.26)	76.65	0.03	27.34	5	<0.001
**Cerebellar peduncles T2 lesion**	MS	7	0.78	(0.64-0.93)	89.50	0.03	83.24	6	<0.001
	NMOSD	7	0.10	(0.03-0.16)	47.04	0.00	14.29	6	0.027
	MOGAD	6	0.31	(0.12-0.50)	83.57	0.04	43.05	5	<0.001
**Infratentorial T2 lesion**	MS	6	0.29	(0.23-0.34)	0.00	0.00	1.44	5	0.92
	NMOSD	5	0.28	(0.16-0.40)	62.77	0.01	12.59	4	0.013
	MOGAD	4	0.39	(0.31-0.47)	0.00	0.00	0.70	3	0.873

Lesion Type	Group	No. Studies	Est prop	(95% CI)	I^2	Tau^2	Q	df	p value
	MS	5	0.33	(0.15-0.52)	92.69	0.04	52.74	4	<0.001
**U-fibre lesion T2 lesion**	NMOSD	5	0.06	(0.00-0.14)	96.80	0.01	16.87	4	0.002
	MOGAD	3	0.03	(0.01-0.05)	0.00	0.00	1.62	2	0.445
	MS	4	0.38	(0.18-0.59)	92.52	0.04	51.00	3	<0.001
**Fluffy lesion**	NMOSD	4	0.23	(0.00-0.49)	90.26	0.06	18.14	3	<0.001
	MOGAD	4	0.75	(0.49-1.00)	84.84	0.06	16.78	3	<0.001
	MS	4	0.09	(0.00-0.17)	48.64	0.00	6.09	3	0.108
**Bilateral hemispheric T2 lesions**	NMOSD	4	0.66	(0.43-0.88)	77.41	0.04	14.85	3	0.002
	MOGAD	4	0.66	(0.44-0.87)	69.64	0.03	10.73	3	0.013
	MS	4	0.97	(0.93-1.00)	0.00	0.00	1.23	3	0.745
**Tumefactive lesion**	NMOSD	3	0.03	(0.00-0.06)	47.06	0.00	4.11	2	0.128
	MS	2	0.01	(0.00-0.03)	0.00	0.00	0.00	1	0.982
**Brain Gd enhancement**	NMOSD	10	0.32	(0.19-0.45)	86.64	0.04	57.95	9	<0.001
	MOGAD	6	0.33	(0.13-0.53)	91.47	0.05	70.41	5	<0.001
	MS	9	0.52	(0.34-0.71)	94.93	0.07	239.26	8	<0.001
**Cloud-like enhancement**	NMOSD	5	0.15	(0.02-0.27)	88.98	0.02	17.34	4	0.002
	MS	5	0.01	(0.00-0.02)	0.00	0.00	3.59	4	0.465
**Leptomeningeal enhancement**	NMOSD	5	0.07	(0.02-0.11)	21.11	0.00	3.68	4	0.45
	MOGAD	2	0.30	(0.15-0.44)	0.00	0.00	0.00	1	0.969
	MS	4	0.01	(0.00-0.02)	0.00	0.00	0.55	3	0.909
**Ring enhancement**	NMOSD	3	0.02	(0.00-0.05)	0.00	0.00	0.75	2	0.686
	MS	3	0.34	(0.17-0.50)	75.01	0.02	8.28	2	0.016
**Spine**									
**Abnormal spinal MRI**	NMOSD	20	0.69	(0.60-0.77)	85.26	0.03	137.94	19	<0.001
	MOGAD	16	0.39	(0.25-0.52)	85.53	0.06	137.75	15	<0.001
	MS	11	0.58	(0.44-0.72)	89.03	0.05	95.29	10	<0.001
**LETM**	NMOSD	22	0.73	(0.66-0.80)	88.01	0.02	221.52	21	<0.001
	MOGAD	16	0.41	(0.28-0.54)	93.60	0.06	422.82	15	<0.001

Lesion Type	Group	No. Studies	Est prop	(95% CI)	I^2	Tau^2	Q	df	p value
	MS	14	0.03	(0.02-0.05)	14.36	0.00	19.77	13	0.101
**Cervical spine T2 lesion**	NMOSD	16	0.48	(0.37-0.58)	90.04	0.04	180.31	15	<0.001
	MOGAD	12	0.32	(0.17-0.48)	93.07	0.07	150.49	11	<0.001
	MS	11	0.64	(0.51-0.76)	87.77	0.04	118.15	10	<0.001
**Thoracic spine T2 lesion**	NMOSD	14	0.48	(0.38-0.57)	85.95	0.03	83.57	13	<0.001
	MOGAD	11	0.46	(0.28-0.64)	92.90	0.09	172.00	10	<0.001
	MS	9	0.27	(0.17-0.36)	81.70	0.02	53.49	8	<0.001
**Central cord T2 lesion**	NMOSD	14	0.60	(0.45-0.75)	95.57	0.07	580.00	13	<0.001
	MOGAD	11	0.48	(0.34-0.63)	86.79	0.05	101.90	10	<0.001
	MS	9	0.16	(0.08-0.24)	77.25	0.01	32.53	8	<0.001
**Conus medullaris T2 lesion**	NMOSD	14	0.05	(0.03-0.06)	11.44	0.00	13.51	13	0.409
	MOGAD	15	0.33	(0.21-0.44)	88.11	0.04	107.37	14	<0.001
	MS	8	0.02	(0.00-0.04)	14.706	0.00	16.72	7	0.019
**Bright spotty T2 lesion**	NMOSD	6	0.43	(0.30-0.56)	80.17	0.02	26.85	5	<0.001
	MOGAD	2	0.94	(0.83-1.05)	77.83	0.01	4.51	1	0.034
	MS	6	0.02	(0.00-0.04)	0.00	0.00	2.23	5	0.816
**T1 hypointensity spine**	NMOSD	6	0.55	(0.30-0.80)	96.17	0.09	261.14	5	<0.001
	MOGAD	4	0.31	(0.09-0.73)	94.36	0.09	101.38	3	<0.001
	MS	4	0.13	(0.01-0.24)	69.92	0.01	12.65	3	0.005
**Spinal cord expansion**	NMOSD	6	0.53	(0.31-0.74)	92.67	0.07	80.56	5	<0.001
	MOGAD	3	0.48	(0.02-0.95)	95.92	0.16	54.26	2	<0.001
	MS	4	0.13	(0.00-0.33)	98.26	0.04	20.58	3	<0.001
**Transversally extensive T2 lesion**	NMOSD	6	0.68	(0.42-0.94)	97.98	0.10	296.88	5	<0.001
	MOGAD	3	0.48	(0.04-0.92)	97.81	0.15	107.86	2	<0.001
	MS	4	0.25	(0.03-0.46)	92.83	0.04	42.95	3	<0.001
**Short segment T2 lesion**	NMOSD	5	0.43	(0.32-0.55)	46.11	0.01	6.90	4	0.141
	MOGAD	3	0.09	(0.00-0.17)	0.00	0.00	1.62	2	0.446

Lesion Type	Group	No. Studies	Est prop	(95% CI)	I^2	Tau^2	Q	df	p value
	MS	4	0.57	(0.35-0.78)	85.42	0.04	14.29	3	0.003
**Peripheral cord T2 lesion**	NMOSD	5	0.21	(0.00-0.47)	97.65	0.08	60.01	4	<0.001
	MOGAD	3	0.10	(0.00-0.21)	48.93	0.01	3.87	2	0.145
	MS	4	0.45	(0.30-0.59)	53.75	0.01	6.41	3	0.093
**Spinal cord atrophy**	NMOSD	5	0.19	(0.05-0.32)	87.37	0.02	34.14	4	<0.001
	MOGAD	3	0.05	(0.00-0.10)	0.00	0.00	0.06	2	0.971
	MS	3	0.05	(0.02-0.09)	0.00	0.00	0.44	2	0.804
**Posterior column T2 lesion**	NMOSD	2	0.06	(0.00-0.16)	53.14	0.00	2.13	1	0.144
	MOGAD	3	0.08	(0.00-0.17)	43.91	0.00	3.52	2	0.172
	MS	2	0.38	(0.02-0.74)	88.16	0.06	8.44	1	0.004
**Anterior column T2 lesion**	NMOSD	2	0.12	(0.03-0.21)	0.00	0.00	0.69	1	0.407
	MOGAD	3	0.09	(0.02-0.15)	0.00	0.00	0.47	2	0.79
	MS	2	0.21	(0.00-0.48)	82.97	0.03	5.87	1	0.015
**Mixed central and peripheral cord T2 lesion**	NMOSD	3	0.66	(0.55-0.78)	0.00	0.00	0.05	2	0.974
	MOGAD	2	0.19	(0.00-0.54)	81.30	0.06	5.35	1	0.021
	MS	3	0.47	(0.22-0.72)	74.81	0.04	8.48	2	0.014
**Gd enhancement spine**	NMOSD	15	0.66	(0.55-0.77)	89.84	0.04	141.33	14	<0.001
	MOGAD	9	0.47	(0.29-0.66)	91.05	0.07	173.09	8	<0.001
	MS	11	0.37	(0.19-0.54)	95.95	0.08	329.89	10	<0.001
**Heterogenous enhancement spine**	NMOSD	3	0.34	(0.19-0.50)	72.23	0.01	6.89	2	0.032
	MS	3	0.03	(0.00-0.06)	0.44	0.00	3.84	2	0.147
**Homogenous enhancement spine**	NMOSD	3	0.20	(0.05-0.36)	82.15	0.02	10.42	2	0.005
	MS	3	0.13	(0.06-0.21)	0.00	0.00	1.20	2	0.549

MOGAD = myelin oligodendrocyte glycoprotein antibody associated disease; NMOSD = neuromyelitis optica spectrum disorder; MS = multiple sclerosis

Q = Cochran's Q; df = degrees of freedom

Supplementary Table 9. Meta regression results for Subcortical White Matter T2 Lesions

	Estimate (95% CI)	z	P value	I^2 (95% CI)	Tau^2 (95% CI)	Q	df	P	Egger's test p value
**Intercept**	1.486 (0.922 – 2.050)	5.166	<0.001	97.78	0.041	1197.171	18	<0.001	0.672
**NMOSD***	-0.304 (-0.563 – -0.046)	-2.307	0.021	(95.616-98.928)	(0.020-0.085)				
**MOGAD***	-0.496 (-0.728 – -0.264)	-4.196	<0.001						
**HC***	-0.739 (-1.185 – -0.292)	-3.239	0.001						
**Age at time of study (years)**	-0.007 (-0.016 – 0.002)	-1.451	0.147						
**Female proportion**	-0.494 (-1.377 – 0.388)	-1.099	0.272						

* MS as comparator

MOGAD = myelin oligodendrocyte glycoprotein antibody associated disease; NMOSD = neuromyelitis optica spectrum disorder; MS = multiple sclerosis

CI = confidence interval; Q = Cochran's Q; df = degrees of freedom

Supplementary Table 10. Definitions of lesions not previously elaborated on in Clarke et al 2021[14]

Lesion characteristic	Definition	References
**Canalicular optic nerve T2 lesion**	T2 hyperintensity of the optic nerve as it passes through the optic canal	(1, 18)
**Intracranial optic nerve T2 lesion**	T2 hyperintensity of the optic nerve posterior to the optic canal and anterior to the optic chiasm	(1, 18)
**Intraorbital optic nerve T2**	T2 hyperintensity of the optic nerve anterior to it entering the optic canal	(1, 18)
**Optic chiasm T2 lesion**	T2 hyperintensity affecting any part of the optic chiasm	(1, 18, 56)
**Optic nerve swelling**	swelling of the optic nerve when compared with the contralateral normal optic nerve in unilateral ON, or an ipsilateral unaffected segment of optic nerve in bilateral ON.	(47)
**Optic nerve head swelling**	optic nerve head elevation, optic nerve sheath enlargement	(47)
**Optic tract T2 lesion**	T2 hyperintensity posterior to the optic chiasm	(1, 18)
**Perineural enhancement**	as an extensive enhancement pattern that was not limited to the optic nerve but extended to the soft tissues of the orbit	(31)
**Fluffy T2 lesion**	a poorly demarcated hyperintensity on T2 imaging	(10, 30)
**Juxtacortical T2 lesion**	A T2-hyperintense cerebral white matter lesion abutting the cortex, and not separated from it by white matter.	(14, 67)
**Ring enhancing brain lesion**	peripheral hyperintense rim surrounding a hypointense center on T1 post-contrast imaging	(26)
**Conus medullaris T2 lesion**	T2 hyperintensity involving the conus medullaris (lower cone-shaped end of the spinal cord)	(22)
**Peripheral spinal cord T2 lesion**	T2 hyperintensity which lays outside of an imaginary eclipse, of which the centre point was at the central canal and the boundary of the eclipse was not beyond the anterior and posterior horns of the gray matter of the spinal cord (H shape)	(20)
**Transversally extensive spinal cord T2 lesion**	T2 hyperintense axial lesions extending over 50% of the spinal cord, as measured by cross-sectional area	(27, 28)

References

1. Akaishi T, Nakashima I, Takeshita T, Kaneko K, Mugikura S, Sato DK, et al. Different etiologies and prognoses of optic neuritis in demyelinating diseases. Journal of Neuroimmunology. 2016;299:152-7.

2. Ambika S, Durgapriyadarshini S, Padmalakshmi K, Noronha V, Arjundas D. Clinical profile, imaging features and short term visual outcomes of Indian optic neuritis patients with and without seromarkers for myelin oligodendrocyte glycoprotein and neuromyelitis optica. Indian journal of ophthalmology. 2022;70(1):194-200.

3. Bensi C, Marrodan M, González A, Chertcoff A, Sanz EO, Chaves H, et al. Brain and spinal cord lesion criteria distinguishes AQP4-positive neuromyelitis optica and MOG-positive disease from multiple sclerosis. Multiple sclerosis and related disorders. 2018;25:246-50.

4. Cacciaguerra L, Meani A, Mesaros S, Radaelli M, Palace J, Dujmovic-Basuroski I, et al. Brain and cord imaging features in neuromyelitis optica spectrum disorders. Annals of Neurology. 2019;85(3):371-84.

5. Cacciaguerra L, Morris P, Tobin WO, Chen JJ, Banks SA, Elsbernd P, et al. Tumefactive Demyelination in MOG Ab–Associated Disease, Multiple Sclerosis, and AQP-4-IgG–Positive Neuromyelitis Optica Spectrum Disorder. Neurology. 2023;100(13):e1418-e32.

6. Cai M-T, Zhang Y-X, Zheng Y, Fang W, Ding M-P. Callosal lesions on magnetic resonance imaging with multiple sclerosis, neuromyelitis optica spectrum disorder and acute disseminated encephalomyelitis. Multiple Sclerosis and Related Disorders. 2019;32:41-5.

7. Cai M-T, Zheng Y, Shen C-H, Yang F, Fang W, Zhang Y-X, et al. Evaluation of brain and spinal cord lesion distribution criteria at disease onset in distinguishing NMOSD

from MS and MOG antibody-associated disorder. Multiple Sclerosis Journal. 2021;27(6):871-82.

8. Camera V, Holm-Mercer L, Ali AAH, Messina S, Horvat T, Kuker W, et al. Frequency of new silent MRI lesions in myelin oligodendrocyte glycoprotein antibody disease and aquaporin-4 antibody neuromyelitis optica spectrum disorder. JAMA Network Open. 2021;4(12):e2137833-e.

9. Carnero Contentti E, López PA, Criniti J, Pettinicchi JP, Cristiano E, Patrucco L, et al. Chiasmatic lesions on conventional magnetic resonance imaging during the first event of optic neuritis in patients with neuromyelitis optica spectrum disorder and myelin oligodendrocyte glycoprotein-associated disease in a Latin American cohort. European Journal of Neurology. 2022;29(3):802-9.

10. Chen B, Qin C, Ji S, Tian D, Zhang M, Bu B. Modified models to distinguish central nervous system demyelinating diseases with brain lesions. Multiple Sclerosis and Related Disorders. 2021;52:102965.

11. Chia NH, Redenbaugh V, Chen JJ, Pittock SJ, Flanagan EP. Corpus callosum involvement in MOG antibody-associated disease in comparison to AQP4-IgG-seropositive neuromyelitis optica spectrum disorder and multiple sclerosis. Multiple Sclerosis Journal. 2023;29(6):748-52.

12. Chien C, Scheel M, Schmitz-Hübsch T, Borisow N, Ruprecht K, Bellmann-Strobl J, et al. Spinal cord lesions and atrophy in NMOSD with AQP4-IgG and MOG-IgG associated autoimmunity. Multiple Sclerosis Journal. 2019;25(14):1926-36.

13. Ciotti JR, Eby NS, Brier MR, Wu GF, Chahin S, Cross AH, et al. Central vein sign and other radiographic features distinguishing myelin oligodendrocyte glycoprotein antibody disease from multiple sclerosis and aquaporin-4 antibody-positive neuromyelitis optica. Multiple Sclerosis Journal. 2022;28(1):49-60.

14. Clarke L, Arnett S, Bukhari W, Khalilidehkordi E, Jimenez Sanchez S, O'Gorman C, et al. MRI patterns distinguish AQP4 antibody positive neuromyelitis optica spectrum disorder from multiple sclerosis. Frontiers in neurology. 2021;12:722237.

15. Combes AJ, Matthews L, Lee JS, Li DK, Carruthers R, Traboulsee AL, et al. Cervical cord myelin water imaging shows degenerative changes over one year in multiple sclerosis but not neuromyelitis optica spectrum disorder. NeuroImage: Clinical. 2017;16:17-22.

16. Cortese R, Battaglini M, Prados F, Bianchi A, Haider L, Jacob A, et al. Clinical and MRI measures to identify non-acute MOG-antibody disease in adults. Brain. 2023;146(6):2489-501.

17. Cortese R, Carrasco FP, Tur C, Bianchi A, Brownlee W, De Angelis F, et al. Differentiating multiple sclerosis from AQP4-neuromyelitis optica spectrum disorder and MOG-antibody disease with imaging. Neurology. 2023;100(3):e308-e23.

18. Darakdjian M, Chaves H, Hernandez J, Cejas C. MRI pattern in acute optic neuritis: Comparing multiple sclerosis, NMO and MOGAD. The Neuroradiology Journal. 2023;36(3):267-72.

19. Dubey D, Pittock SJ, Krecke KN, Morris PP, Sechi E, Zalewski NL, et al. Clinical, radiologic, and prognostic features of myelitis associated with myelin oligodendrocyte glycoprotein autoantibody. JAMA neurology. 2019;76(3):301-9.

20. Dumrikarnlert C, Siritho S, Chulapimphan P, Ngamsombat C, Satukijchai C, Prayoonwiwat N. The characteristics of spinal imaging in different types of demyelinating diseases. Journal of the neurological sciences. 2017;372:138-43.

21. Etemadifar M, Abbasi M, Salari M, Etemadifar F, Tavakoli H. Comparing myelin oligodendrocyte glycoprotein antibody (MOG-Ab) and non MOG-Ab associated optic neuritis: clinical course and treatment outcome. Multiple sclerosis and related disorders. 2019;27:127-30.

22. Etemadifar M, Salari M, Kargaran PK, Sigari AA, Nouri H, Etemadifar F, et al. Conus medullaris involvement in demyelinating disorders of the CNS: a comparative study. Multiple Sclerosis and Related Disorders. 2021;54:103127.

23. Fadda G, Alves CA, O'Mahony J, Castro DA, Yeh EA, Marrie RA, et al. Comparison of spinal cord magnetic resonance imaging features among children with acquired demyelinating syndromes. JAMA network open. 2021;4(10):e2128871-e.

24. Goldman-Yassen A, Lee A, Gombolay G. Leptomeningeal Enhancement in Pediatric Anti-Myelin Oligodendrocyte Glycoprotein Antibody Disease, Multiple Sclerosis, and Neuromyelitis Optica Spectrum Disorder. Pediatric Neurology. 2024;153:125-30.

25. Hacohen Y, Mankad K, Chong WK, Barkhof F, Vincent A, Lim M, et al. Diagnostic algorithm for relapsing acquired demyelinating syndromes in children. Neurology. 2017;89(3):269-78.

26. Huh S-Y, Min J-H, Kim W, Kim S-H, Kim HJ, Kim B-J, et al. The usefulness of brain MRI at onset in the differentiation of multiple sclerosis and seropositive neuromyelitis optica spectrum disorders. Multiple Sclerosis Journal. 2014;20(6):695-704.

27. Hyun J-W, Kim S-H, Jeong IH, Lee SH, Kim HJ. Bright spotty lesions on the spinal cord: an additional MRI indicator of neuromyelitis optica spectrum disorder? Journal of Neurology, Neurosurgery & Psychiatry. 2015;86(11):1280-2.

28. Hyun J-W, Lee HL, Park J, Kim J, Min J-H, Kim BJ, et al. Brighter spotty lesions on spinal MRI help differentiate AQP4 antibody-positive NMOSD from MOGAD. Multiple Sclerosis Journal. 2022;28(6):989-92.

29. Ito S, Mori M, Makino T, Hayakawa S, Kuwabara S. "Cloud-like enhancement" is a magnetic resonance imaging abnormality specific to neuromyelitis optica. Annals of Neurology: Official Journal of the American Neurological Association and the Child Neurology Society. 2009;66(3):425-8.

30. Jurynczyk M, Geraldes R, Probert F, Woodhall MR, Waters P, Tackley G, et al. Distinct brain imaging characteristics of autoantibody-mediated CNS conditions and multiple sclerosis. Brain. 2017;140(3):617-27.

31. Kim S-M, Woodhall MR, Kim J-S, Kim S-J, Park KS, Vincent A, et al. Antibodies to MOG in adults with inflammatory demyelinating disease of the CNS. Neurology-Neuroimmunology Neuroinflammation. 2015;2(6).

32. Kitley J, Waters P, Woodhall M, Leite MI, Murchison A, George J, et al. Neuromyelitis optica spectrum disorders with aquaporin-4 and myelin-oligodendrocyte glycoprotein antibodies: a comparative study. JAMA neurology. 2014;71(3):276-83.

33. Li X, Miao X, Wang Y, Sun J, Gao H, Han J, et al. Central nervous system tumefactive demyelinating lesions: Risk factors of relapse and follow-up observations. Frontiers in Immunology. 2022;13:1052678.

34. Liao M-F, Chang K-H, Lyu R-K, Huang C-C, Chang H-S, Wu Y-R, et al. Comparison between the cranial magnetic resonance imaging features of neuromyelitis optica spectrum disorder versus multiple sclerosis in Taiwanese patients. BMC neurology. 2014;14:1-9.

35. Lin C-W, Chen W-T, Lin Y-H, Hung K, Chen T-C. Clinical characteristics and prognosis of optic neuritis in Taiwan-a hospital-based cohort study. Multiple Sclerosis and Related Disorders. 2023;75:104739.

36. Chen M, Zhang B, Gao C, Zheng Y, Xie L, Gao Q, et al. Brain gadolinium enhancement along the ventricular and leptomeningeal regions in patients with aquaporin-4 antibodies in cerebral spinal fluid. Journal of Neuroimmunology. 2014;269(1-2):62-7.

37. Lu P, Tian G, Liu X, Wang F, Zhang Z, Sha Y. Differentiating neuromyelitis optica–related and multiple sclerosis–related acute optic neuritis using conventional magnetic

resonance imaging combined with readout-segmented echo-planar diffusion-weighted imaging. Journal of Computer Assisted Tomography. 2018;42(4):502-9.

38. Mariano R, Messina S, Kumar K, Kuker W, Leite MI, Palace J. Comparison of clinical outcomes of transverse myelitis among adults with myelin oligodendrocyte glycoprotein antibody vs aquaporin-4 antibody disease. JAMA Network Open. 2019;2(10):e1912732-e.

39. Marrodán M, Hernandez MA, Köhler AA, Correale J. Differential diagnosis in acute inflammatory myelitis. Multiple Sclerosis and Related Disorders. 2020;46:102481.

40. Masuda H, Mori M, Hirano S, Uzawa A, Uchida T, Muto M, et al. Silent progression of brain atrophy in aquaporin-4 antibody-positive neuromyelitis optica spectrum disorder. Journal of Neurology, Neurosurgery & Psychiatry. 2022;93(1):32-40.

41. Matthews L, Kolind S, Brazier A, Leite MI, Brooks J, Traboulsee A, et al. Imaging surrogates of disease activity in neuromyelitis optica allow distinction from multiple sclerosis. PLoS One. 2015;10(9):e0137715.

42. Matthews L, Marasco R, Jenkinson M, Küker W, Luppe S, Leite MI, et al. Distinction of seropositive NMO spectrum disorder and MS brain lesion distribution. Neurology. 2013;80(14):1330-7.

43. Nagireddy RBR, Kumar A, Singh VK, Prasad R, Pathak A, Chaurasia RN, et al. Clinicoradiological comparative study of Aquaporin-4-IgG seropositive neuromyelitis optica spectrum disorder (NMOSD) and MOG antibody associated disease (MOGAD): A prospective observational study and review of literature. Journal of Neuroimmunology. 2021;361:577742.

44. Papadopoulou A, Oertel FC, Gaetano L, Kuchling J, Zimmermann H, Chien C, et al. Attack-related damage of thalamic nuclei in neuromyelitis optica spectrum disorders. Journal of Neurology, Neurosurgery & Psychiatry. 2019;90(10):1156-64.

45. Pekcevik Y, Mitchell CH, Mealy MA, Orman G, Lee IH, Newsome SD, et al. Differentiating neuromyelitis optica from other causes of longitudinally extensive transverse myelitis on spinal magnetic resonance imaging. Multiple Sclerosis Journal. 2016;22(3):302-11.

46. Peng Y, Liu L, Zheng Y, Qiao Z, Feng K, Wang J. Diagnostic implications of MOG/AQP4 antibodies in recurrent optic neuritis. Experimental and Therapeutic Medicine. 2018;16(2):950-8.

47. Ramanathan S, Prelog K, Barnes EH, Tantsis EM, Reddel SW, Henderson AP, et al. Radiological differentiation of optic neuritis with myelin oligodendrocyte glycoprotein antibodies, aquaporin-4 antibodies, and multiple sclerosis. Multiple Sclerosis Journal. 2016;22(4):470-82.

48. Rempe T, Tarhan B, Rodriguez E, Viswanathan VT, Gyang TV, Carlson A, et al. Anti-MOG associated disorder–Clinical and radiological characteristics compared to AQP4-IgG+ NMOSD–A single-center experience. Multiple sclerosis and related disorders. 2021;48:102718.

49. Salama S, Khan M, Shanechi A, Levy M, Izbudak I. MRI differences between MOG antibody disease and AQP4 NMOSD. Multiple Sclerosis Journal. 2020;26(14):1854-65.

50. Salunkhe M, Gupta P, Singh RK, Elavarasi A, Vibha D, Garg A, et al. A comparative analysis of demographic, clinical and imaging features of myelin oligodendrocyte glycoprotein antibody positive, aquaporin 4 antibody positive, and double seronegative demyelinating disorders–An Indian tertiary care center prospective study. Journal of Neurosciences in Rural Practice. 2023;14(2):313.

51. Sato DK, Callegaro D, Lana-Peixoto MA, Waters PJ, de Haidar Jorge FM, Takahashi T, et al. Distinction between MOG antibody-positive and AQP4 antibody-positive NMO spectrum disorders. Neurology. 2014;82(6):474-81.

52. Sechi E, Krecke KN, Messina SA, Buciuc M, Pittock SJ, Chen JJ, et al. Comparison of MRI lesion evolution in different central nervous system demyelinating disorders. Neurology. 2021;97(11):e1097-e109.

53. Siegel DR, Van Harn M, Taguchi M, Bansal P, Cerghet M, Memon AB. Clinical and diagnostic spectrum of optic neuritis: A single-center retrospective study of disorders associated with multiple sclerosis, anti-aquaporin-4 and anti-myelin oligodendrocyte glycoprotein antibodies. Clinical neurology and neurosurgery. 2022;221:107381.

54. Silveira F, Pappolla A, Sánchez F, Marques VD, de Castillo IS, Tkachuk V, et al. Brain magnetic resonance imaging features in multiple sclerosis and neuromyelitis optica spectrum disorders patients with or without aquaporin-4 antibody in a Latin American population. Multiple Sclerosis and Related Disorders. 2020;42:102049.

55. Storoni M, Davagnanam I, Radon M, Siddiqui A, Plant GT. Distinguishing optic neuritis in neuromyelitis optica spectrum disease from multiple sclerosis: a novel magnetic resonance imaging scoring system. Journal of Neuro-Ophthalmology. 2013;33(2):123-7.

56. Tajfirouz D, Padungkiatsagul T, Beres S, Moss HE, Pittock S, Flanagan E, et al. Optic chiasm involvement in AQP-4 antibody–positive NMO and MOG antibody–associated disorder. Multiple Sclerosis Journal. 2022;28(1):149-53.

57. Tantsis EM, Prelog K, Alper G, Benson L, Gorman M, Lim M, et al. Magnetic resonance imaging in enterovirus-71, myelin oligodendrocyte glycoprotein antibody, aquaporin-4 antibody, and multiple sclerosis-associated myelitis in children. Developmental Medicine & Child Neurology. 2019;61(9):1108-16.

58. Tzanetakos D, Tzartos JS, Vakrakou AG, Breza M, Velonakis G, Stathopoulos P, et al. Cortical involvement and leptomeningeal inflammation in myelin oligodendrocyte glycoprotein antibody disease: a three-dimensional fluid-attenuated inversion recovery MRI study. Multiple Sclerosis Journal. 2022;28(5):718-29.

59. Xiao J, Zhang S-Q, Chen X, Tang Y, Chen M, Shang K, et al. Comparison of clinical and radiological characteristics in autoimmune GFAP astrocytopathy, MOGAD and AQP4-IgG+ NMOSD mimicking intracranial infection as the initial manifestation. Multiple Sclerosis and Related Disorders. 2022;66:104057.

60. Xie H, Shao Y, Du J, Song Y, Li Y, Duan R, et al. Comparative analysis of clinical and imaging data between patients with myelin oligodendrocyte glycoprotein antibody disease and patients with aquaporin 4 antibody-positive neuromyelitis optica spectrum disorder. Journal of Neurology. 2022;269(3):1641-50.

61. Yang L, Li H, Xia W, Quan C, Zhou L, Geng D, et al. Quantitative brain lesion distribution may distinguish MOG-ab and AQP4-ab neuromyelitis optica spectrum disorders. European radiology. 2020;30:1470-9.

62. Yonezu T, Ito S, Mori M, Ogawa Y, Makino T, Uzawa A, et al. "Bright spotty lesions" on spinal magnetic resonance imaging differentiate neuromyelitis optica from multiple sclerosis. Multiple Sclerosis Journal. 2014;20(3):331-7.

63. ZhangBao J, Huang W, Zhou L, Wang L, Chang X, Lu C, et al. Myelitis in inflammatory disorders associated with myelin oligodendrocyte glycoprotein antibody and aquaporin-4 antibody: A comparative study in Chinese Han patients. European journal of neurology. 2021;28(4):1308-15.

64. Zhao Y, Tan S, Chan TCY, Xu Q, Zhao J, Teng D, et al. Clinical features of demyelinating optic neuritis with seropositive myelin oligodendrocyte glycoprotein antibody in Chinese patients. British Journal of Ophthalmology. 2018;102(10):1372-7.

65. Zheng F, Li Y, Zhuo Z, Duan Y, Cao G, Tian D, et al. Structural and functional hippocampal alterations in Multiple sclerosis and neuromyelitis optica spectrum disorder. Multiple Sclerosis Journal. 2022;28(5):707-17.

66. Zrzavy T, Leutmezer F, Rommer P, Bsteh G, Kornek B, Berger T, et al. Imaging features to distinguish AQP4-positive NMOSD and MS at disease onset: A retrospective analysis in a single-center cohort. European Journal of Radiology. 2022;146:110063.

67. Thompson AJ, Banwell BL, Barkhof F, Carroll WM, Coetzee T, Comi G, et al. Diagnosis of multiple sclerosis: 2017 revisions of the McDonald criteria. The Lancet Neurology. 2018;17(2):162-73.
